# Palladium-Catalyzed Stereoselective Construction of 1,3-Stereocenters Displaying Axial and Central Chirality via Asymmetric Alkylations

**DOI:** 10.3390/molecules28072927

**Published:** 2023-03-24

**Authors:** Aiqi Xue, Xingfu Wei, Yue Huang, Jingping Qu, Baomin Wang

**Affiliations:** State Key Laboratory of Fine Chemicals, Department of Pharmaceutical Sciences, School of Chemical Engineering, Dalian University of Technology, 2 Linggong Road, Dalian 116024, China

**Keywords:** Pd-catalysis, 1,3-stereocenters, axial and central chirality, asymmetric alkylation

## Abstract

The concurrent construction of 1,3-stereocenters remains a challenge. Herein, we report the development of stereoselective union of a point chiral center with allenyl axial chirality in 1,3-position by Pd-catalyzed asymmetric allenylic alkylation between racemic allenyl carbonates and indanone-derived β-ketoesters. Various target products bearing a broad range of functional groups were afforded in high yield (up to 99%) with excellent enantioselectivities (up to 98% ee) and good diastereoselectivities (up to 13:1 dr).

## 1. Introduction

**Scheme 1:**
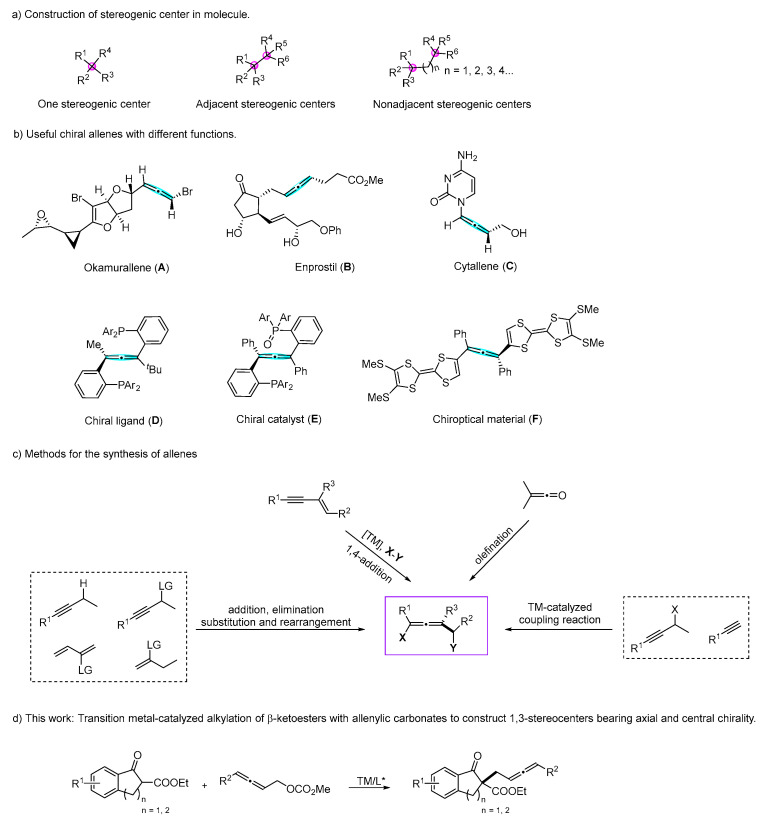
Representative allenes with different functions, different methods for the synthesis of allenes and construction of 1,3-stereocenters bearing allenyl axial and central chirality.

Over the past decades, extensive demands for chiral non-racemic compounds from various fields have significantly motivated the development of asymmetric catalysis [1,2,3,4,5]. Hence numerous catalytic asymmetric methodologies have been developed for enantioselective construction of chiral structures [6,7,8,9]. Whereas many methods are available for the synthesis of chiral molecules containing one single stereocenter or even two adjacent stereocenters (Scheme 1a), much less efforts have been paid to the concurrent creation of nonadjacent stereocenters in an enantio- and diastereoselective manner, due in part to the difficulties in high levels of simultaneous stereocontrol posed by increased distance between the two chiral centers [10,11,12,13,14]. Of note, the limited reports on enantio- and diastereoselective construction of 1,3-stereocenters focused mostly on molecules bearing two point chiral centers, while those containing different types of chirality, for example, central and axial chiral motifs, have been rarely explored.

As a prominent example of axial chiral molecules, chiral allenes constitute an important structural motif widely present in a variety of organic molecules including natural products, pharmaceutical agents, chiral catalysts, and ligands for coordination chemistry etc., as represented by the selected compounds in Scheme 1b [15,16,17,18,19]. Interestingly, Enprostil [20], a prostaglandin analogue used for the treatment of acute duodenal ulcer disease, shows a distinct 1,3-stereocenters consisting of a point chiral center and allenyl axial chirality. 

Functionalized alkynes are most commonly employed for the synthesis of chiral allenes by classic synthetic methods such as addition, elimination, substitution, and rearrangement (Scheme 1c) [5,21,22,23,24,25]. Moreover, transition metal-catalyzed 1,4-addition of enynes serves as a powerful strategy for the concise and efficient synthesis of multi-substituted chiral allenes [26,27,28,29,30]. It is worth noting that although great progress has been achieved for the enantioselective construction of allenyl axial chirality [31,32,33,34,35,36,37,38], only two reports documented the asymmetric concurrent creation of 1,3-stereocenters bearing allenyl axial chirality and central chirality, contributed by the Trost group [11] and the Ma/Zhang group [39], respectively. Both studies exploited the strategy of Pd-catalyzed asymmetric allenylic alkylation employing racemic allenyl acetate electrophile through dynamic kinetic transformation. In connection with our interest in the asymmetric functionalization of indanone derivatives [40,41], herein we report Pd-catalyzed asymmetric allenylic alkylation between racemic allenylic carbonates and indanone-derived β-ketoesters to construct 1,3-stereocenters bearing allenyl axial and central chirality (Scheme 1d), providing alkylation products in good yields (up to 99%) with excellent enantioselectivities (up to 98% ee) and good diastereoselectivities (up to 13:1 dr).

## 2. Results and Discussion

### 2.1. Optimization of the Reaction Conditions

In our preliminary investigation, allenylic carbonate **2a** was selected as the model substrate for the asymmetric alkylation with β-ketoester **1a** in the presence of Cs_2_CO_3_ using a palladium catalyst (Table 1, entries 1–26). Initially, several classic biphosphine ligands were screened to explore the effect of the chiral ligands on the Pd-catalyzed allenylic alkylation reaction. With **L1** or **L3** as the ligand, product **3a** could be afforded in high yields (84% and 89%) with good enantioselectivities (−71% ee and −67% ee) (entries 1 and 3). In the reaction with Trost ligand **L2**, no product was observed (entry 2), while **L4** afforded product **3a** in 55% yield with −73% ee and 15:1 dr (entry 4). Further screening led to the observation that ligands **L5**–**L7** were sluggish for this allenylation, whereas ligand **L6** afforded the product **3a** with high diastereoselectivity (11:1) but low yield and enantioselectivity (20%, −7% ee) (entry 6). Then, the ligand (*R*)- and (*S*)-SegPhos were applied in this reaction. Interestingly, using ligand (*S*)-SegPhos (**L9**) the product **3a** was obtained in 90% yield with 4:1 dr and 91% ee (entry 9). Subsequently, increasing the amount of **2a** further increased the yield of **3a** to 97%, and the enantioselectivity of **3a** to 93%, respectively (entry 10). With **L9** as the ligand, subsequent investigation on the solvent effect showed that THF was the best one, affording 97% yield **3a** with 6:1 dr and 92% ee (entry 15). Next, we investigated the effect of base, and observed that the product **3a** was formed in 98% yield, with 7:1 dr and 93% ee (entry 20), when the base of the reaction was NaHCO_3_. Further optimization was performed and found that the reaction at 0.05 M could give product **3a** in 98% yield with 8:1 dr and 96% ee at −10 °C.

### 2.2. Substrate Scope for the Asymmetric Alkylations of β-Ketoester ***1***

**Scheme 2:**
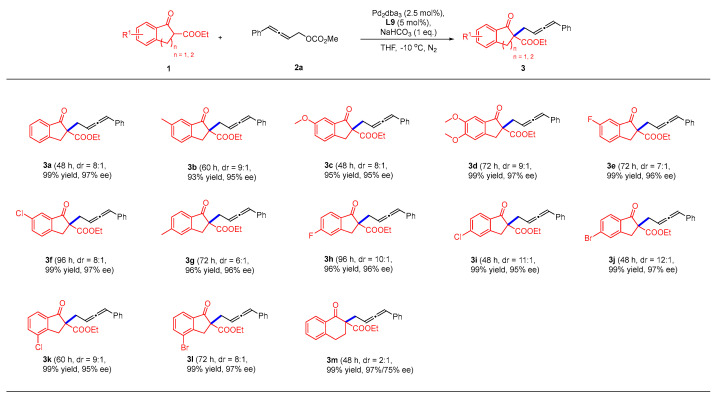
Substrate scope for the reactions of β-ketoesters **1** with arylallenyl carbonate **2a.** The reaction was carried out on a 0.2 mmol scale with Pd_2_dba_3_ (2.5 mol%) and **L9** (5 mol%) in 4.0 mL THF with NaHCO_3_ (0.2 mmol); the ratio of **1**/**2a** is 1.0/1.2; isolated yields are given; the dr was determined by ^1^H NMR of crude product; the ee was determined by chiral HPLC.

Having established the optimized reaction conditions (Table 1, entry 26), the scope of β-ketoester **1** was then examined with allenylic carbonate **2a** (Scheme 2). We evaluated the electronic nature and position of the substituents on the 1H-indanone backbone of the substrates. The products **3b**, **3c,** and **3g** bearing electron-donating substituents were obtained in high yields (93–96% yield) with excellent enantioselectivities (95–96% ee) and good diastereoselectivities (6:1–9:1 dr). Furthermore, the disubstituted substrate **1d** was also successfully transformed into **3d** in 99% yield, with 97% ee and 9:1 dr. Meanwhile, substrates (**1e**, **1f**, and **1h**–**1l**) bearing halogen groups also engaged in the asymmetric alkylation reaction well and afforded products (**3e**, **3f**, and **3h**–**3l**) in 96–99% yields, with high enantioselectivities (95–97% ee) and diastereoselectivities (7:1–12:1 dr). Notably, the six-membered cyclic β-ketoester (**1m**) underwent the alkylation reaction smoothly to afford product **3m** in high yield and excellent enantioselectivity (99%, 97% ee), though with a moderate dr (2:1).

### 2.3. Substrate Scope for the Asymmetric Alkylations of Allenylic Carbonates ***2***

**Scheme 3:**
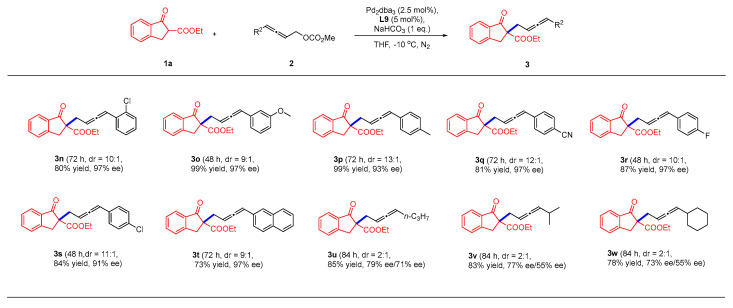
Substrate scope for the reactions of β-ketoester **1a** with allenylic carbonates **2.** The reaction was carried out on a 0.2 mmol scale with Pd_2_dba_3_ (2.5 mol%) and **L9** (5 mol%) in 4.0 mL THF with NaHCO_3_ (0.2 mmol); the ratio of **1a**/**2** is 1.0/1.2; isolated yields are given; the dr was determined by ^1^H NMR of crude product; the ee was determined by chiral HPLC.

Next, the substrate scope with respect to allenylic carbonates **2** were investigated (Scheme 3). The electronic nature and position of the substituents on the arylallenyl carbonates backbone of the substrates were also examined. When a chloro group was present at the C2 position in the aryl, high yield and enantioselectivity were observed for the formation of the alkylation product **3n** (80% yield, 97% ee). The products (**3o** and **3p**) bearing electron-donating substituents were obtained in 99% yield with 93–97% ee. Meanwhile, halogenation in the aryl C4 position of the substrates (**2f** and **2g**) were well-tolerated, giving the corresponding products (**3r** and **3s**) with 91–97% ee and a slight decrease in yield (84–87%). Moreover, 2-naphthyl-substituted allenylic carbonate also worked smoothly (73% yield, 97% ee). Compared to model substrate **2a**, various substituted arylallenyl carbonates (**2b**–**2h**) could afford the target products (**3n**–**3t**) with relatively higher diastereoselectivities as expected. In addition, substrates **2i** with an n-propyl group and **2j** with isopropyl afforded products **3u** and **3v** with slightly decreased enantioselectivities and diastereoselectivities. The asymmetric alkylation of allenylic carbonate with symmetrical cyclohexyl substituent delivered the product **3w** in moderate enantioselectivity and diastereoselectivity, albeit with a lower yield.

### 2.4. Gram-Scale Reaction and Product Derivatization

**Scheme 4:**
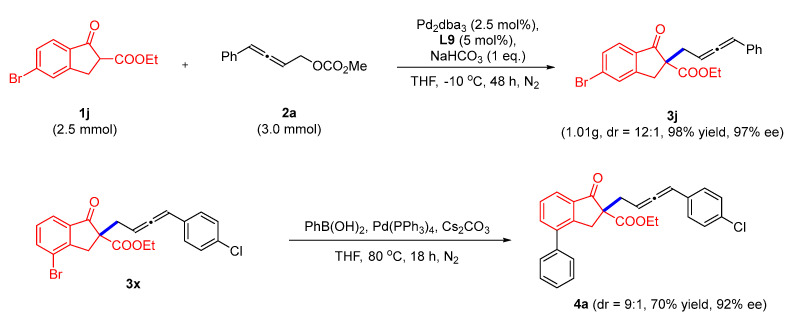
Gram-scale reaction and product derivatization.

To demonstrate the practicality of the transformation, a gram-scale reaction was conducted and found that the reaction of β-ketoester **1j** (2.5 mmol) with allenylic carbonate **2a** (3.0 mmol) gave product **3j** (1.01 g) in 98% yield, with 97% ee and 12:1 dr (Scheme 4). Target product **3x** was found to undergo Suzuki coupling smoothly with phenyl boronic acid, affording compound **4a** (Scheme 4) with a biphenyl motif (70% yield, 92% ee, 9:1 dr) [42].

### 2.5. Plausible Mechanism of the Palladium-Catalyzed Alkylation of β-Ketoester ***1***

**Scheme 5:**
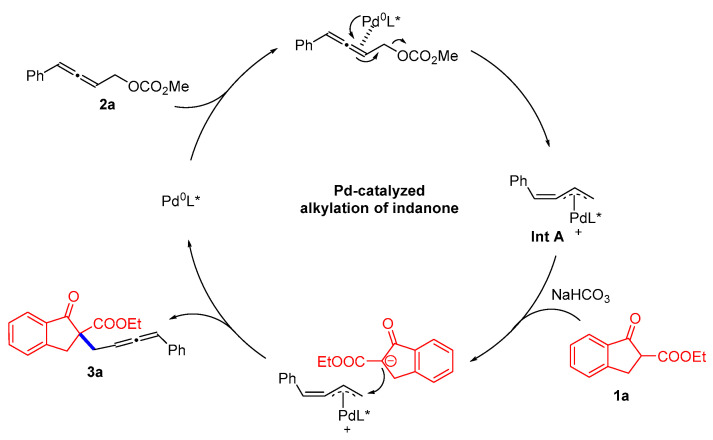
Plausible mechanism of the palladium-catalyzed alkylation of β-ketoester **1**.

A plausible mechanism for the palladium-catalyzed allenylic alkylation of indanone β-ketoester is depicted in Scheme 5 [43,44]. Oxidative addition of **2a** with Pd(0) would immediately undergo delocalization to yield intermediate **A**. In the presence of NaHCO_3_, the nucleophile **1a** attacks the terminal carbon atom of intermediate **A** to afford the target product **3a** and regenerate Pd(0).

## 3. Materials and Methods

### 3.1. General Information

Unless otherwise noted, materials were purchased from commercial suppliers and used without further purification. Column chromatography was performed on silica gel (200~300 mesh). Enantiomeric excesses (ee) were determined by HPLC (Agilent, Palo Alto, CA, USA) using corresponding commercial chiral columns as stated at 30 °C with UV detector at 254 nm. Optical rotations (JiaHang Instruments, Shanghai, China) were reported as follows: ${\left[\alpha \right]}_{D}^{T}$ (*c* g/100 mL, solvent). All ^1^H NMR and ^19^F NMR spectra were recorded on a Bruker Avance II 400 MHz (Bruker, Karlsruhe, Germany) and Bruker Avance III 600 MHz (Bruker, Karlsruhe, Germany), respectively, ^13^C NMR spectra were recorded on a Bruker Avance II 101 MHz or Bruker Avance III 151 MHz with chemical shifts reported as ppm (in CDCl_3_, TMS as an internal standard). Data for ^1^H NMR are recorded as follows: chemical shift (δ, ppm), multiplicity (s = singlet, d = doublet, t = triplet, m = multiplet, br = broad singlet, dd = double doublet, coupling constants in Hz, integration). HRMS (ESI) was obtained with a HRMS/MS instrument (LTQ Orbitrap XL TM, Agilent, Palo Alto, CA, USA). The characterization data is available in Supplementary Material.

### 3.2. Procedure for the Synthesis of Compounds ***3***

(*S*)-SegPhos (**L9**) (5 mol%) and Pd_2_dba_3_ (2.5 mol%) were stirred in THF (4 mL) in a Schlenk tube under a nitrogen atmosphere at room temperature for 10 min. To this Schlenk tube were added **1** (0.20 mmol, 1.0 equiv), NaHCO_3_ (0.20 mmol, 1.0 equiv), and **2** (0.24 mmol, 1.2 equiv), then the reaction mixture was stirred at −10 °C. When compound **1** was consumed as checked by TLC, the reaction was stopped and purified by column chromatography (petroleum ether/ethyl acetate = 30:1) on silica gel directly to give product **3**.

*Ethyl 1-oxo-2-(4-phenylbuta-2,3-dien-1-yl)-2,3-dihydro-1H-indene-2-carboxylate (* **3a** *)*

Prepared according to the procedure within 48 h as light yellow liquid (65.8 mg, 99% yield, dr = 8:1). ${\left[\alpha \right]}_{D}^{17}$ = 84.324 (*c* 0.37, CH_2_Cl_2_); ^1^H NMR (400 MHz, chloroform-*d*) δ 7.77 (d, *J* = 7.7 Hz, 1H), 7.06–7.55 (m, 1H), 7.51–7.33 (m, 3H), 7.31–7.27 (m, 1H), 7.21–7.16 (m, 3H), 6.08 (dt, *J* = 5.9, 2.7 Hz, 1H), 5.48 (q, *J* = 6.9 Hz, 1H), 4.09 (q, *J* = 7.1 Hz, 2H), 3.75 (d, *J* = 17.4 Hz, 1H), 3.24 (d, *J* = 17.4 Hz, 1H), 3.01 (ddd, *J* = 14.7, 7.0, 2.8 Hz, 1H), 2.68 (ddd, *J* = 14.7, 7.2, 2.7 Hz, 1H), 1.17 (t, *J* = 7.1 Hz, 3H); ^13^C NMR (101 MHz, Chloroform-*d*) δ 206.5, 201.8, 170.4, 153.2, 135.3, 135.3, 134.1, 128.5, 127.7, 127.0, 126.8, 126.4, 124.7, 95.5, 90.0, 61.8, 60.3, 36.5, 34.2, 14.0. HRMS (ESI) *m*/*z* Calcd. for C_22_H_20_NaO_3_ ([M + Na]^+^) 335.1305, found 335.1298. Enantiomeric excess was determined to be 97% (determined by HPLC using chiral AD-H column, hexane/2-propanol = 50/1, λ = 254 nm, 30 °C, 0.8 mL/min, t_major_ = 26.2 min, t_minor_ = 24.5 min).

*Ethyl 6-methyl-1-oxo-2-(4-phenylbuta-2,3-dien-1-yl)-2,3-dihydro-1H-indene-2-carboxylate (* **3b** *)*

Prepared according to the procedure within 60 h as light yellow liquid (64.4 mg, 93% yield, dr = 9:1). ${\left[\alpha \right]}_{D}^{17}$ = 77.698 (*c* 0.56, CH_2_Cl_2_); ^1^H NMR (400 MHz, chloroform-*d*) δ 7.58 (s, 1H), 7.45–7.39 (m, 1H), 7.36–7.26 (m, 3H), 7.24–7.18 (m, 3H), 6.10 (dt, *J* = 6.1, 2.7 Hz, 1H), 5.49 (q, *J* = 6.8 Hz, 1H), 4.11 (q, *J* = 7.1 Hz, 2H), 3.71 (d, *J* = 17.2 Hz, 1H), 3.21 (d, *J* = 17.2 Hz, 1H), 3.01 (ddd, *J* = 14.6, 7.1, 2.8 Hz, 1H), 2.70 (ddd, *J* = 14.5, 7.2, 2.6 Hz, 1H), 2.42 (s, 3H), 1.19 (t, *J* = 7.1 Hz, 3H); ^13^C NMR (101 MHz, Chloroform-*d*) δ 206.5, 202.0, 170.6, 150.7, 137.7, 136.7, 135.4, 134.1, 128.5, 127.0, 126.8, 126.1, 124.6, 95.4, 90.1, 61.7, 60.6, 36.2, 34.2, 21.1, 14.0. HRMS (ESI) *m*/*z* Calcd. for C_23_H_22_NaO_3_ ([M + Na]^+^) 369.1461, found 369.1456. Enantiomeric excess was determined to be 95% (determined by HPLC using chiral AD-H column, hexane/2-propanol = 50/1, λ = 254 nm, 30 °C, 0.8 mL/min, t_major_ = 27.9 min, t_minor_ = 22.4 min).

*Ethyl 6-methoxy-1-oxo-2-(4-phenylbuta-2,3-dien-1-yl)-2,3-dihydro-1H-indene-2-carboxylate (* **3c** *)*

Prepared according to the procedure within 48 h as light yellow liquid (68.8 mg, 95% yield, dr = 8:1). ${\left[\alpha \right]}_{D}^{17}$ = 71.161 (*c* 0.27, CH_2_Cl_2_); ^1^H NMR (400 MHz, chloroform-*d*) δ 7.40–7.32 (m, 4H), 7.27 (d, *J* = 1.9 Hz, 2H), 7.25–7.15 (m, 2H), 6.16 (dt, *J* = 5.8, 2.7 Hz, 1H), 5.55 (q, *J* = 6.9 Hz, 1H), 4.18 (q, *J* = 7.1 Hz, 2H), 3.91 (s, 3H), 3.72 (d, *J* = 17.0 Hz, 1H), 3.24 (d, *J* = 17.0 Hz, 1H), 3.06 (ddd, *J* = 14.6, 7.1, 2.8 Hz, 1H), 2.78 (ddd, *J* = 14.6, 7.2, 2.7 Hz, 1H), 1.25 (t, *J* = 7.1 Hz, 3H); ^13^C NMR (101 MHz, Chloroform-*d*) δ 206.5, 201.9, 170.5, 159.7, 146.2, 136.5, 134.1, 128.5, 127.1, 127.0, 126.8, 124.9, 105.7, 95.4, 90.0, 61.7, 61.0, 55.6, 35.8, 34.2, 14.0. HRMS (ESI) *m*/*z* Calcd. for C_23_H_22_NaO_4_ ([M + Na]^+^) 385.1410, found 385.1404. Enantiomeric excess was determined to be 95% (determined by HPLC using chiral OD-H column, hexane/2-propanol = 50/1, λ = 254 nm, 30 °C, 0.8 mL/min, t_major_ = 31.3 min, t_minor_ = 35.8 min).

*Ethyl 5,6-dimethoxy-1-oxo-2-(4-phenylbuta-2,3-dien-1-yl)-2,3-dihydro-1H-indene-2-carboxyl* *ate (* **3d** *)*

Prepared according to the procedure within 72 h as light yellow liquid (77.6 mg, 99% yield, dr = 9:1). ${\left[\alpha \right]}_{D}^{17}$ = 100.27 (*c* 0.74, CH_2_Cl_2_); ^1^H NMR (400 MHz, chloroform-*d*) δ 7.32–7.22 (m, 2H), 7.20–7.10 (m, 4H), 6.79 (s, 1H), 6.05 (dt, *J* = 6.1, 2.8 Hz, 1H), 5.48 (q, *J* = 6.9 Hz, 1H), 4.10 (q, *J* = 7.1 Hz, 2H), 3.92 (s, 3H), 3.89 (s, 3H), 3.63 (d, *J* = 17.1 Hz, 1H), 3.20–3.09 (m, 1H), 2.97 (ddd, *J* = 14.8, 7.1, 2.7 Hz, 1H), 2.71 (ddd, *J* = 14.7, 7.0, 2.8 Hz, 1H), 1.18 (t, *J* = 7.1 Hz, 3H); ^13^C NMR (101 MHz, Chloroform-*d*) δ 206.3, 200.3, 170.7, 156.0, 149.7, 148.8, 134.1, 128.5, 128.0, 126.9, 126.8, 107.3, 104.9, 95.4, 90.1, 61.7, 60.6, 56.2, 56.1, 36.1, 34.0, 14.0. HRMS (ESI) *m*/*z* Calcd. for C_24_H_24_NaO_5_ ([M + Na]^+^) 415.1516, found 415.1507. Enantiomeric excess was determined to be 97% (determined by HPLC using chiral OD-H column, hexane/2-propanol = 7/3, λ = 254 nm, 30 °C, 0.8 mL/min, t_major_ = 25.3 min, t_minor_ = 33.9 min).

*Ethyl 6-fluoro-1-oxo-2-(4-phenylbuta-2,3-dien-1-yl)-2,3-dihydro-1H-indene-2-carboxylate* *(* **3e** *)*

Prepared according to the procedure within 72 h as light yellow liquid (69.3 mg, 99% yield, dr = 7:1). ${\left[\alpha \right]}_{D}^{18}$ = 76.074 (*c* 0.65, CH_2_Cl_2_); ^1^H NMR (600 MHz, chloroform-*d*) δ 7.37 (dd, *J* = 7.4, 2.5 Hz, 1H), 7.33 (dd, *J* = 8.5, 4.3 Hz, 1H), 7.29–7.22 (m, 3H), 7.20–7.13 (m, 3H), 6.05 (dt, *J* = 6.0, 2.7 Hz, 1H), 5.47 (q, *J* = 6.7 Hz, 1H), 4.08 (qd, *J* = 7.1, 1.2 Hz, 2H), 3.67 (d, *J* = 17.1 Hz, 1H), 3.19 (d, *J* = 17.1 Hz, 1H), 2.97 (ddd, *J* = 14.9, 7.0, 2.8 Hz, 1H), 2.73 (ddd, *J* = 14.9, 7.0, 2.8 Hz, 1H), 1.16 (t, *J* = 7.2 Hz, 3H); ^13^C NMR (151 MHz, Chloroform-*d*) δ 206.3, 201.1, 170.1, 162.4 (d, *J* = 166.7 Hz), 148.6, 137.0, 133.9, 128.6, 127.9 (d, *J* = 5.1 Hz), 127.1, 126.8, 122.9, 110.3 (d, *J* = 15.2 Hz), 95.8, 89.9, 61.9, 61.2, 35.9, 34.0, 14.0; ^19^F NMR (376 MHz, Chloroform-*d*) δ −104.2–−123.6 (m). HRMS (ESI) *m*/*z* Calcd. for C_22_H_19_FNaO_3_ ([M + Na]^+^) 373.1210, found 373.1202. Enantiomeric excess was determined to be 96% (determined by HPLC using chiral IC-IB-H column, hexane/2-propanol = 9/1, λ = 254 nm, 30 °C, 0.6 mL/min, t_major_ = 33.4 min, t_minor_ = 32.0 min).

*Ethyl 6-chloro-1-oxo-2-(4-phenylbuta-2,3-dien-1-yl)-2,3-dihydro-1H-indene-2-carboxylate* *(* **3f** *)*

Prepared according to the procedure within 96 h as light yellow liquid (72.5 mg, 99% yield, dr = 8:1). ${\left[\alpha \right]}_{D}^{16}$ = 104.13 (*c* 0.70, CH_2_Cl_2_); ^1^H NMR (600 MHz, chloroform-*d*) δ 7.69 (d, *J* = 2.1 Hz, 1H), 7.47 (dd, *J* = 8.1, 2.1 Hz, 1H), 7.29 (d, *J* = 8.2 Hz, 1H), 7.28–7.24 (m, 2H), 7.20–7.16 (m, 1H), 7.15–7.12 (m, 2H), 6.04 (dt, *J* = 6.0, 2.7 Hz, 1H), 5.46 (q, *J* = 6.7 Hz, 1H), 4.08 (q, *J* = 7.1 Hz, 2H), 3.67 (d, *J* = 17.4 Hz, 1H), 3.18 (d, *J* = 17.4 Hz, 1H), 2.96 (ddd, *J* = 15.0, 7.0, 2.7 Hz, 1H), 2.74 (ddd, *J* = 14.9, 6.9, 2.8 Hz, 1H), 1.15 (t, *J* = 7.1 Hz, 3H); ^13^C NMR (151 MHz, Chloroform-*d*) δ 206.2, 200.7, 170.1, 151.3, 136.9, 135.2, 134.1, 133.8, 128.5, 127.6, 127.1, 126.8, 124.3, 95.9, 89.8, 62.0, 60.8, 36.0, 34.0, 14.0. HRMS (ESI) *m*/*z* Calcd. for C_22_H_19_ClNaO_3_ ([M + Na]^+^) 389.0915, found 389.0911. Enantiomeric excess was determined to be 97% (determined by HPLC using chiral AD-H column, hexane/2-propanol = 50/1, λ = 254 nm, 30 °C, 0.8 mL/min, t_major_ = 37.9 min, t_minor_ = 30.4 min).

*Ethyl 5-methyl-1-oxo-2-(4-phenylbuta-2,3-dien-1-yl)-2,3-dihydro-1H-indene-2-carboxylate (* **3g** *)*

Prepared according to the procedure within 72 h as light yellow liquid (66.5 mg, 96% yield, dr = 6:1); ${\left[\alpha \right]}_{D}^{16}$ = 69.372 (*c* 0.38, CH_2_Cl_2_); ^1^H NMR (400 MHz, chloroform-*d*) δ 7.65 (d, *J* = 7.8 Hz, 1H), 7.30–7.23 (m, 2H), 7.18 (d, *J* = 7.1 Hz, 5H), 6.06 (dt, *J* = 5.9, 2.8 Hz, 1H), 5.47 (q, *J* = 6.9 Hz, 1H), 4.08 (q, *J* = 7.1 Hz, 2H), 3.68 (d, *J* = 17.4 Hz, 1H), 3.17 (d, *J* = 17.3 Hz, 1H), 2.98 (ddd, *J* = 14.7, 7.0, 2.8 Hz, 1H), 2.67 (ddd, *J* = 14.6, 7.2, 2.7 Hz, 1H), 2.40 (s, 3H), 1.16 (t, *J* = 7.1 Hz, 3H); ^13^C NMR (101 MHz, Chloroform-*d*) δ 206.4, 201.3, 170.6, 153.8, 146.8, 143.3, 134.1, 133.0, 129.1, 128.5, 127.0, 126.8, 124.5, 95.4, 90.1, 61.7, 60.4, 36.3, 34.2, 22.1, 14.0. HRMS (ESI) *m*/*z* Calcd. for C_23_H_22_NaO_3_ ([M + Na]^+^) 369.1461, found 369.1455. Enantiomeric excess was determined to be 96% (determined by HPLC using chiral AD-H column, hexane/2-propanol = 50/1, λ = 254 nm, 30 °C, 0.8 mL/min, t_major_ = 33.3 min, t_minor_ = 30.3 min).

*Ethyl 5-fluoro-1-oxo-2-(4-phenylbuta-2,3-dien-1-yl)-2,3-dihydro-1H-indene-2-carboxylate (* **3h** *)*

Prepared according to the procedure within 96 h as light yellow liquid (67.2 mg, 96% yield, dr = 10:1). ${\left[\alpha \right]}_{D}^{17}$ = 84.648 (*c* 0.47, CH_2_Cl_2_); ^1^H NMR (600 MHz, chloroform-*d*) δ 7.75 (dd, *J* = 8.4, 5.2 Hz, 1H), 7.31–7.25 (m, 2H), 7.22–7.15 (m, 3H), 7.08–7.01 (m, 2H), 6.06 (dt, *J* = 6.0, 2.8 Hz, 1H), 5.47 (q, *J* = 6.8 Hz, 1H), 4.09 (q, *J* = 7.1 Hz, 2H), 3.71 (d, *J* = 17.5 Hz, 1H), 3.20 (d, *J* = 17.6 Hz, 1H), 2.98 (ddd, *J* = 14.9, 7.0, 2.8 Hz, 1H), 2.70 (ddd, *J* = 14.9, 6.9, 2.7 Hz, 1H), 1.17 (t, *J* = 7.1 Hz, 3H); ^13^C NMR (151 MHz, Chloroform-*d*) δ 206.3, 199.9, 170.1, 167.5 (d, *J* = 257.6 Hz), 156.2 (d, *J* = 9.9 Hz), 133.9, 131.7, 128.6, 127.1, 127.0 (d, *J* = 10.8 Hz), 126.8, 116.2 (d, *J* = 23.9 Hz), 113.2 (d, *J* = 22.7 Hz), 95.7, 89.9, 61.9, 60.6, 36.2, 34.0, 14.0; ^19^F NMR (377 MHz, Chloroform-*d*) δ -101.5 (t, *J* = 9.4 Hz). HRMS (ESI) *m*/*z* Calcd. for C_22_H_19_FNaO_3_ ([M + Na]^+^) 373.1210, found 373.1202. Enantiomeric excess was determined to be 96% (determined by HPLC using chiral AD-H column, hexane/2-propanol = 50/1, λ = 254 nm, 30 °C, 0.8 mL/min, t_major_ = 26.1 min, t_minor_ = 24.0 min).

*Ethyl 5-chloro-1-oxo-2-(4-phenylbuta-2,3-dien-1-yl)-2,3-dihydro-1H-indene-2-carboxylate (* **3i** *)*

Prepared according to the procedure within 48 h as light yellow liquid (72.5 mg, 99% yield, dr = 11:1). ${\left[\alpha \right]}_{D}^{18}$ = 69.762 (*c* 0.51, CH_2_Cl_2_); ^1^H NMR (400 MHz, chloroform-*d*) δ 7.70 (d, *J* = 8.1 Hz, 1H), 7.51–7.41 (m, 1H), 7.35 (dd, *J* = 10.2, 2.0 Hz, 2H), 7.28 (d, *J* = 1.8 Hz, 1H), 7.22 (d, *J* = 7.2 Hz, 1H), 7.20–7.15 (m, 2H), 6.07 (dt, *J* = 6.0, 2.9 Hz, 1H), 5.49 (q, *J* = 6.6 Hz, 1H), 4.11 (q, *J* = 7.1 Hz, 2H), 3.71 (d, *J* = 17.5 Hz, 1H), 3.22 (d, *J* = 17.5 Hz, 1H), 2.99 (ddd, *J* = 14.9, 7.0, 2.8 Hz, 1H), 2.75 (ddd, *J* = 14.9, 6.8, 2.8 Hz, 1H), 1.19 (t, *J* = 7.1 Hz, 3H); ^13^C NMR (101 MHz, Chloroform-*d*) δ 206.3, 200.4, 170.1, 154.6, 141.9, 133.9, 133.8, 128.6, 128.6, 127.2, 126.7, 126.7, 125.7, 95.9, 89.8, 61.9, 60.5, 36.1, 33.9, 14.0. HRMS (ESI) *m*/*z* Calcd. for C_22_H_19_ClNaO_3_ ([M + Na]^+^) 389.0915, found 389.0908. Enantiomeric excess was determined to be 95% (determined by HPLC using chiral AD-H column, hexane/2-propanol = 50/1, λ = 254 nm, 30 °C, 0.8 mL/min, t_major_ = 25.3 min, t_minor_ = 22.8 min).

*Ethyl 5-bromo-1-oxo-2-(4-phenylbuta-2,3-dien-1-yl)-2,3-dihydro-1H-indene-2-carboxylate (* **3j** *)*

Prepared according to the procedure within 48 h as light yellow liquid (81.2 mg, 99% yield, dr = 12:1). ${\left[\alpha \right]}_{D}^{16}$ = 48.041 (*c* 0.69, CH_2_Cl_2_); ^1^H NMR (400 MHz, chloroform-*d*) δ 7.59 (d, *J* = 8.2 Hz, 1H), 7.54–7.46 (m, 2H), 7.30–7.24 (m, 2H), 7.22–7.12 (m, 3H), 6.03 (dt, *J* = 6.1, 2.8 Hz, 1H), 5.46 (q, *J* = 6.7 Hz, 1H), 4.08 (q, *J* = 7.1 Hz, 2H), 3.68 (d, *J* = 17.5 Hz, 1H), 3.19 (d, *J* = 17.5 Hz, 1H), 2.96 (ddd, *J* = 14.9, 6.9, 2.8 Hz, 1H), 2.73 (ddd, *J* = 14.9, 6.8, 2.9 Hz, 1H), 1.16 (t, *J* = 7.1 Hz, 3H); ^13^C NMR (101 MHz, Chloroform-*d*) δ 206.2, 200.7, 170.0, 154.7, 134.3, 133.8, 131.4, 130.8, 129.8, 128.6, 127.2, 126.7, 125.7, 95.9, 89.8, 62.0, 60.4, 36.0, 33.9, 14.0. HRMS (ESI) *m*/*z* Calcd. for C_22_H_19_BrNaO_3_ ([M + Na]^+^) 433.0410, found 433.0403. Enantiomeric excess was determined to be 97% (determined by HPLC using chiral AD-H column, hexane/2-propanol = 50/1, λ = 254 nm, 30 °C, 0.8 mL/min, t_major_ = 27.2min, t_minor_ = 24.8 min).

*Ethyl 4-chloro-1-oxo-2-(4-phenylbuta-2,3-dien-1-yl)-2,3-dihydro-1H-indene-2-carboxylate* (**3k**)

Prepared according to the procedure within 60 h as light yellow liquid (72.5 mg, 99% yield, dr = 9:1). ${\left[\alpha \right]}_{D}^{15}$ = 227.30 (*c* 0.67, CH_2_Cl_2_); ^1^H NMR (600 MHz, chloroform-*d*) δ 7.65 (d, *J* = 7.6 Hz, 1H), 7.52 (d, *J* = 7.7 Hz, 1H), 7.33–7.30 (m, 1H), 7.29–7.23 (m, 2H), 7.20–7.13 (m, 3H), 6.06 (dt, *J* = 6.0, 2.7 Hz, 1H), 5.48 (q, *J* = 6.8 Hz, 1H), 4.10 (qd, *J* = 7.2, 2.4 Hz, 2H), 3.69 (d, *J* = 17.8 Hz, 1H), 3.23 (d, *J* = 17.8 Hz, 1H), 3.00 (ddd, *J* = 14.8, 6.7, 2.8 Hz, 1H), 2.70 (ddd, *J* = 14.9, 7.2, 2.7 Hz, 1H), 1.17 (t, *J* = 7.1 Hz, 3H); ^13^C NMR (101 MHz, Chloroform-*d*) δ 206.4, 201.1, 170.0, 150.7, 137.3, 134.8, 133.8, 132.8, 129.3, 128.5, 127.1, 126.8, 122.8, 95.9, 89.8, 62.0, 60.2, 35.6, 34.1, 14.0. HRMS (ESI) *m*/*z* Calcd. for C_22_H_19_ClNaO_3_ ([M + Na]^+^) 389.0915, found 389.0908. Enantiomeric excess was determined to be 95% (determined by HPLC using chiral AD-OD-H column, hexane/2-propanol = 50/1, λ = 254 nm, 30 °C, 0.6 mL/min, t_major_ = 47.7 min, t_minor_ = 51.0 min).

*Ethyl 4-bromo-1-oxo-2-(4-phenylbuta-2,3-dien-1-yl)-2,3-dihydro-1H-indene-2-carboxylate* (**3l**)

Prepared according to the procedure within 72 h as light yellow liquid (81.2 mg, 99% yield, dr = 8:1). ${\left[\alpha \right]}_{D}^{16}$ = 76.255 (*c* 0.74, CH_2_Cl_2_); ^1^H NMR (400 MHz, chloroform-*d*) δ 7.69 (d, *J* = 7.7 Hz, 2H), 7.29–7.22 (m, 3H), 7.21–7.14 (m, 3H), 6.06 (dt, *J* = 6.1, 2.8 Hz, 1H), 5.48 (q, *J* = 6.8 Hz, 1H), 4.11 (qd, *J* = 7.1, 1.5 Hz, 2H), 3.64 (d, *J* = 17.9 Hz, 1H), 3.18 (d, *J* = 17.9 Hz, 1H), 3.00 (ddd, *J* = 14.8, 6.7, 2.9 Hz, 1H), 2.69 (ddd, *J* = 14.8, 7.3, 2.7 Hz, 1H), 1.18 (t, *J* = 7.2 Hz, 3H); ^13^C NMR (101 MHz, Chloroform-*d*) δ 206.4, 201.2, 170.0, 152.8, 138.0, 137.3, 133.8, 129.5, 128.6, 127.1, 126.8, 123.5, 122.0, 95.9, 89.8, 62.0, 60.3, 37.6, 34.2, 14.0. HRMS (ESI) *m*/*z* Calcd. for C_22_H_19_BrNaO_3_ ([M + Na]^+^) 433.0410, found 433.0404. Enantiomeric excess was determined to be 97% (determined by HPLC using chiral AD-OD-H column, hexane/2-propanol = 50/1, λ = 254 nm, 30 °C, 0.6 mL/min, t_major_ = 48.8 min, t_minor_ = 52.0 min).

*Ethyl 1-oxo-2-(4-phenylbuta-2,3-dien-1-yl)-1,2,3,4-tetrahydronaphthalene-2-carboxylate (* **3m** *)*

Prepared according to the procedure within 48 h as light yellow liquid (68.5 mg, 99% yield, dr = 2:1). ${\left[\alpha \right]}_{D}^{16}$ = 9.667 (*c* 0.30, CH_2_Cl_2_); ^1^H NMR (400 MHz, chloroform-*d*) δ 8.05 (dd, *J* = 7.9, 1.5 Hz, 1H), 7.51–7.38 (m, 1H), 7.35–7.21 (m, 5H), 7.21–7.17 (m, 2H), 6.12 (dt, *J* = 6.5, 2.3 Hz, 1H), 5.60 (q, *J* = 7.4 Hz, 1H), 4.16 (qt, *J* = 7.1, 1.4 Hz, 2H), 3.11–2.86 (m, 2H), 2.79 (ddd, *J* = 8.1, 5.5, 2.4 Hz, 2H), 2.62 (dt, *J* = 13.9, 5.5 Hz, 1H), 2.34 (ddd, *J* = 14.1, 9.5, 5.0 Hz, 1H), 1.17 (t, *J* = 7.1Hz, 3H); ^13^C NMR (101 MHz, Chloroform-*d*) δ 207.0, 195.0, 171.5, 143.2, 134.3, 133.6, 131.9, 128.8, 128.6, 128.4, 128.1, 127.0, 126.8, 94.7, 90.2, 61.5, 57.5, 33.8, 30.4, 25.7, 14.1. HRMS (ESI) *m*/*z* Calcd. for C_23_H_22_NaO_3_ ([M + Na]^+^) 369.1461, found 369.1454. Enantiomeric excess was determined to be 97%/75% (determined by HPLC using chiral OJ-H column, hexane/2-propanol = 50/1, λ = 254 nm, 30 °C, 0.8 mL/min, t_major_ = 76.9/68.2 min, t_minor_ = 57.7/82.0 min).

*Ethyl 2-(4-(2-chlorophenyl) buta-2,3-dien-1-yl)-1-oxo-2,3-dihydro-1H-indene-2-carboxylate (**3n**)*

Prepared according to the procedure within 72 h as light yellow liquid (58.6 mg, 80% yield, dr = 10:1). ${\left[\alpha \right]}_{D}^{15}$ = 300.81 (*c* 0.25, CH_2_Cl_2_); ^1^H NMR (400 MHz, chloroform-*d*) δ 7.78–7.76 (m, 1H), 7.61–7.57 (m, 1H), 7.46–7.33 (m, 3H), 7.29 (dd, *J* = 8.0, 1.4 Hz, 1H), 7.21–7.16 (m, 1H), 7.13–7.08 (m, 1H), 6.54 (dt, *J* = 6.5, 2.7 Hz, 1H), 5.50 (q, *J* = 7.0 Hz, 1H), 4.09 (qd, *J* = 7.1, 1.4 Hz, 2H), 3.73 (d, *J* = 17.4 Hz, 1H), 3.22 (d, *J* = 17.3 Hz, 1H), 2.99 (ddd, *J* = 14.7, 7.2, 2.8 Hz, 1H), 2.73 (ddd, *J* = 14.6, 7.1, 2.8 Hz, 1H), 1.16 (t, *J* = 7.1 Hz, 3H); ^13^C NMR (101 MHz, Chloroform-*d*) δ 207.3, 201.8, 170.4, 153.2, 135.4, 135.3, 132.1, 131.8, 129.7, 128.3, 128.0, 127.8, 126.7, 126.4, 124.8, 91.8, 90.2, 61.8, 60.2, 36.5, 33.9, 14.0. HRMS (ESI) *m*/*z* Calcd. for C_22_H_19_ClNaO_3_ ([M + Na]^+^) 389.0915, found 389.0912. Enantiomeric excess was determined to be 97% (determined by HPLC using chiral OJ-H column, hexane/2-propanol = 50/1, λ = 254 nm, 30 °C, 0.8 mL/min, t_major_ = 48.5 min, t_minor_ = 38.6 min).

*Ethyl 2-(4-(3-methoxyphenyl) buta-2,3-dien-1-yl)-1-oxo-2,3-dihydro-1H-indene-2-carboxylate (* **3o** *)*

Prepared according to the procedure within 48 h as light yellow liquid (71.6 mg, 99% yield, dr = 9:1). ${\left[\alpha \right]}_{D}^{21}$ = 64.833 (*c* 0.51, CH_2_Cl_2_); ^1^H NMR (400 MHz, chloroform-*d*) δ 7.76 (d, *J* = 7.7 Hz, 1H), 7.59–7.55 (m, 1H), 7.45–7.33 (m, 2H), 7.20–7.16 (m, 1H), 6.86–6.68 (m, 3H), 6.05 (dt, *J* = 6.1, 2.8 Hz, 1H), 5.48 (q, *J* = 6.8 Hz, 1H), 4.09 (qd, *J* = 7.2, 1.1 Hz, 2H), 3.81 (s, 3H), 3.74 (d, *J* = 17.3 Hz, 1H), 3.24 (d, *J* = 17.4 Hz, 1H), 3.00 (ddd, *J* = 14.7, 6.9, 2.9 Hz, 1H), 2.67 (ddd, *J* = 14.7, 7.2, 2.6 Hz, 1H), 1.16 (t, *J* = 7.1 Hz, 3H); ^13^C NMR (101 MHz, Chloroform-*d*) δ 206.5, 201.9, 170.4, 159.9, 153.2, 135.5, 135.4, 135.2, 129.5, 127.8, 126.5, 124.8, 119.5, 113.0, 111.8, 95.5, 90.2, 61.8, 60.3, 55.2, 36.5, 34.2, 14.0. HRMS (ESI) *m*/*z* Calcd. for C_23_H_22_NaO_4_ ([M + Na]^+^) 385.1410, found 385.1407. Enantiomeric excess was determined to be 97% (determined by HPLC using chiral IB-H column, hexane/2-propanol = 50/1, λ = 254 nm, 30 °C, 0.8 mL/min, t_major_ = 30.8 min, t_minor_ = 26.8 min).

*Ethyl 1-oxo-2-(4-(p-tolyl) buta-2,3-dien-1-yl)-2,3-dihydro-1H-indene-2-carboxylate (* **3p** *)*

Prepared according to the procedure within 72 h as light yellow liquid (68.5 mg, 99% yield, dr = 13:1). ${\left[\alpha \right]}_{D}^{13}$ = 64.270 (*c* 0.45, CH_2_Cl_2_); ^1^H NMR (400 MHz, chloroform-*d*) δ 7.77 (d, *J* = 7.6 Hz, 1H), 7.65–7.52 (m, 1H), 7.48–7.33 (m, 3H), 7.09 (s, 3H), 6.05 (dt, *J* = 6.4, 2.7 Hz, 1H), 5.45 (q, *J* = 6.8 Hz, 1H), 4.10 (q, *J* = 7.1 Hz, 2H), 3.74 (d, *J* = 17.4 Hz, 1H), 3.24 (d, *J* = 17.4 Hz, 1H), 3.00 (ddd, *J* = 14.6, 7.0, 2.8 Hz, 1H), 2.66 (ddd, *J* = 14.5, 7.2, 2.6 Hz, 1H), 2.32 (s, 3H), 1.17 (t, *J* = 7.1 Hz, 3H); ^13^C NMR (101 MHz, Chloroform-*d*) δ 206.3, 201.9, 170.4, 153.3, 136.8, 135.4, 135.2, 131.0, 129.3, 127.7, 126.7, 126.5, 124.8, 95.3, 89.9, 61.8, 60.4, 36.5, 34.3, 21.2, 14.0. HRMS (ESI) *m*/*z* Calcd. for C_23_H_22_NaO_3_ ([M + Na]^+^) 369.1461, found 369.1455. Enantiomeric excess was determined to be 93% (determined by HPLC using chiral AD-OD-H column, hexane/2-propanol = 50/1, λ = 254 nm, 30 °C, 0.6 mL/min, t_major_ = 59.1 min, t_minor_ = 54.5 min).

*Ethyl 2-(4-(4-cyanophenyl) buta-2,3-dien-1-yl)-1-oxo-2,3-dihydro-1H-indene-2-carboxylate (* **3q** *)*

Prepared according to the procedure within 72 h as light yellow liquid (57.9 mg, 81% yield, dr = 12:1). ${\left[\alpha \right]}_{D}^{15}$ = 88.378 (*c* 0.37, CH_2_Cl_2_); ^1^H NMR (400 MHz, chloroform-*d*) δ 7.69 (d, *J* = 7.7 Hz, 1H), 7.51 (dd, *J* = 7.5, 1.2 Hz, 1H), 7.46 (d, *J* = 8.1 Hz, 2H), 7.39–7.28 (m, 2H), 7.23–7.14 (m, 2H), 6.01 (dt, *J* = 6.0, 2.8 Hz, 1H), 5.50 (q, *J* = 6.9 Hz, 1H), 4.01 (q, *J* = 7.1 Hz, 2H), 3.65 (d, *J* = 17.3 Hz, 1H), 3.11 (d, *J* = 17.3 Hz, 1H), 2.90 (ddd, *J* = 14.8, 7.0, 2.9 Hz, 1H), 2.69 (ddd, *J* = 14.8, 7.3, 2.7 Hz, 1H), 1.08 (t, *J* = 7.1 Hz, 3H); ^13^C NMR (101 MHz, Chloroform-*d*) δ 207.6, 201.6, 170.3, 153.0, 139.3, 135.5, 135.3, 132.3, 127.9, 127.2, 126.4, 124.8, 119.0, 110.2, 94.8, 91.0, 61.9, 60.0, 36.5, 33.6, 14.0. HRMS (ESI) *m*/*z* Calcd. for C_23_H_19_NNaO_3_ ([M + Na]^+^) 380.1257, found 380.1248. Enantiomeric excess was determined to be 97% (determined by HPLC using chiral OD-H column, hexane/2-propanol = 95/5, λ = 254 nm, 30 °C, 0.8 mL/min, t_major_ = 41.0 min, t_minor_ = 36.8 min).

*Ethyl 2-(4-(4-fluorophenyl) buta-2,3-dien-1-yl)-1-oxo-2,3-dihydro-1H-indene-2-carboxylate (* **3r** *)*

Prepared according to the procedure within 48 h as light yellow liquid (60.9 mg, 87% yield, dr = 10:1). ${\left[\alpha \right]}_{D}^{17}$ = 79.750 (*c* 0.40, CH_2_Cl_2_); ^1^H NMR (400 MHz, chloroform-*d*) δ 7.76 (d, *J* = 7.7 Hz, 1H), 7.60–7.56 (m, 1H), 7.44–7.35 (m, 2H), 7.17–7.10 (m, 2H), 7.01–6.93 (m, 2H), 6.04 (dt, *J* = 6.3, 2.8 Hz, 1H), 5.47 (q, *J* = 6.8 Hz, 1H), 4.09 (q, *J* = 7.1 Hz, 2H), 3.73 (d, *J* = 17.4 Hz, 1H), 3.21 (d, *J* = 17.3 Hz, 1H), 2.98 (ddd, *J* = 14.8, 6.9, 2.8 Hz, 1H), 2.70 (ddd, *J* = 14.7, 7.1, 2.7 Hz, 1H), 1.16 (t, *J* = 7.1 Hz, 3H); ^13^C NMR (101 MHz, Chloroform-*d*) δ 206.2, 201.8, 170.4, 162.0 (d, *J* = 247.5 Hz), 153.2, 135.3 (d, *J* = 6.1 Hz), 130.0, 128.3, 128.2, 127.8, 126.4, 124.7, 115.5 (d, *J* = 21.2 Hz), 94.6, 90.3, 61.8, 60.2, 36.5, 34.1, 14.0. ^19^F NMR (376 MHz, Chloroform-*d*) δ -102.3 (q, *J* = 6.5 Hz); HRMS (ESI) *m*/*z* Calcd. for C_22_H_19_FNaO_3_ ([M + Na]^+^) 373.1210, found 373.1203. Enantiomeric excess was determined to be 97% (determined by HPLC using chiral AD-OD-H column, hexane/2-propanol = 50/1, λ = 254 nm, 30 °C, 0.6 mL/min, t_major_ = 64.5 min, t_minor_ = 61.0 min).

*Ethyl 2-(4-(4-chlorophenyl) buta-2,3-dien-1-yl)-1-oxo-2,3-dihydro-1H-indene-2-carboxylate (* **3s** *)*

Prepared according to the procedure within 48 h as light yellow liquid (61.5 mg, 84% yield, dr = 11:1). ${\left[\alpha \right]}_{D}^{17}$ = 72.922 (*c* 0.42, CH_2_Cl_2_); ^1^H NMR (400 MHz, chloroform-*d*) δ 7.76 (d, *J* = 7.7 Hz, 1H), 7.60–7.56 (m, 1H), 7.46–7.34 (m, 2H), 7.30–7.19 (m, 2H), 7.15–7.06 (m, 2H), 6.03 (dt, *J* = 6.2, 2.8 Hz, 1H), 5.49 (q, *J* = 6.9 Hz, 1H), 4.09 (q, *J* = 7.1 Hz, 2H), 3.73 (d, *J* = 17.4 Hz, 1H), 3.20 (d, *J* = 17.3 Hz, 1H), 2.98 (ddd, *J* = 14.7, 6.9, 2.9 Hz, 1H), 2.70 (ddd, *J* = 14.7, 7.2, 2.7 Hz, 1H), 1.16 (t, *J* = 7.1 Hz, 3H); ^13^C NMR (101 MHz, Chloroform-*d*) δ 206.5, 201.8, 170.4, 153.1, 135.4, 135.3, 132.6, 132.6, 128.7, 128.0, 127.8, 126.4, 124.7, 94.7, 90.5, 61.8, 60.2, 36.5, 34.0, 14.0. HRMS (ESI) *m*/*z* Calcd. for C_22_H_19_ClNaO_3_ ([M + Na]^+^) 389.0915, found 389.0907. Enantiomeric excess was determined to be 91% (determined by HPLC using chiral IC-IB-H column, hexane/2-propanol = 9/1, λ = 254 nm, 30 °C, 0.6 mL/min, t_major_ = 41.9 min, t_minor_ = 37.0 min).

*Ethyl 2-(4-(naphthalen-2-yl) buta-2,3-dien-1-yl)-1-oxo-2,3-dihydro-1H-indene-2-carboxylate (* **3t** *)*

Prepared according to the procedure within 72 h as light yellow liquid (55.8 mg, 73% yield, dr = 9:1). ${\left[\alpha \right]}_{D}^{16}$ = 94.309 (*c* 0.25, CH_2_Cl_2_); ^1^H NMR (400 MHz, chloroform-*d*) δ 7.84–7.70 (m, 4H), 7.54 (d, *J* = 7.9 Hz, 2H), 7.44 (d, *J* = 6.0 Hz, 2H), 7.40–7.35 (m, 3H), 6.24 (dd, *J* = 6.7, 3.1 Hz, 1H), 5.55 (d, *J* = 6.9 Hz, 1H), 4.10 (q, *J* = 7.4 Hz, 2H), 3.76 (d, *J* = 17.4 Hz, 1H), 3.27 (d, *J* = 17.3 Hz, 1H), 3.04 (dd, *J* = 15.3, 6.7 Hz, 1H), 2.73 (dd, *J* = 15.3, 7.6 Hz, 1H), 1.16 (t, *J* = 7.1 Hz, 3H); ^13^C NMR (101 MHz, chloroform-*d*) δ 207.0, 201.8, 170.4, 153.2, 135.4, 135.3, 133.7, 132.7, 131.6, 128.5, 128.2, 127.7, 126.4, 126.2, 125.7, 125.7, 125.5, 124.7, 124.7, 95.9, 90.3, 61.8, 60.4, 36.5, 34.2, 14.0. HRMS (ESI) *m*/*z* Calcd. for C_26_H_22_NaO_3_ ([M + Na]^+^) 405.1461, found 405.1455. Enantiomeric excess was determined to be 97% (determined by HPLC using chiral AD-H column, hexane/2-propanol = 50/1, λ = 254 nm, 30 °C, 0.8 mL/min, t_major_ = 49.1 min, t_minor_ = 45.2 min).

*Ethyl 2-(hepta-2,3-dien-1-yl)-1-oxo-2,3-dihydro-1H-indene-2-carboxylate* *(* **3u** *)*

Prepared according to the procedure within 84 h as light yellow liquid (50.74 mg, 85% yield, dr = 2:1). ${\left[\alpha \right]}_{D}^{16}$ = 49.275 (*c* 0.14, CH_2_Cl_2_); ^1^H NMR (400 MHz, chloroform-*d*) δ 7.76 (d, *J* = 7.7 Hz, 1H), 7.65–7.57 (m, 1H), 7.48 (d, *J* = 7.7 Hz, 1H), 7.40–7.37 (m, 1H), 5.14–4.83 (m, 2H), 4.24–4.07 (m, 2H), 3.68 (dd, *J* = 17.3, 2.6 Hz, 1H), 3.23 (d, *J* = 17.3 Hz, 1H), 2.83 (dtd, *J* = 15.1, 7.6, 2.5 Hz, 1H), 2.54 (dddd, *J* = 14.5, 7.4, 5.2, 2.6 Hz, 1H), 1.87 (qd, *J* = 7.1, 3.6 Hz, 2H), 1.36 (qd, *J* = 7.4, 2.1 Hz, 2H), 1.21 (t, *J* = 7.1 Hz, 3H), 0.88 (t, *J* = 7.4, 3H); ^13^C NMR (101 MHz, Chloroform-*d*) δ 205.7, 202.1, 170.6, 153.4, 135.4, 135.3, 127.6, 126.4, 124.7, 91.4, 85.6, 61.6, 60.6, 36.2, 34.6, 30.9, 22.3, 14.1, 13.6. HRMS (ESI) *m*/*z* Calcd. for C_19_H_22_NaO_3_ ([M + Na]^+^) 321.1461, found 321.1453. Enantiomeric excess was determined to be 79%/71% (determined by HPLC using chiral AS-AD-H column, hexane/2-propanol = 50/1, λ = 254 nm, 30 °C, 0.6 mL/min, t_major_ = 30.5/26.7 min, t_minor_ = 25.6/28.2 min).

*Ethyl 2-(5-methylhexa-2,3-dien-1-yl)-1-oxo-2,3-dihydro-1H-indene-2-carboxylate (* **3v** *)*

Prepared according to the procedure within 84 h as light yellow liquid (49.5 mg, 83% yield, dr = 2:1). ${\left[\alpha \right]}_{D}^{16}$ = 67.677 (*c* 0.20, CH_2_Cl_2_); ^1^H NMR (600 MHz, chloroform-*d*) δ 7.76 (dd, *J* = 7.7, 3.9 Hz, 1H), 7.65–7.59 (m, 1H), 7.48 (dd, *J* = 7.8, 2.9 Hz, 1H), 7.44–7.35 (m, 1H), 5.15–4.89 (m, 2H), 4.32–4.08 (m, 2H), 3.68 (d, *J* = 17.2 Hz, 1H), 3.24 (dd, *J* = 17.3, 3.7 Hz, 1H), 2.94–2.77 (m, 1H), 2.55 (ddt, *J* = 14.2, 7.6, 2.0 Hz, 1H), 2.20 (dp, *J* = 9.7, 3.1 Hz, 1H), 1.20 (t, *J* = 7.1 Hz, 3H), 0.94 (ddd, *J* = 6.5, 4.6, 1.6 Hz, 6H); ^13^C NMR (151 MHz, Chloroform-*d*) δ 204.2, 202.2, 170.7, 153.4, 135.3, 129.0, 127.7, 126.4, 124.8, 98.9, 86.8, 61.7, 60.6, 36.2, 34.8, 27.9, 22.4, 14.1. HRMS (ESI) *m*/*z* Calcd. for C_19_H_22_NaO_3_ ([M + Na]^+^) 321.1461, found 321.1453. Enantiomeric excess was determined to be 77%/55% (determined by HPLC using chiral AS-AD-H column, hexane/2-propanol = 50/1, λ = 254 nm, 30 °C, 0.6 mL/min, t_major_ = 27.5/24.7 min, t_minor_ = 23.8/26.2 min).

*Ethyl 2-(4-cyclohexylbuta-2,3-dien-1-yl)-1-oxo-2,3-dihydro-1H-indene-2-carboxylate (* **3w** *)*

Prepared according to the procedure within 84 h as light yellow liquid (52.8 mg, 78% yield, dr = 2:1). ${\left[\alpha \right]}_{D}^{16}$ = 43.017 (*c* 0.18, CH_2_Cl_2_); ^1^H NMR (400 MHz, chloroform-*d*) δ 7.80–7.72 (m, 1H), 7.64–7.6 (m, 1H), 7.48 (d, *J* = 7.7 Hz, 1H), 7.44–7.34 (m, 1H), 4.99 (dtt, *J* = 23.8, 6.8, 3.1 Hz, 2H), 4.15 (qd, *J* = 7.1, 1.5 Hz, 2H), 3.68 (d, *J* = 17.4 Hz, 1H), 3.24 (d, *J* = 17.3 Hz, 1H), 2.83 (dddd, *J* = 14.1, 6.9, 4.1, 2.6 Hz, 1H), 2.56 (dddd, *J* = 14.9, 7.9, 5.5, 2.7 Hz, 1H), 1.86 (dddd, *J* = 11.2, 8.7, 5.9, 3.1 Hz, 1H), 1.66 (tt, *J* = 18.8, 7.4 Hz, 5H), 1.39–1.10 (m, 6H), 1.00 (tdd, *J* = 13.6, 7.7, 3.7 Hz, 2H); ^13^C NMR (101 MHz, Chloroform-*d*) δ 204.5, 202.2, 170.7, 153.4, 135.4, 129.0, 128.4, 127.7, 126.4, 124.8, 97.5, 86.4, 61.7, 60.6, 37.2, 36.2, 34.8, 32.9, 26.1, 14.1. HRMS (ESI) *m*/*z* Calcd. for C_22_H_26_NaO_3_ ([M + Na]^+^) 361.1774, found 361.1765. Enantiomeric excess was determined to be 73%/55% (determined by HPLC using chiral AS-AD-H column, hexane/2-propanol = 50/1, λ = 254 nm, 30 °C, 0.6 mL/min, t_major_ = 29.9/26.9 min, t_minor_ = 26.3/27.9 min).

*Ethyl4-bromo-2-(4-(4-chlorophenyl) buta-2,3-dien-1-yl)-1-oxo-2,3-dihydro-1H-indene-2-carboxylate (* **3x** *)*

Prepared according to the procedure within 48 h as light yellow liquid (87.9 mg, 99% yield, dr = 10:1). ${\left[\alpha \right]}_{D}^{16}$ = 101.589 (*c* 1.20, CH_2_Cl_2_); ^1^H NMR (600 MHz, chloroform-*d*) δ 7.72–7.69 (m, 2H), 7.26–7.21 (m, 3H), 7.07 (d, *J* = 8.3 Hz, 2H), 6.01 (dt, *J* = 6.0, 2.8 Hz, 1H), 5.50 (q, *J* = 6.8 Hz, 1H), 4.11 (qd, *J* = 7.1, 3.2 Hz, 2H), 3.62 (d, *J* = 17.8 Hz, 1H), 3.14 (d, *J* = 17.8 Hz, 1H), 2.98 (ddd, *J* = 14.9, 6.7, 2.9 Hz, 1H), 2.72 (ddd, *J* = 14.9, 7.1, 2.8 Hz, 1H), 1.18 (t, *J* = 7.1 Hz, 3H). ^13^C NMR (101 MHz, Chloroform-*d*) δ 206.4, 201.2, 170.0, 152.7, 138.0, 137.3, 132.7, 132.4, 129.6, 128.7, 127.9, 123.4, 122.0, 95.1, 90.2, 62.0, 60.2, 37.6, 33.9, 14.0. HRMS (ESI) *m*/*z* Calcd. for C_22_H_18_BrClNaO_3_ ([M + Na]^+^) 467.0020, found 467.0022. Enantiomeric excess was determined to be 96% (determined by HPLC using chiral AD-OJ-H column, hexane/2-propanol = 50/1, λ = 254 nm, 30 °C, 0.6 mL/min, t_major_ = 92.3 min, t_minor_ = 83.9 min).

### 3.3. Procedure for the Synthesis of Compounds ***4a***

A Schlenk flask under a nitrogen atmosphere was charged with compound **3x** (222 mg, 0.50 mmol, 1.0 eq), phenylboronic acid (73.2 mg, 0.60 mmol, 1.2 eq), Cs_2_CO_3_ (244 mg, 0.75 mmol, 1.5 eq), and Pd(PPh_3_)_4_ (28.9 mg, 25.0 µmol, 5 mol%). THF (5 mL) was added and the mixture was heated to 80 °C for 18 h, when compound **3x** was consumed as checked by TLC, the mixture was cooled to rt and diluted with Et_2_O (15 mL). The mixture was washed with water (15 mL). The aq. layer was extracted with Et_2_O (2 × 25 mL) and the combined org. layers were dried, filtered, and concentrated. The crude product was purified by column chromatography (petroleum ether/ethyl acetate = 20:1) yielding the title compound **4a** as a slightly yellow oil.

*Ethyl2-(4-(4-chlorophenyl)buta-2,3-dien-1-yl)-1-oxo-4-phenyl-2,3-dihydro-1H-indene-2-carboxylate* (**4a***)*

Prepared according to the procedure within 18 h as slightly yellow oil (154 mg, 70% yield, dr = 9:1). ${\left[\alpha \right]}_{D}^{16}$ = 23.014 (*c* 0.37, CH_2_Cl_2_); ^1^H NMR (400 MHz, chloroform-*d*) δ 7.79 (dd, *J* = 18.7, 7.5 Hz, 1H), 7.63 (dd, *J* = 16.9, 7.2 Hz, 1H), 7.55–7.42 (m, 5H), 7.38 (dd, *J* = 6.6, 3.1 Hz, 1H), 7.32–7.27 (m, 1H), 7.16 (dd, *J* = 8.6, 2.1 Hz, 2H), 7.07–7.03 (m, 1H), 6.10–5.91 (m, 1H), 5.61–5.48 (m, 1H), 4.30–4.12 (m, 2H), 3.81 (dd, *J* = 17.5, 11.5 Hz, 1H), 3.22 (dd, *J* = 17.5, 9.8 Hz, 1H), 3.01 (tdd, *J* = 11.7, 7.0, 3.0 Hz, 1H), 2.70 (dddd, *J* = 30.5, 14.6, 7.2, 2.7 Hz, 1H), 1.23 (t, *J* = 7.1 Hz, 3H). ^13^C NMR (101 MHz, Chloroform-*d*) δ 206.5, 201.8, 170.4, 150.6, 140.2, 138.7, 135.6, 132.6, 128.8, 128.7, 128.4, 128.4, 128.4, 127.9, 127.8, 123.8, 115.3, 94.5, 90.5, 61.9, 60.5, 36.5, 34.2, 14.1. HRMS (ESI) *m*/*z* Calcd. for C_28_H_23_ClNaO_3_ ([M + Na]^+^) 465.1228, found 465.1225. Enantiomeric excess was determined to be 92% (determined by HPLC using chiral IF-OD-H column, hexane/2-propanol = 95/5, λ = 254 nm, 30 °C, 0.6 mL/min, t_major_ = 41.3 min, t_minor_ = 44.3 min).

## 4. Conclusions

In conclusion, we have developed Pd(0)-catalyzed asymmetric allenylic alkylation of indanone-derived β-ketoesters by allenyl carbonates. This reaction provides a contribution toward the construction of 1,3-stereocenters bearing allenyl axial and central chirality with high levels of stereocontrol. This work features a broad substrate scope, mild reaction conditions, high efficiency, excellent enantioselectivities, and good diastereoselectivities.

## Figures and Tables

**Table 1:** Optimization of reaction conditions.

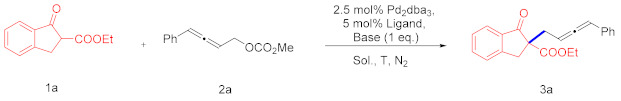								
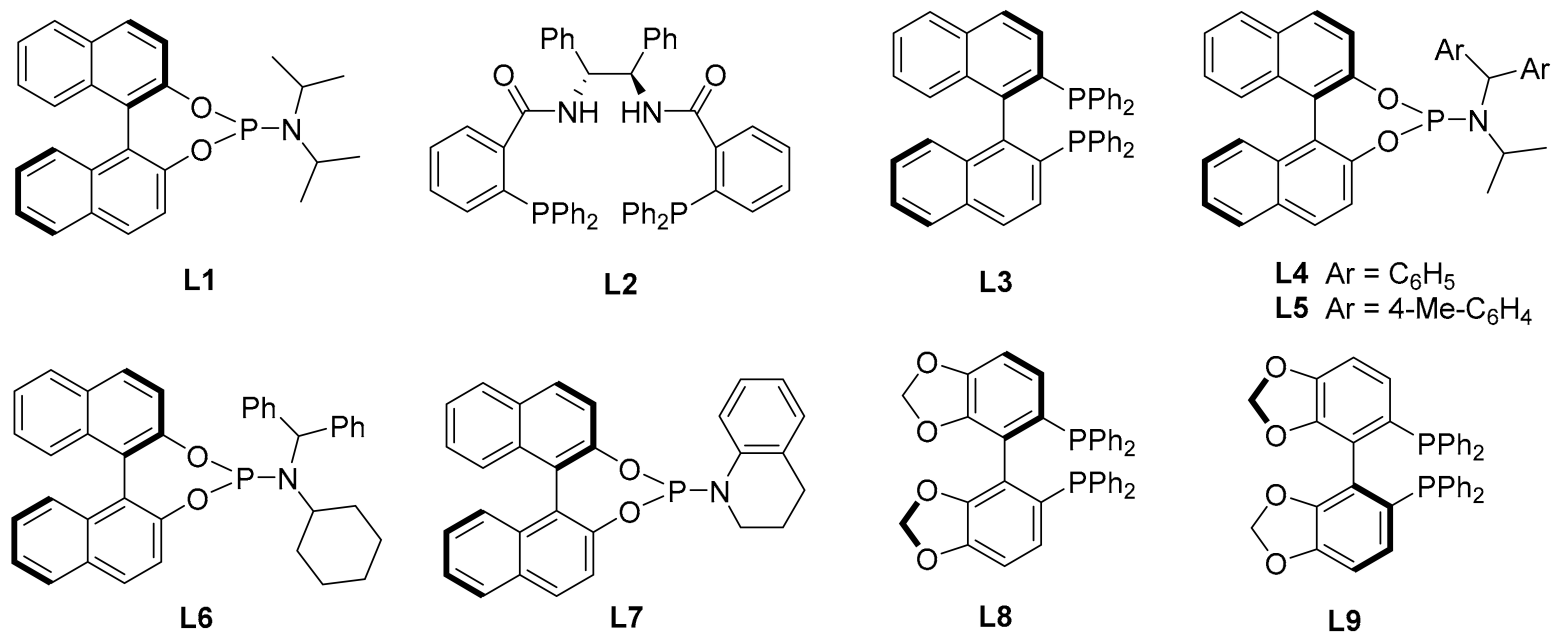								
**Entry *^a^***	**Ligand**	**Base**	**Sol.**	**T [°C]**	**t [h]**	**Yield [%] *^b^***	**dr *^c^***	**ee [%] *^d^***
1	**L1**	Cs_2_CO_3_	DCM	25	0.5	84	10:1	−71/−39
2	**L2**	Cs_2_CO_3_	DCM	25	12	trace	-	-
3	**L3**	Cs_2_CO_3_	DCM	25	3	89	3:1	−67/−69
4	**L4**	Cs_2_CO_3_	DCM	25	12	55	15:1	−73/−17
5	**L5**	Cs_2_CO_3_	DCM	25	48	21	4:1	−23/−33
6	**L6**	Cs_2_CO_3_	DCM	25	24	20	11:1	−7/−5
7	**L7**	Cs_2_CO_3_	DCM	25	7.5	59	5:1	−69/−69
8	**L8**	Cs_2_CO_3_	DCM	25	24	29	5:1	−89/−51
9	**L9**	Cs_2_CO_3_	DCM	25	24	90	4:1	91/81
10 *^e^*	**L9**	Cs_2_CO_3_	DCM	25	6	97	4:1	93/77
11 *^e^*	**L9**	Cs_2_CO_3_	CHCl_3_	25	9	92	5:1	92/61
12 *^e^*	**L9**	Cs_2_CO_3_	DCE	25	12	92	4:1	91/75
13 *^e^*	**L9**	Cs_2_CO_3_	MeCN	25	12	96	5:1	92/81
14 *^e^*	**L9**	Cs_2_CO_3_	Tol.	25	9	98	5:1	92/73
15 *^e^*	**L9**	Cs_2_CO_3_	THF	25	6	97	6:1	92/65
16 *^e^*	**L9**	Cs_2_CO_3_	Dioxane	25	6	98	4:1	93/61
17 *^e^*	**L9**	Et_3_N	THF	25	10	70	6:1	94/71
18 *^e^*	**L9**	C_4_H_9_OK	THF	25	4	90	7:1	92/71
19 *^e^*	**L9**	C_2_H_5_ONa	THF	25	6	98	3:1	90/73
20 *^e^*	**L9**	NaHCO_3_	THF	25	6	98	7:1	93/67
21 *^e^*	**L9**	Na_2_CO_3_	THF	25	10	61	5:1	92/51
22 *^e^*	**L9**	K_2_CO_3_	THF	25	6	98	2:1	93/79
23 *^e^*	**L9**	NaHCO_3_	THF	0	24	98	6:1	95/79
24 *^e^*	**L9**	NaHCO_3_	THF	−10	42	98	7:1	95/70
25 *^e,f^*	**L9**	NaHCO_3_	THF	−10	12	98	7:1	96/71
26 *^e,g^*	**L9**	NaHCO_3_	THF	−10	48	98	8:1	96/77

*^a^* The reaction was conducted with **1a** (0.1 mmol), **2a** (0.11 mmol), base (0.1 mmol), Pd_2_dba_3_ (2.5 mol%) and ligand (5 mol%) in solvent (1.0 mL). *^b^* Isolated yield. *^c^* Detected by ^1^H NMR of the crude product. *^d^* Detected by chiral HPLC analysis. *^e^* **2a** (0.12 mmol). *^f^* THF (0.5 mL) was used. *^g^* THF (2.0 mL) was used.

## Data Availability

The data presented in this study are available in Supplementary Material.

## Supplementary Materials

The following supporting information can be downloaded at: https://www.mdpi.com/article/10.3390/molecules28072927/s1, materials and methods, experimental procedures [33,43,45], characterization data, ^1^H, ^13^C, and ^19^F NMR spectra, HRMS spectrometry data, and HPLC chromatogram.

## Sample Availability

Samples of the compounds are available from the authors.
