# Supplementary material for: Palladium-Catalyzed Stereoselective Construction of 1,3-Stereocenters Displaying Axial and Central Chirality via Asymmetric Alkylations
Source: Molecules. 2023 Mar 24;28(7):2927. doi: 10.3390/molecules28072927 (PMC10096308; doi:10.3390/molecules28072927)
Supplement: Supplementary file 1 [file molecules-28-02927-s001.zip › molecules-2281822-supplementary.pdf]

Palladium-Catalyzed Stereoselective Construction of 1,3-Stereocenters Displaying Axial and Central Chirality via Asymmetric Alkylations

Aiqi Xue, Xingfu Wei, Yue Huang, Jingping Qu and Baomin Wang*

State Key Laboratory of Fine Chemicals, Department of Pharmaceutical Sciences, School of Chemical Engineering, Dalian University of Technology, 2 Linggong Road, Dalian 116024, China

Contents

1. General information--S1
2. Experimental sections of compounds **3** --S1
3. Experimental procedures and characterization of compounds **3** and **4a**-----------------------------S2
4. NMR spectra for compounds --S31

1. General information

Unless otherwise noted, materials were purchased from commercial suppliers and used without further purification. Column chromatography was performed on silica gel (200~300 mesh). Enantiomeric excesses (ee) were determined by HPLC using corresponding commercial chiral columns as stated at 30 °C with UV detector at 254 nm. Optical rotations were reported as follows: $[\alpha]_D^T$ (*c* g/100 mL, solvent). All ^{1}H NMR and ^{19}F NMR spectra were recorded on a Bruker AvanceII 400 MHz and Bruker Avance III 600 MHz respectively, ^{13}C NMR spectra were recorded on a Bruker AvanceII 101 MHz and Bruker Avance III 151 MHz with chemical shifts reported as ppm (in $CDCl_3$, TMS as an internal standard). Data for ^{1}H NMR are recorded as follows: chemical shift (δ, ppm), multiplicity (s = singlet, d = doublet, t = triplet, m = multiplet, br = broad singlet, dd = double doublet, coupling constants in Hz, integration). HRMS (ESI) was obtained with a HRMS/MS instrument (LTQ Orbitrap XL TM).

β-Ketoesters **1** and allenylic carbonates **2** were prepared according to the literature. The racemic products were synthesized using racemic SegPhos and Pd_2dba_3 as the catalyst.

2. Experimental sections of compounds 3

Table S1: Optimization of reaction conditions.

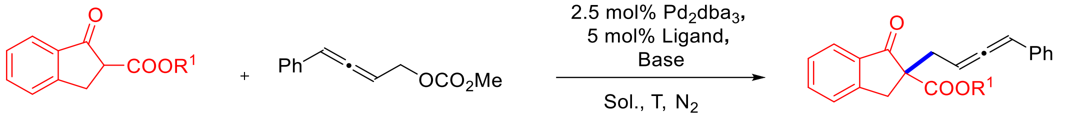

1 **2a** **3**

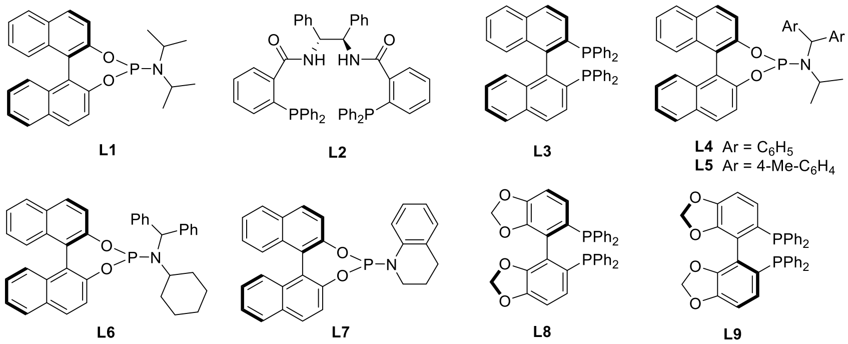

Entry[a]	R[1]	Ligand	Base	Sol.	T [°C]	t [h]	Yield [%][b]	dr[c]	ee [%][d]
1	Et	**L1**	Cs_2CO_3	DCM	25	0.5	84	10:1	-71/-39
2	Et	**L2**	Cs_2CO_3	DCM	25	12	trace	-	-
3	Et	**L3**	Cs_2CO_3	DCM	25	3	89	3:1	-67/-69
4	Et	**L4**	Cs_2CO_3	DCM	25	12	55	15:1	-73/-17
5	Et	**L5**	Cs_2CO_3	DCM	25	48	21	4:1	-23/-33
6	Et	**L6**	Cs_2CO_3	DCM	25	24	20	11:1	-7/-5
7	Et	**L7**	Cs_2CO_3	DCM	25	7.5	59	5:1	-69/-69
8	Et	**L8**	Cs_2CO_3	DCM	25	24	29	5:1	-89/-51
9	Et	**L9**	Cs_2CO_3	DCM	25	24	90	4:1	91/81
10[e]	Et	**L9**	Cs_2CO_3	DCM	25	6	97	4:1	93/77
11[f]	Et	**L9**	Cs_2CO_3	DCM	25	4.5	98	4:1	91/79
12[g]	Et	**L9**	Cs_2CO_3	DCM	25	3	98	4:1	92/77
13[e]	Et	**L9**	Cs_2CO_3	$CHCl_3$	25	9	92	5:1	92/61

14[e]	Et	**L9**	Cs_2CO_3	DCE	25	12	92	4:1	91/75
15[e]	Et	**L9**	Cs_2CO_3	MeCN	25	12	96	5:1	92/81
16[e]	Et	**L9**	Cs_2CO_3	Tol.	25	9	98	5:1	92/73
17[e]	Et	**L9**	Cs_2CO_3	THF	25	6	97	6:1	92/65
18[e]	Et	**L9**	Cs_2CO_3	Dioxane	25	6	98	4:1	93/61
19[e]	Et	**L9**	Et_3N	THF	25	10	70	6:1	94/71
20[e]	Et	**L9**	C_4H_9OK	THF	25	4	90	7:1	92/71
21[e]	Et	**L9**	C_2H_5ONa	THF	25	6	98	3:1	90/73
22[e]	Et	**L9**	$NaHCO_3$	THF	25	6	98	7:1	93/67
23[e]	Et	**L9**	Na_2CO_3	THF	25	10	61	5:1	92/51
24[e]	Et	**L9**	K_2CO_3	THF	25	6	98	2:1	93/79
25[e]	Et	**L9**	$NaHCO_3$	THF	0	24	98	6:1	95/79
26[e]	Et	**L9**	$NaHCO_3$	THF	-10	42	98	7:1	95/70
27[e,h]	Et	**L9**	$NaHCO_3$	THF	-10	12	98	7:1	96/71
28[e,i]	Et	**L9**	$NaHCO_3$	THF	-10	48	98	8:1	96/77
29[e, i, j]	Et	**L9**	$NaHCO_3$	THF	-10	48	69	6:1	96/77
30[e, i, k]	Et	**L9**	$NaHCO_3$	THF	-10	24	95	7:1	95/75
31[e, i, l]	Et	**L9**	$NaHCO_3$	THF	-10	12	74	8:1	95/69
32[e, i]	Me	**L9**	$NaHCO_3$	THF	-10	48	55	6:1	96/70

[a] The reaction was conducted with **1** (0.1 mmol), **2a** (0.11 mmol), base (0.1 mmol), Pd_2dba_3 (2.5 mol%) and ligand (5 mol%) in solvent (1.0 mL). [b] Isolated yield. [c] Detected by ^1H NMR of the crude product. [d] Detected by chiral HPLC analysis. [e] **2a** (0.12 mmol). [f] **2a** (0.15 mmol). [g] **2a** (0.2 mmol). [h] THF (0.5 mL) was used. [i] THF (2.0 mL) was used. [j] Base (0.15 mmol). [k] Base (0.2 mmol). [l] Base (0.25 mmol).

3. Experimental procedures and characterization of compounds 3

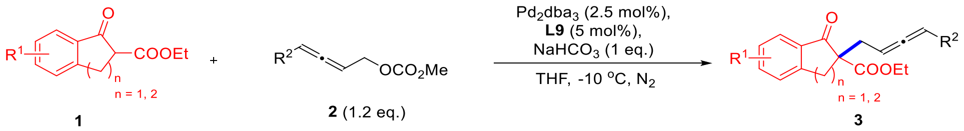

L9 (5 mol%) and Pd_2dba_3 (2.5 mol%) were stirred in THF (4 mL) in a Schlenk flask under a nitrogen atmosphere at room temperature for 10 min. To this Schlenk tube were added **1** (0.20 mmol, 1.0 equiv), $NaHCO_3$ (0.20 mmol, 1.0 equiv) and **2** (0.24 mmol, 1.2 equiv), then the reaction mixture was stirred at -10 °C. When compound **1** was consumed as checked by TLC, the reaction was stopped and purified by column chromatography (petroleum ether/ethyl acetate = 30:1) on silica gel directly to give the product **3**.

Compound 3a

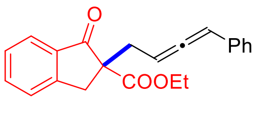

3a

Prepared according to the procedure within 48 h as light yellow liquid (65.8 mg, 99% yield, dr = 8 : 1). $[\alpha]_D^{17}$ = 84.324 (*c* 0.37, CH_2Cl_2); ^1H NMR (400 MHz, Chloroform-*d*) δ 7.77 (d, *J* = 7.7 Hz, 1H), 7.06-7.55 (m, 1H), 7.51 – 7.33 (m, 3H), 7.31 – 7.27 (m, 1H), 7.21 – 7.16 (m, 3H), 6.08 (dt, *J* = 5.9, 2.7 Hz, 1H), 5.48 (q, *J* = 6.9 Hz, 1H), 4.09 (q, *J* = 7.1 Hz, 2H), 3.75 (d, *J* = 17.4 Hz, 1H), 3.24 (d, *J* = 17.4 Hz, 1H), 3.01 (ddd, *J* = 14.7, 7.0, 2.8 Hz, 1H), 2.68 (ddd, *J* = 14.7, 7.2, 2.7 Hz, 1H), 1.17 (t, *J* = 7.1 Hz, 3H); ^{13}C NMR (101 MHz, Chloroform-*d*) δ 206.5, 201.8, 170.4, 153.2, 135.3, 135.3, 134.1, 128.5, 127.7, 127.0, 126.8, 126.4, 124.7, 95.5, 90.0, 61.8, 60.3, 36.5, 34.2, 14.0. HRMS (ESI) m/z Calcd. for $C_{22}H_{20}NaO_3$ ($[M+Na]^+$) 335.1305, Found 335.1298. Enantiomeric excess was

determined to be 97% (determined by HPLC using chiral AD-H column, hexane/2-propanol = 50/1, λ = 254 nm, 30 °C, 0.8 mL/min, t_{major} = 26.2 min, t_{minor} = 24.5 min).

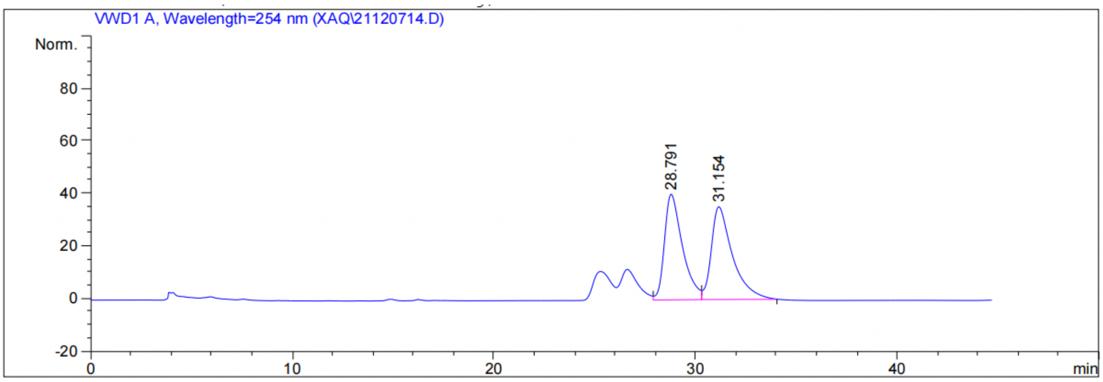

Peak #	RetTime [min]	Type	Width [min]	Area mAU *s	Height [mAU]	Area %
1	28.791	VV	0.9166	2506.95264	40.21124	49.1163
2	31.154	VB	1.0588	2597.16260	35.38195	50.8837

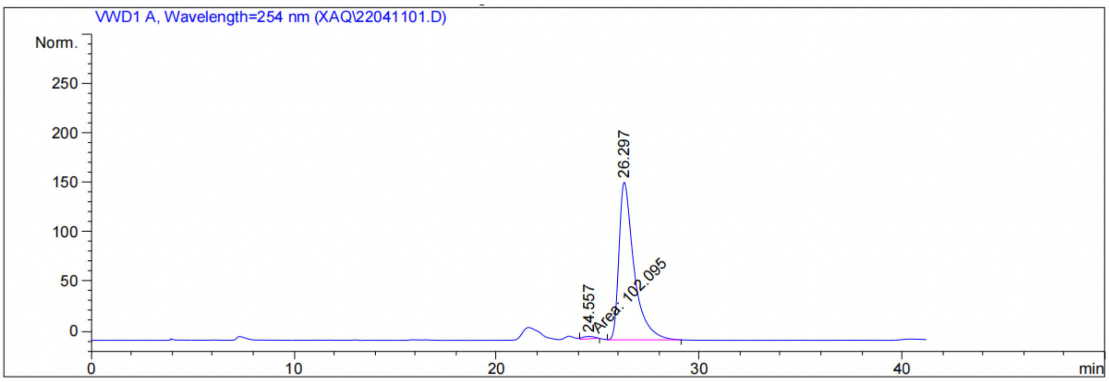

Peak #	RetTime [min]	Type	Width [min]	Area mAU *s	Height [mAU]	Area %
1	24.557	MM	0.6909	102.09455	2.46267	1.2256
2	26.297	VB	0.7617	8228.38867	159.20488	98.7744

Compound 3b

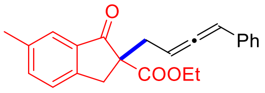

3b

Prepared according to the procedure within 60 h as light yellow liquid (64.4 mg, 93% yield, dr = 9 : 1). $[\alpha]_D^{17}$ = 77.698 (*c* 0.56, CH_2Cl_2); ^1H NMR (400 MHz, Chloroform-*d*) δ 7.58 (s, 1H), 7.45 – 7.39 (m, 1H), 7.36 – 7.26 (m, 3H), 7.24 – 7.18 (m, 3H), 6.10 (dt, *J* = 6.1, 2.7 Hz, 1H), 5.49 (q, *J* = 6.8 Hz, 1H), 4.11 (q, *J* = 7.1 Hz, 2H), 3.71 (d, *J* = 17.2 Hz, 1H), 3.21 (d, *J* = 17.2 Hz, 1H), 3.01 (ddd, *J* = 14.6, 7.1, 2.8 Hz, 1H), 2.70 (ddd, *J* = 14.5, 7.2, 2.6 Hz, 1H), 2.42 (s, 3H), 1.19 (t, *J* = 7.1 Hz, 3H); ^{13}C NMR (101 MHz, Chloroform-*d*) δ 206.5, 202.0, 170.6, 150.7, 137.7, 136.7, 135.4, 134.1, 128.5, 127.0, 126.8, 126.1, 124.6, 95.4, 90.1, 61.7, 60.6, 36.2, 34.2, 21.1, 14.0. HRMS (ESI) m/z Calcd. for $C_{23}H_{22}NaO_3$ ($[M+Na]^+$) 369.1461, Found 369.1456. Enantiomeric excess was determined to be 95% (determined by HPLC using chiral AD-H column, hexane/2-propanol = 50/1, λ = 254 nm, 30 °C, 0.8 mL/min, t_{major} = 27.9min, t_{minor} = 22.4 min).

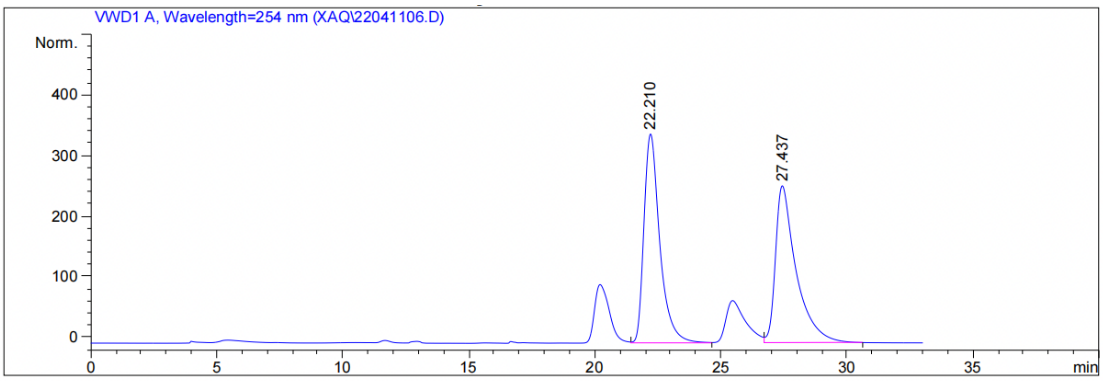

Peak #	RetTime [min]	Type	Width [min]	Area mAU *s	Height [mAU]	Area %
1	22.210	VB	0.6652	1.50798e4	344.35129	50.5578
2	27.437	VB	0.8225	1.47471e4	258.63065	49.4422

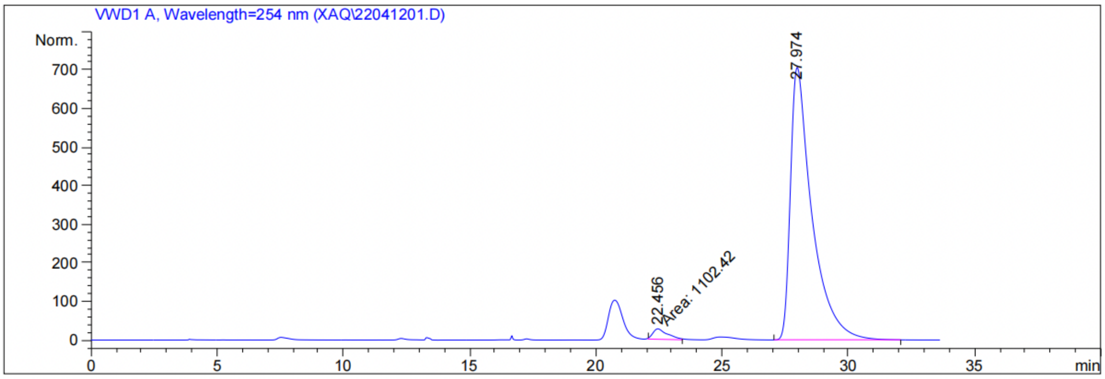

Peak #	RetTime [min]	Type	Width [min]	Area mAU *s	Height [mAU]	Area %
1	22.456	MM	0.6778	1102.42407	27.10729	2.5688
2	27.974	BB	0.8479	4.18141e4	706.38190	97.4312

Compound 3c

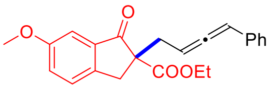

3c

Prepared according to the procedure within 48 h as light yellow liquid (68.8 mg, 95% yield, dr = 8 : 1). $[\alpha]_D^{17}$ = 71.161 (*c* 0.27, CH_2Cl_2); ^1H NMR (400 MHz, Chloroform-*d*) δ 7.40 – 7.32 (m, 4H), 7.27 (d, *J* = 1.9 Hz, 2H), 7.25 – 7.15 (m, 2H), 6.16 (dt, *J* = 5.8, 2.7 Hz, 1H), 5.55 (q, *J* = 6.9 Hz, 1H), 4.18 (q, *J* = 7.1 Hz, 2H), 3.91 (s, 3H), 3.72 (d, *J* = 17.0 Hz, 1H), 3.24 (d, *J* = 17.0 Hz, 1H), 3.06 (ddd, *J* = 14.6, 7.1, 2.8 Hz, 1H), 2.78 (ddd, *J* = 14.6, 7.2, 2.7 Hz, 1H), 1.25 (t, *J* = 7.1 Hz, 3H); ^{13}C NMR (101 MHz, Chloroform-*d*) δ 206.5, 201.9, 170.5, 159.7, 146.2, 136.5, 134.1, 128.5, 127.1, 127.0, 126.8, 124.9, 105.7, 95.4, 90.0, 61.7, 61.0, 55.6, 35.8, 34.2, 14.0. HRMS (ESI) m/z Calcd. for $C_{23}H_{22}NaO_4$ ($[M+Na]^+$) 385.1410, Found 385.1404. Enantiomeric excess was determined to be 95% (determined by HPLC using chiral OD-H column, hexane/2-propanol = 50/1, λ = 254 nm, 30 ºC, 0.8 mL/min, t_{major} = 31.3 min, t_{minor} = 35.8 min).

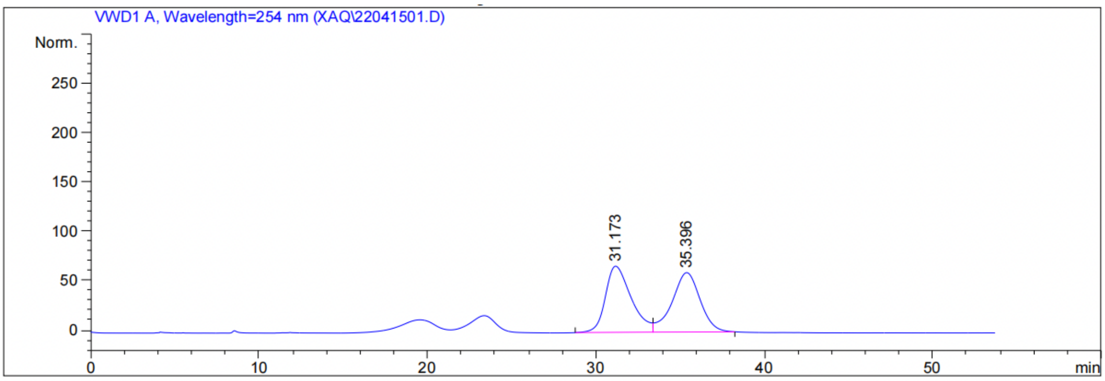

Peak #	RetTime [min]	Type	Width [min]	Area mAU *s	Height [mAU]	Area %
1	31.173	BV	1.5686	7174.93408	67.13802	50.6282
2	35.396	VB	1.6970	6996.89258	60.24957	49.3718

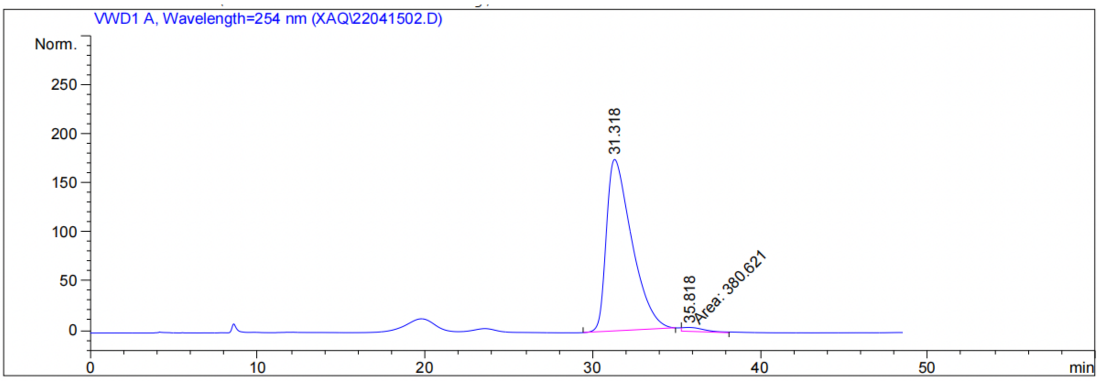

Peak #	RetTime [min]	Type	Width [min]	Area mAU *s	Height [mAU]	Area %
1	31.318	PB	1.5467	1.84605e4	174.57515	97.9798
2	35.818	MM	1.5652	380.62112	4.05298	2.0202

Compound 3d

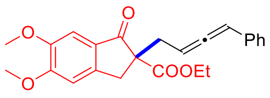

3d

Prepared according to the procedure within 72 h as light yellow liquid (77.6 mg, 99% yield, dr = 9 : 1). $[\alpha]_D^{17}$ = 100.27 (c 0.74, CH_2Cl_2); ^{1}H NMR (400 MHz, Chloroform-d) δ 7.32 – 7.22 (m, 2H), 7.20 – 7.10 (m, 4H), 6.79 (s, 1H), 6.05 (dt, J = 6.1, 2.8 Hz, 1H), 5.48 (q, J = 6.9 Hz, 1H), 4.10 (q, J = 7.1 Hz, 2H), 3.92 (s, 3H), 3.89 (s, 3H), 3.63 (d, J = 17.1 Hz, 1H), 3.20 – 3.09 (m, 1H), 2.97 (ddd, J = 14.8, 7.1, 2.7 Hz, 1H), 2.71 (ddd, J = 14.7, 7.0, 2.8 Hz, 1H), 1.18 (t, J = 7.1 Hz, 3H); ^{13}C NMR (101 MHz, Chloroform-d) δ 206.3, 200.3, 170.7, 156.0, 149.7, 148.8, 134.1, 128.5, 128.0, 126.9, 126.8, 107.3, 104.9, 95.4, 90.1, 61.7, 60.6, 56.2, 56.1, 36.1, 34.0, 14.0. HRMS (ESI) m/z Calcd. for $C_{24}H_{24}NaO_5$ ($[M+Na]^+$) 415.1516, Found 415.1507. Enantiomeric excess was determined to be 97% (determined by HPLC using chiral OD-H column, hexane/2-propanol = 7/3, λ = 254 nm, 30 °C, 0.8 mL/min, t_{major} = 25.3 min, t_{minor} = 33.9 min).

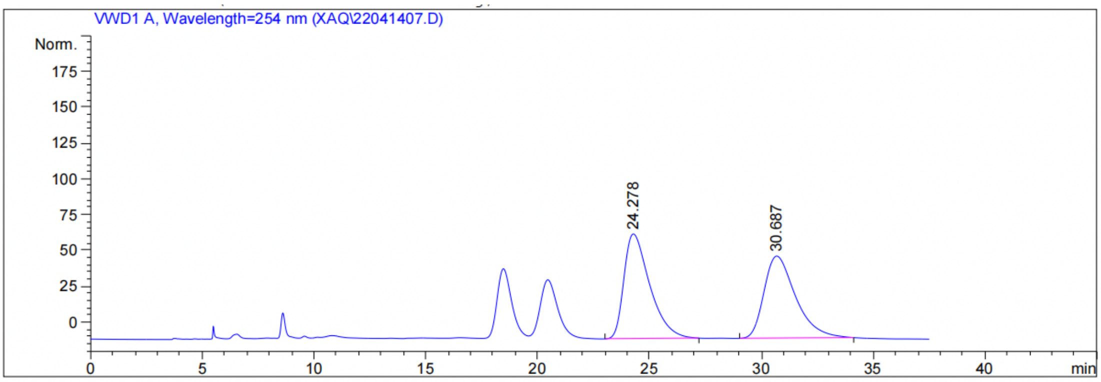

Peak #	RetTime [min]	Type	Width [min]	Area mAU *s	Height [mAU]	Area %
1	24.278	BB	1.2041	5891.83203	73.07874	51.0950
2	30.687	BB	1.4677	5639.29980	57.26864	48.9050

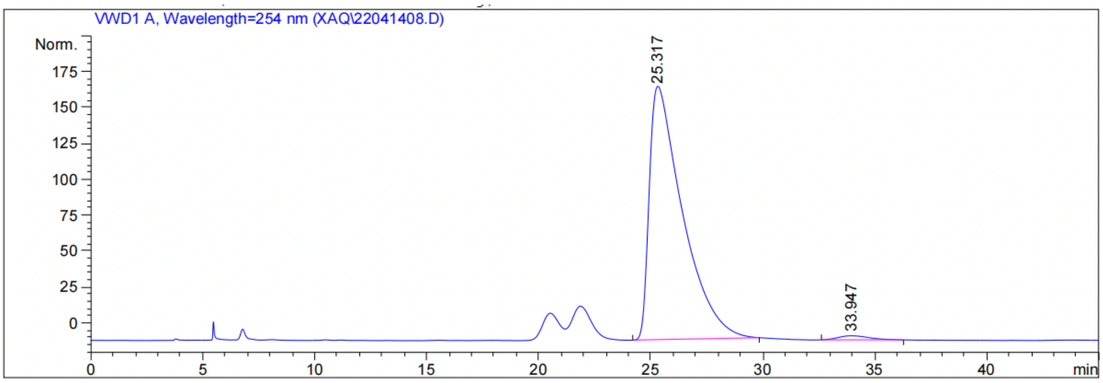

Peak #	RetTime [min]	Type	Width [min]	Area mAU *s	Height [mAU]	Area %
1	25.317	PB	1.4613	1.81929e4	176.35521	98.5053
2	33.947	BB	1.1370	276.05276	2.85450	1.4947

Compound 3e

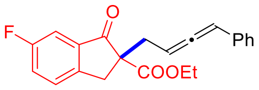

3e

Prepared according to the procedure within 72 h as light yellow liquid (69.3 mg, 99% yield, dr = 7 : 1). $[\alpha]^{18}_{D}$ = 76.074 (*c* 0.65, CH_2Cl_2); ^{1}H NMR (600 MHz, Chloroform-*d*) δ 7.37 (dd, *J* = 7.4, 2.5 Hz, 1H), 7.33 (dd, *J* = 8.5, 4.3 Hz, 1H), 7.29 – 7.22 (m, 3H), 7.20 – 7.13 (m, 3H), 6.05 (dt, *J* = 6.0, 2.7 Hz, 1H), 5.47 (q, *J* = 6.7 Hz, 1H), 4.08 (qd, *J* = 7.1, 1.2 Hz, 2H), 3.67 (d, *J* = 17.1 Hz, 1H), 3.19 (d, *J* = 17.1 Hz, 1H), 2.97 (ddd, *J* = 14.9, 7.0, 2.8 Hz, 1H), 2.73 (ddd, *J* = 14.9, 7.0, 2.8 Hz, 1H), 1.16 (t, *J* = 7.2 Hz, 3H); ^{13}C NMR (151 MHz, Chloroform-*d*) δ 206.3, 201.1, 170.1, 162.4 (d, *J* = 166.7 Hz), 148.6, 137.0, 133.9, 128.6, 127.9 (d, *J* = 5.1 Hz), 127.1, 126.8, 122.9, 110.3 (d, *J* = 15.2 Hz), 95.8, 89.9, 61.9, 61.2, 35.9, 34.0, 14.0; ^{19}F NMR (376 MHz, Chloroform-*d*) δ -104.2 – -123.6 (m). HRMS (ESI) m/z Calcd. for $C_{22}H_{19}FNaO_3$ ($[M+Na]^+$) 373.1210, Found 373.1202. Enantiomeric excess was determined to be 96% (determined by HPLC using chiral IC-IB-H column, hexane/2-propanol = 9/1, λ = 254 nm, 30 °C, 0.6 mL/min, t_{major} = 33.4 min, t_{minor} = 32.0 min).

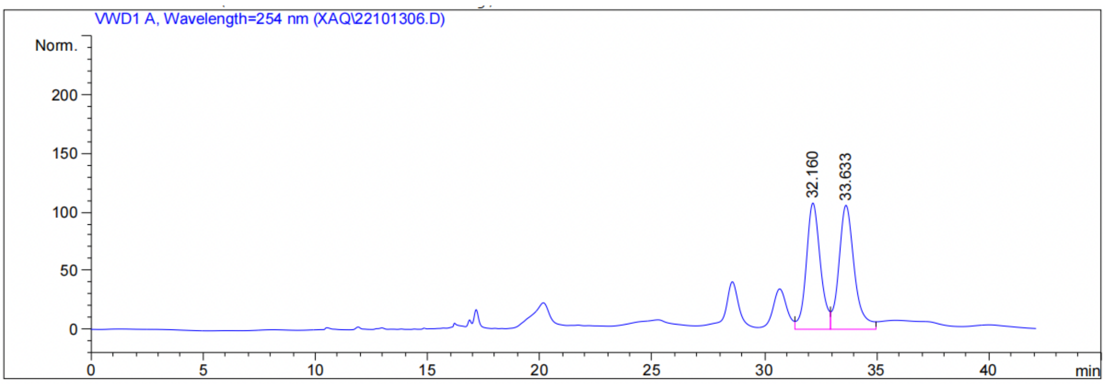

Peak #	RetTime [min]	Type	Width [min]	Area mAU *s	Height [mAU]	Area %
1	32.160	VV	0.6629	4710.12256	107.73920	48.0619
2	33.633	VV	0.7259	5089.98584	105.78870	51.9381

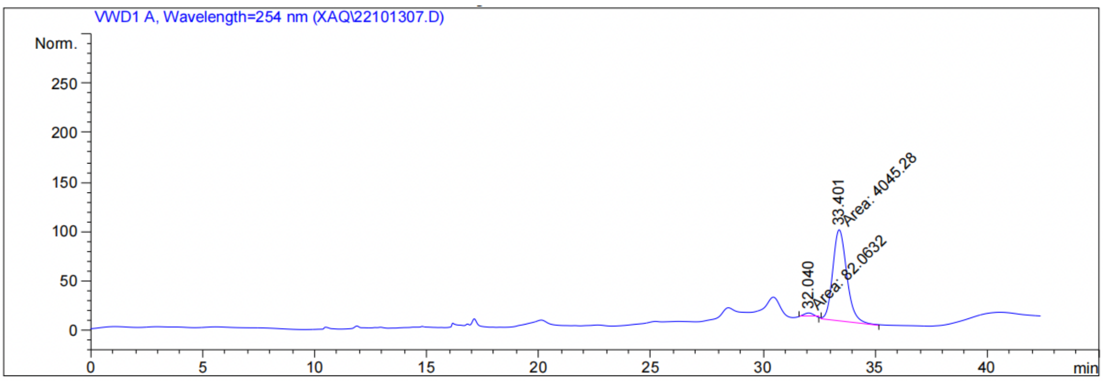

Peak #	RetTime [min]	Type	Width [min]	Area mAU *s	Height [mAU]	Area %
1	32.040	MM	0.4285	82.06321	3.19188	1.9883
2	33.401	MM	0.7299	4045.28101	92.36564	98.0117

Compound 3f

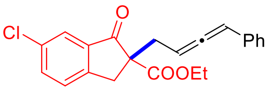

3f

Prepared according to the procedure within 96 h as light yellow liquid (72.5 mg, 99% yield, dr = 8 : 1). $[\alpha]_D^{16}$ = 104.13 (*c* 0.70, CH_2Cl_2); ^{1}H NMR (600 MHz, Chloroform-*d*) δ 7.69 (d, *J* = 2.1 Hz, 1H), 7.47 (dd, *J* = 8.1, 2.1 Hz, 1H), 7.29 (d, *J* = 8.2 Hz, 1H), 7.28 – 7.24 (m, 2H), 7.20-7.16 (m, 1H), 7.15 – 7.12 (m, 2H), 6.04 (dt, *J* = 6.0, 2.7 Hz, 1H), 5.46 (q, *J* = 6.7 Hz, 1H), 4.08 (q, *J* = 7.1 Hz, 2H), 3.67 (d, *J* = 17.4 Hz, 1H), 3.18 (d, *J* = 17.4 Hz, 1H), 2.96 (ddd, *J* = 15.0, 7.0, 2.7 Hz, 1H), 2.74 (ddd, *J* = 14.9, 6.9, 2.8 Hz, 1H), 1.15 (t, *J* = 7.1 Hz, 3H); ^{13}C NMR (151 MHz, Chloroform-*d*) δ 206.2, 200.7, 170.1, 151.3, 136.9, 135.2, 134.1, 133.8, 128.5, 127.6, 127.1, 126.8, 124.3, 95.9, 89.8, 62.0, 60.8, 36.0, 34.0, 14.0. HRMS (ESI) m/z Calcd. for $C_{22}H_{19}ClNaO_3$ ($[M+Na]^+$) 389.0915, Found 389.0911. Enantiomeric excess was determined to be 97% (determined by HPLC using chiral AD-H column, hexane/2-propanol = 50/1, λ = 254 nm, 30 °C, 0.8 mL/min, t_{major} = 37.9 min, t_{minor} = 30.4 min).

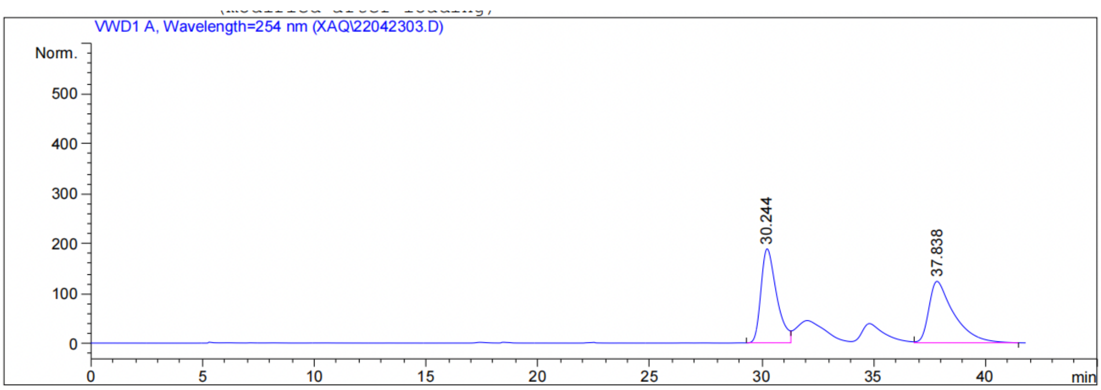

Peak #	RetTime [min]	Type	Width [min]	Area mAU *s	Height [mAU]	Area %
1	30.244	BV	0.7205	9166.04199	187.90143	49.0342
2	37.838	BB	1.1028	9527.13086	122.84808	50.9658

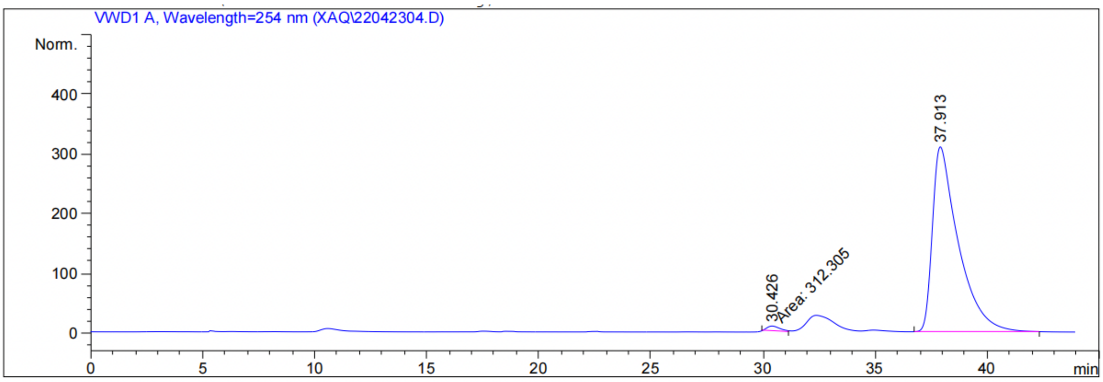

Peak #	RetTime [min]	Type	Width [min]	Area mAU *s	Height [mAU]	Area %
1	30.426	MM	0.6603	312.30530	7.88311	1.2377
2	37.913	BB	1.1396	2.49198e4	310.19211	98.7623

Compound 3g

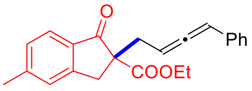

3g

Prepared according to the procedure within 72 h as light yellow liquid (66.5 mg, 96% yield, dr = 6 : 1); $[\alpha]_D^{16}$ = 69.372 (*c* 0.38, CH_2Cl_2); ^{1}H NMR (400 MHz, Chloroform-*d*) δ 7.65 (d, *J* = 7.8 Hz, 1H), 7.30 – 7.23 (m, 2H), 7.18 (d, *J* = 7.1 Hz, 5H), 6.06 (dt, *J* = 5.9, 2.8 Hz, 1H), 5.47 (q, *J* = 6.9 Hz, 1H), 4.08 (q, *J* = 7.1 Hz, 2H), 3.68 (d, *J* = 17.4 Hz, 1H), 3.17 (d, *J* = 17.3 Hz, 1H), 2.98 (ddd, *J* = 14.7, 7.0, 2.8 Hz, 1H), 2.67 (ddd, *J* = 14.6, 7.2, 2.7 Hz, 1H), 2.40 (s, 3H), 1.16 (t, *J* = 7.1 Hz, 3H); ^{13}C NMR (101 MHz, Chloroform-*d*) δ 206.4, 201.3, 170.6, 153.8, 146.8, 143.3, 134.1, 133.0, 129.1, 128.5, 127.0, 126.8, 124.5, 95.4, 90.1, 61.7, 60.4, 36.3, 34.2, 22.1, 14.0. HRMS (ESI) m/z Calcd. for $C_{23}H_{22}NaO_3$ ($[M+Na]^+$) 369.1461, Found 369.1455. Enantiomeric excess was determined to be 96% (determined by HPLC using chiral AD-H column, hexane/2-propanol = 50/1, λ = 254 nm, 30 ºC, 0.8 mL/min, t_{major} = 33.3 min, t_{minor} = 30.3 min).

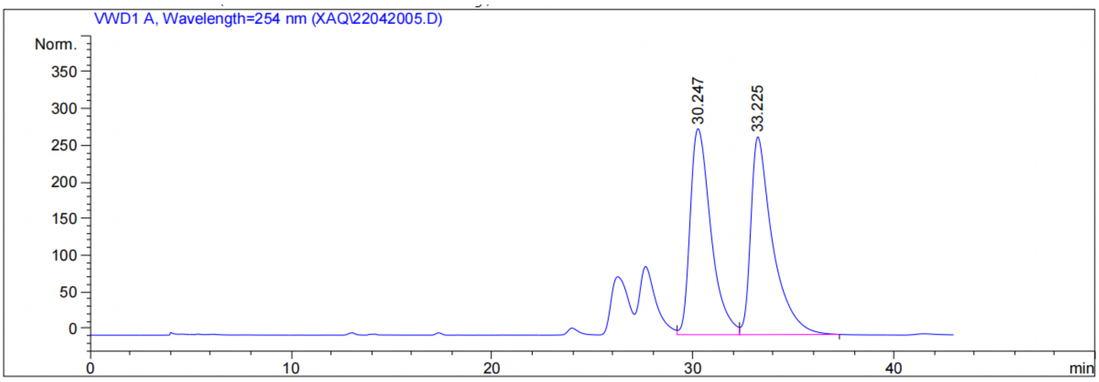

Peak #	RetTime [min]	Type	Width [min]	Area mAU *s	Height [mAU]	Area %
1	30.247	VV	1.0984	2.02312e4	281.24133	49.7153
2	33.225	VB	1.0808	2.04629e4	269.99170	50.2847

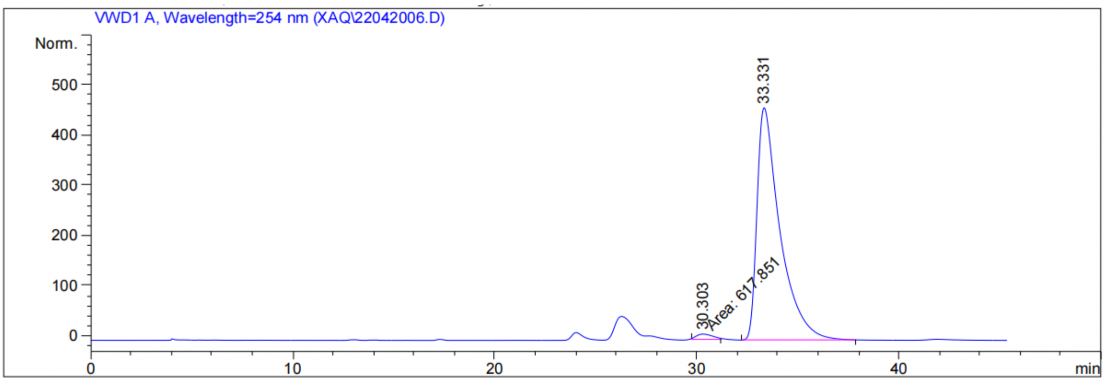

Peak #	RetTime [min]	Type	Width [min]	Area mAU *s	Height [mAU]	Area %
1	30.303	MM	0.9564	617.85095	10.76710	1.6614
2	33.331	BB	1.1400	3.65705e4	462.95499	98.3386

Compound 3h

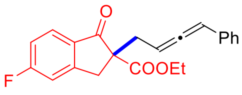

3h

Prepared according to the procedure within 96 h as light yellow liquid (67.2 mg, 96% yield, dr = 10 : 1). $[\alpha]_D^{17}$ = 84.648 (*c* 0.47, CH_2Cl_2); ^{1}H NMR (600 MHz, Chloroform-*d*) δ 7.75 (dd, *J* = 8.4, 5.2 Hz, 1H), 7.31 – 7.25 (m, 2H), 7.22 – 7.15 (m, 3H), 7.08 – 7.01 (m, 2H), 6.06 (dt, *J* = 6.0, 2.8 Hz, 1H), 5.47 (q, *J* = 6.8 Hz, 1H), 4.09 (q, *J* = 7.1 Hz, 2H), 3.71 (d, *J* = 17.5 Hz, 1H), 3.20 (d, *J* = 17.6 Hz, 1H), 2.98 (ddd, *J* = 14.9, 7.0, 2.8 Hz, 1H), 2.70 (ddd, *J* = 14.9, 6.9, 2.7 Hz, 1H), 1.17 (t, *J* = 7.1 Hz, 3H); ^{13}C NMR (151 MHz, Chloroform-*d*) δ 206.3, 199.9, 170.1, 167.5 (d, *J* = 257.6 Hz), 156.2 (d, *J* = 9.9 Hz), 133.9, 131.7, 128.6, 127.1, 127.0 (d, *J* = 10.8 Hz), 126.8, 116.2 (d, *J* = 23.9 Hz), 113.2 (d, *J* = 22.7 Hz), 95.7, 89.9, 61.9, 60.6, 36.2, 34.0, 14.0; ^{19}F NMR (377 MHz, Chloroform-*d*) δ -101.5 (t, *J* = 9.4 Hz). HRMS (ESI) m/z Calcd. for $C_{22}H_{19}FNaO_3$ ($[M+Na]^+$) 373.1210, Found 373.1202. Enantiomeric excess was determined to be 96% (determined by HPLC using chiral AD-H column, hexane/2-propanol = 50/1, λ = 254 nm, 30 °C, 0.8 mL/min, t_{major} = 26.1 min, t_{minor} = 24.0 min).

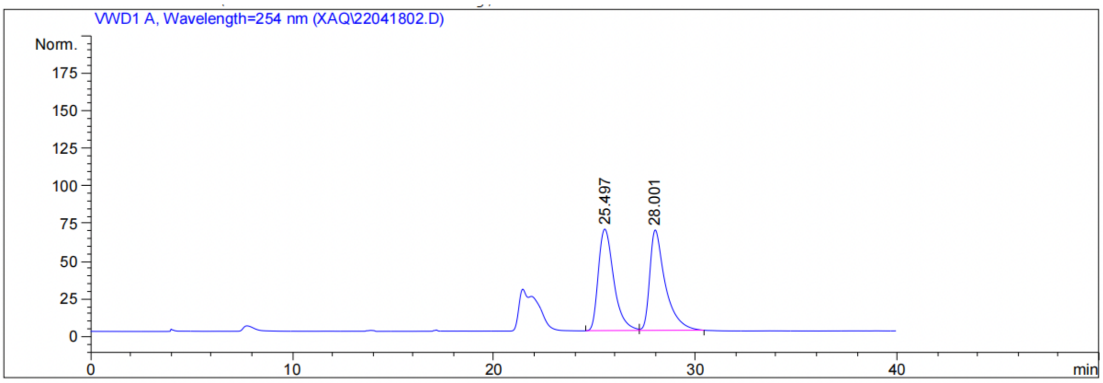

Peak #	RetTime [min]	Type	Width [min]	Area mAU *s	Height [mAU]	Area %
1	25.497	BB	0.8315	3607.89868	67.52844	50.1612
2	28.001	BB	0.7804	3584.70630	66.80284	49.8388

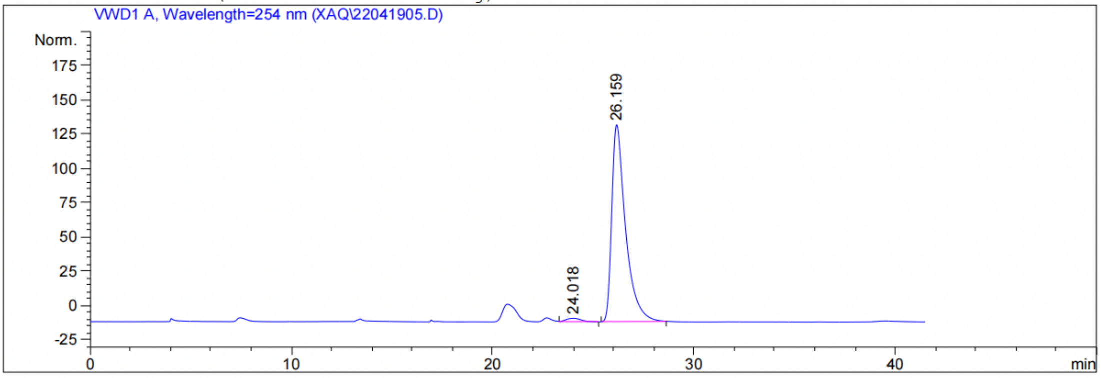

Peak #	RetTime [min]	Type	Width [min]	Area mAU *s	Height [mAU]	Area %
1	24.018	VP	0.6444	134.60893	2.56444	1.9276
2	26.159	BB	0.6973	6848.59326	143.68753	98.0724

Compound 3i

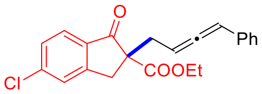

3i

Prepared according to the procedure within 48 h as light yellow liquid (72.5 mg, 99% yield, dr = 11 : 1). $[\alpha]^{18}_{D}$ = 69.762 (*c* 0.51, CH_2Cl_2); ^{1}H NMR (400 MHz, Chloroform-*d*) δ 7.70 (d, *J* = 8.1 Hz, 1H), 7.51 – 7.41 (m, 1H), 7.35 (dd, *J* = 10.2, 2.0 Hz, 2H), 7.28 (d, *J* = 1.8 Hz, 1H), 7.22 (d, *J* = 7.2 Hz, 1H), 7.20 – 7.15 (m, 2H), 6.07 (dt, *J* = 6.0, 2.9 Hz, 1H), 5.49 (q, *J* = 6.6 Hz, 1H), 4.11 (q, *J* = 7.1 Hz, 2H), 3.71 (d, *J* = 17.5 Hz, 1H), 3.22 (d, *J* = 17.5 Hz, 1H), 2.99 (ddd, *J* = 14.9, 7.0, 2.8 Hz, 1H), 2.75 (ddd, *J* = 14.9, 6.8, 2.8 Hz, 1H), 1.19 (t, *J* = 7.1 Hz, 3H); ^{13}C NMR (101 MHz, Chloroform-*d*) δ 206.3, 200.4, 170.1, 154.6, 141.9, 133.9, 133.8, 128.6, 128.6, 127.2, 126.7, 126.7, 125.7, 95.9, 89.8, 61.9, 60.5, 36.1, 33.9, 14.0. HRMS (ESI) m/z Calcd. for $C_{22}H_{19}ClNaO_3$ ($[M+Na]^+$) 389.0915, Found 389.0908. Enantiomeric excess was determined to be 95% (determined by HPLC using chiral AD-H column, hexane/2-propanol = 50/1, λ = 254 nm, 30 °C, 0.8 mL/min, t_{major} = 25.3 min, t_{minor} = 22.8 min).

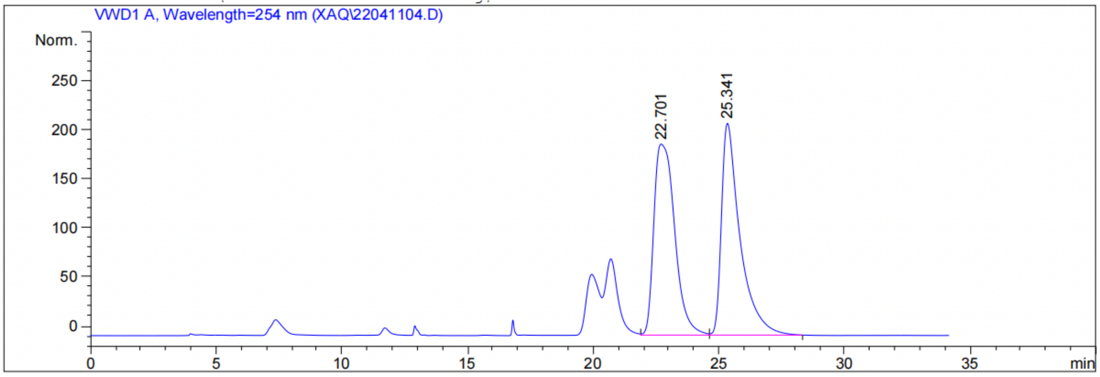

Peak #	RetTime [min]	Type	Width [min]	Area mAU *s	Height [mAU]	Area %
1	22.701	VV	0.9185	1.12684e4	194.66626	50.3950
2	25.341	VB	0.7410	1.10917e4	215.75056	49.6050

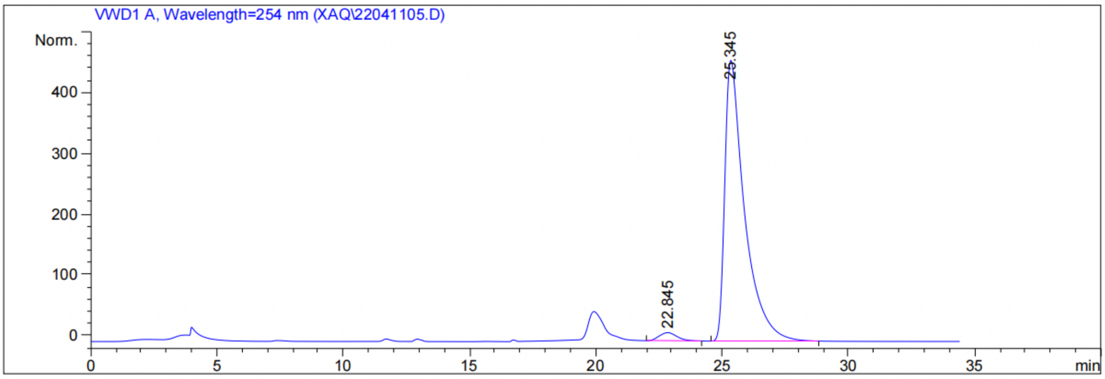

Peak #	RetTime [min]	Type	Width [min]	Area mAU *s	Height [mAU]	Area %
1	22.845	BB	0.7705	652.11298	13.39276	2.5389
2	25.345	PB	0.7860	2.50329e4	461.28653	97.4611

Compound 3j

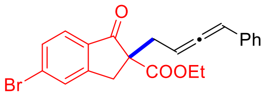

3j

Prepared according to the procedure within 48 h as light yellow liquid (81.2 mg, 99% yield, dr = 12 : 1). $[\alpha]^{16}_{D}$ = 48.041 (*c* 0.69, CH_2Cl_2); ^1H NMR (400 MHz, Chloroform-*d*) δ 7.59 (d, *J* = 8.2 Hz, 1H), 7.54 – 7.46 (m, 2H), 7.30 – 7.24 (m, 2H), 7.22 – 7.12 (m, 3H), 6.03 (dt, *J* = 6.1, 2.8 Hz, 1H), 5.46 (q, *J* = 6.7 Hz, 1H), 4.08 (q, *J* = 7.1 Hz, 2H), 3.68 (d, *J* = 17.5 Hz, 1H), 3.19 (d, *J* = 17.5 Hz, 1H), 2.96 (ddd, *J* = 14.9, 6.9, 2.8 Hz, 1H), 2.73 (ddd, *J* = 14.9, 6.8, 2.9 Hz, 1H), 1.16 (t, *J* = 7.1 Hz, 3H); ^{13}C NMR (101 MHz, Chloroform-*d*) δ 206.2, 200.7, 170.0, 154.7, 134.3, 133.8, 131.4, 130.8, 129.8, 128.6, 127.2, 126.7, 125.7, 95.9, 89.8, 62.0, 60.4, 36.0, 33.9, 14.0. HRMS (ESI) m/z Calcd. for $C_{22}H_{19}BrNaO_3$ ($[M+Na]^+$) 433.0410, Found 433.0403. Enantiomeric excess was determined to be 97% (determined by HPLC using chiral AD-H column, hexane/2-propanol = 50/1, λ = 254 nm, 30 °C, 0.8 mL/min, t_{major} = 27.2min, t_{minor} = 24.8 min).

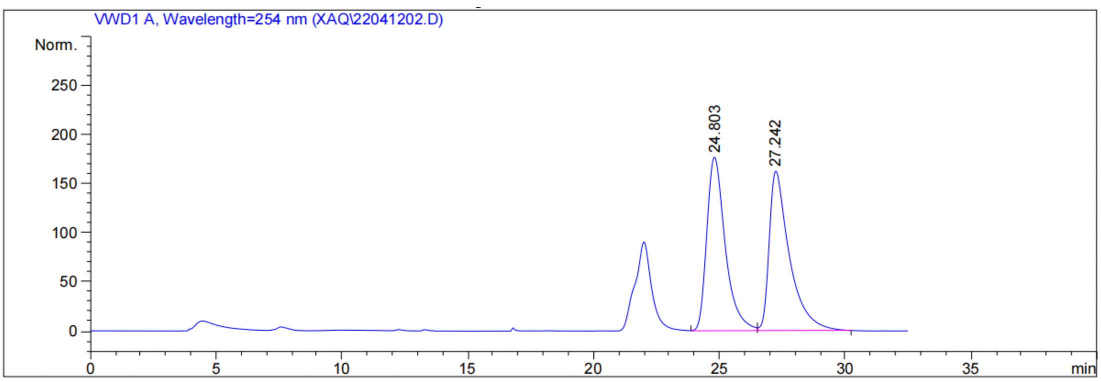

Peak #	RetTime [min]	Type	Width [min]	Area mAU *s	Height [mAU]	Area %
1	24.803	BV	0.7751	9018.91211	176.54369	49.7866
2	27.242	VB	0.8145	9096.20996	162.18997	50.2134

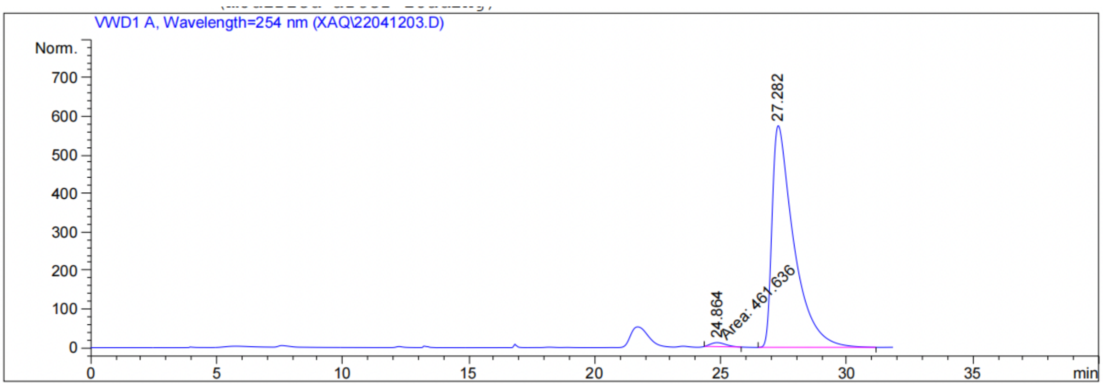

Peak #	RetTime [min]	Type	Width [min]	Area mAU *s	Height [mAU]	Area %
1	24.864	MM	0.7140	461.63571	10.77601	1.3163
2	27.282	BB	0.8719	3.46093e4	575.56549	98.6837

Compound 3k

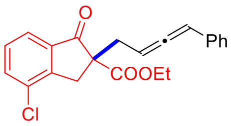

3k

Prepared according to the procedure within 60 h as light yellow liquid (72.5 mg, 99% yield, dr = 9 : 1). $[\alpha]_D^{15}$ = 227.30 (*c* 0.67, CH_2Cl_2); ^{1}H NMR (600 MHz, Chloroform-*d*) δ 7.65 (d, *J* = 7.6 Hz, 1H), 7.52 (d, *J* = 7.7 Hz, 1H), 7.33-7.30 (m, 1H), 7.29 – 7.23 (m, 2H), 7.20 – 7.13 (m, 3H), 6.06 (dt, *J* = 6.0, 2.7 Hz, 1H), 5.48 (q, *J* = 6.8 Hz, 1H), 4.10 (qd, *J* = 7.2, 2.4 Hz, 2H), 3.69 (d, *J* = 17.8 Hz, 1H), 3.23 (d, *J* = 17.8 Hz, 1H), 3.00 (ddd, *J* = 14.8, 6.7, 2.8 Hz, 1H), 2.70 (ddd, *J* = 14.9, 7.2, 2.7 Hz, 1H), 1.17 (t, *J* = 7.1 Hz, 3H); ^{13}C NMR (101 MHz, Chloroform-*d*) δ 206.4, 201.1, 170.0, 150.7, 137.3, 134.8, 133.8, 132.8, 129.3, 128.5, 127.1, 126.8, 122.8, 95.9, 89.8, 62.0, 60.2, 35.6, 34.1, 14.0. HRMS (ESI) m/z Calcd. for $C_{22}H_{19}ClNaO_3$ ($[M+Na]^+$) 389.0915, Found 389.0908. Enantiomeric excess was determined to be 95% (determined by HPLC using chiral AD-OD-H column, hexane/2-propanol = 50/1, λ = 254 nm, 30 °C, 0.6 mL/min, t_{major} = 47.7 min, t_{minor} = 51.0 min).

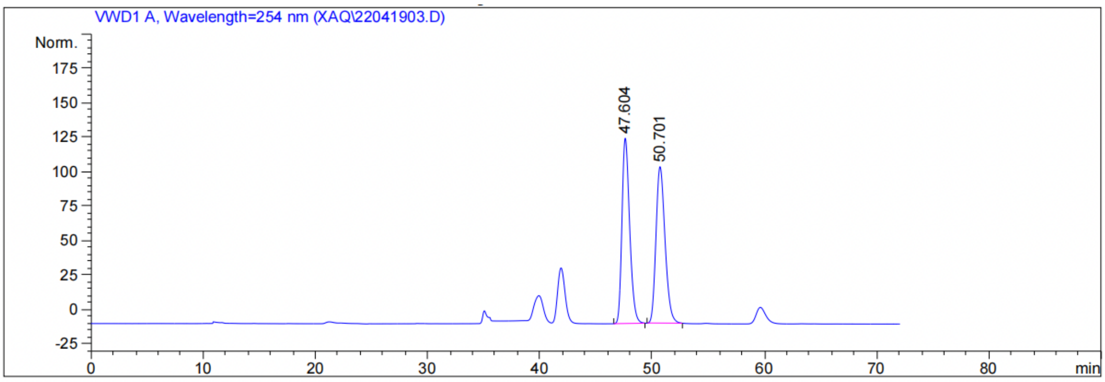

Peak #	RetTime [min]	Type	Width [min]	Area mAU *s	Height [mAU]	Area %
1	47.604	BB	0.7325	6419.30176	134.62816	50.0734
2	50.701	BB	0.8722	6400.48242	113.71004	49.9266

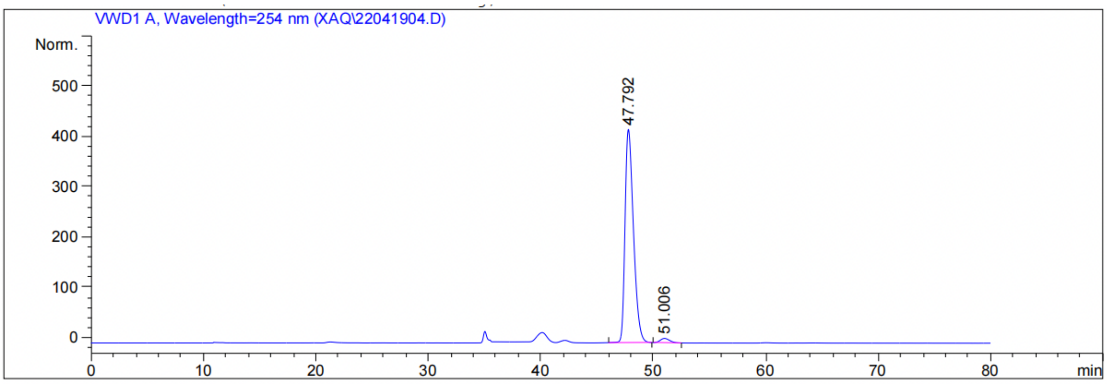

Peak #	RetTime [min]	Type	Width [min]	Area mAU *s	Height [mAU]	Area %
1	47.792	BB	0.7994	2.20036e4	423.77588	97.8261
2	51.006	PB	0.7853	488.96561	8.51138	2.1739

Compound 3l

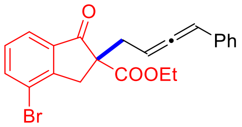

3l

Prepared according to the procedure within 72 h as light yellow liquid (81.2 mg, 99% yield, dr = 8 : 1). $[\alpha]_D^{16}$ = 76.255 (c 0.74, CH_2Cl_2); ^1H NMR (400 MHz, Chloroform-d) δ 7.69 (d, J = 7.7 Hz, 2H), 7.29 – 7.22 (m, 3H), 7.21 – 7.14 (m, 3H), 6.06 (dt, J = 6.1, 2.8 Hz, 1H), 5.48 (q, J = 6.8 Hz, 1H), 4.11 (qd, J = 7.1, 1.5 Hz, 2H), 3.64 (d, J = 17.9 Hz, 1H), 3.18 (d, J = 17.9 Hz, 1H), 3.00 (ddd, J = 14.8, 6.7, 2.9 Hz, 1H), 2.69 (ddd, J = 14.8, 7.3, 2.7 Hz, 1H), 1.18 (t, J = 7.2 Hz, 3H); ^{13}C NMR (101 MHz, Chloroform-d) δ 206.4, 201.2, 170.0, 152.8, 138.0, 137.3, 133.8, 129.5, 128.6, 127.1, 126.8, 123.5, 122.0, 95.9, 89.8, 62.0, 60.3, 37.6, 34.2, 14.0. HRMS (ESI) m/z Calcd. for $C_{22}H_{19}BrNaO_3$ ($[M+Na]^+$) 433.0410, Found 433.0404. Enantiomeric excess was determined to be 97% (determined by HPLC using chiral AD-OD-H column, hexane/2-propanol = 50/1, λ = 254 nm, 30 °C, 0.6 mL/min, t_{major} = 48.8 min, t_{minor} = 52.0 min).

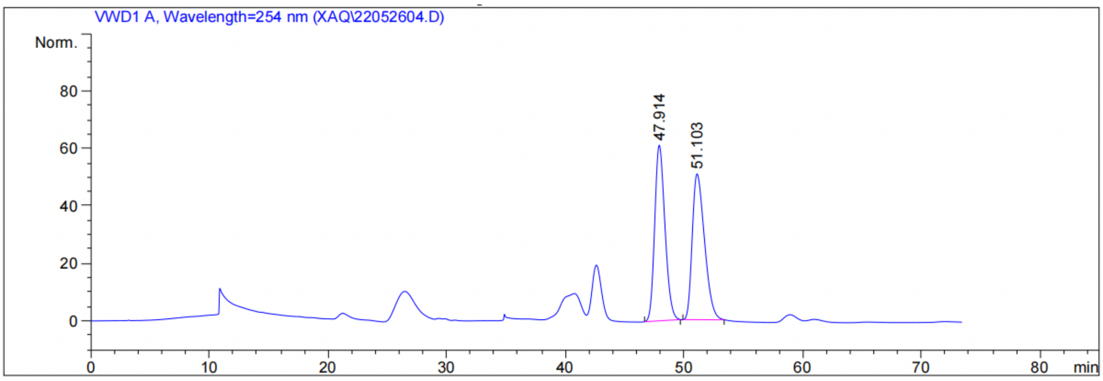

Peak #	RetTime [min]	Type	Width [min]	Area mAU *s	Height [mAU]	Area %
1	47.914	BB	0.9317	3689.23511	61.21368	50.1976
2	51.103	BB	1.0908	3660.19727	50.81617	49.8024

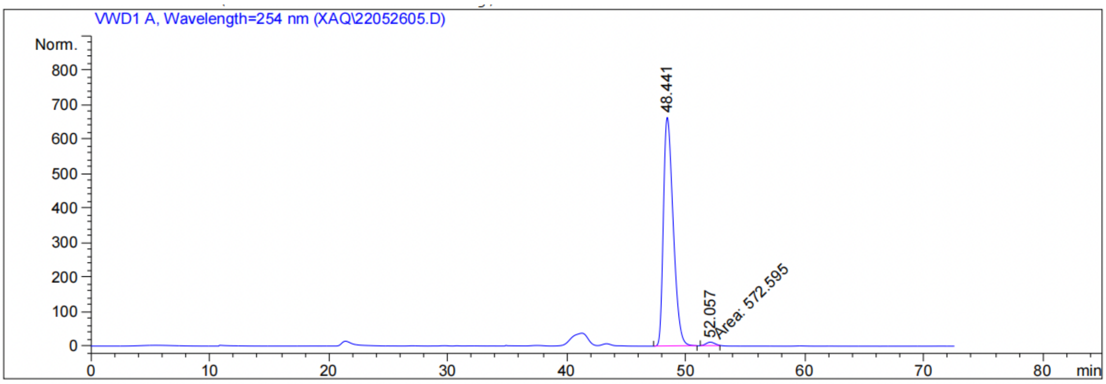

Peak #	RetTime [min]	Type	Width [min]	Area mAU *s	Height [mAU]	Area %
1	48.441	BB	0.8795	3.79183e4	663.34723	98.5124
2	52.057	MM	0.9055	572.59546	10.53911	1.4876

Compound 3m

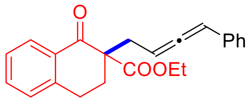

3m

Prepared according to the procedure within 48 h as light yellow liquid (68.5 mg, 99% yield, dr = 2 : 1). $[\alpha]_D^{16}$ = 9.667 (*c* 0.30, CH_2Cl_2); ^{1}H NMR (400 MHz, Chloroform-*d*) δ 8.05 (dd, *J* = 7.9, 1.5 Hz, 1H), 7.51 – 7.38 (m, 1H), 7.35 – 7.21 (m, 5H), 7.21-7.17 (m, 2H), 6.12 (dt, *J* = 6.5, 2.3 Hz, 1H), 5.60 (q, *J* = 7.4 Hz, 1H), 4.16 (qt, *J* = 7.1, 1.4 Hz, 2H), 3.11 – 2.86 (m, 2H), 2.79 (ddd, *J* = 8.1, 5.5, 2.4 Hz, 2H), 2.62 (dt, *J* = 13.9, 5.5 Hz, 1H), 2.34 (ddd, *J* = 14.1, 9.5, 5.0 Hz, 1H), 1.17 (t, *J* = 7.1Hz, 3H); ^{13}C NMR (101 MHz, Chloroform-*d*) δ 207.0, 195.0, 171.5, 143.2, 134.3, 133.6, 131.9, 128.8, 128.6, 128.4, 128.1, 127.0, 126.8, 94.7, 90.2, 61.5, 57.5, 33.8, 30.4, 25.7, 14.1. HRMS (ESI) m/z Calcd. for $C_{23}H_{22}NaO_3$ ($[M+Na]^+$) 369.1461, Found 369.1454. Enantiomeric excess was determined to be 97%/75% (determined by HPLC using chiral OJ-H column, hexane/2-propanol = 50/1, λ = 254 nm, 30 °C, 0.8 mL/min, t_{major} = 76.9/68.2 min, t_{minor} = 57.7/82.0 min).

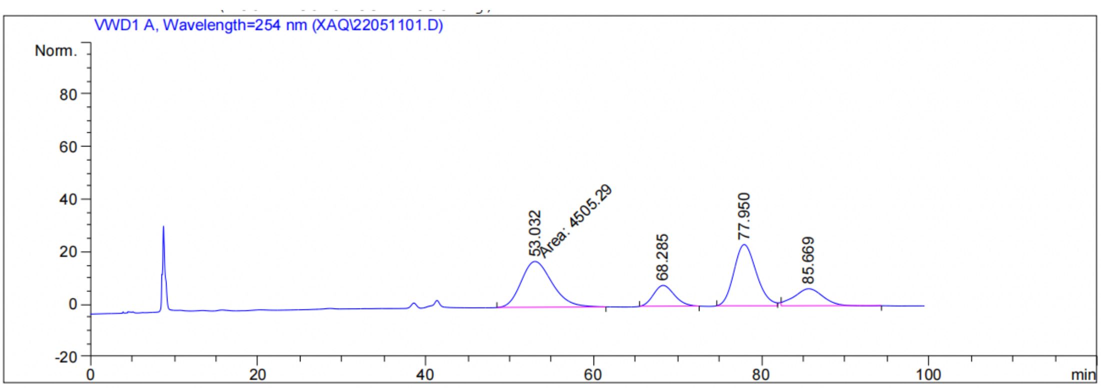

Peak #	RetTime [min]	Type	Width [min]	Area mAU *s	Height [mAU]	Area %
1	53.032	MM	4.2964	4505.28516	17.47685	38.3553
2	68.285	BB	2.0295	1376.68677	7.94235	11.7203
3	77.950	BV	2.1912	4337.10498	23.34061	36.9235
4	85.669	BP	2.7526	1527.10132	6.49299	13.0008

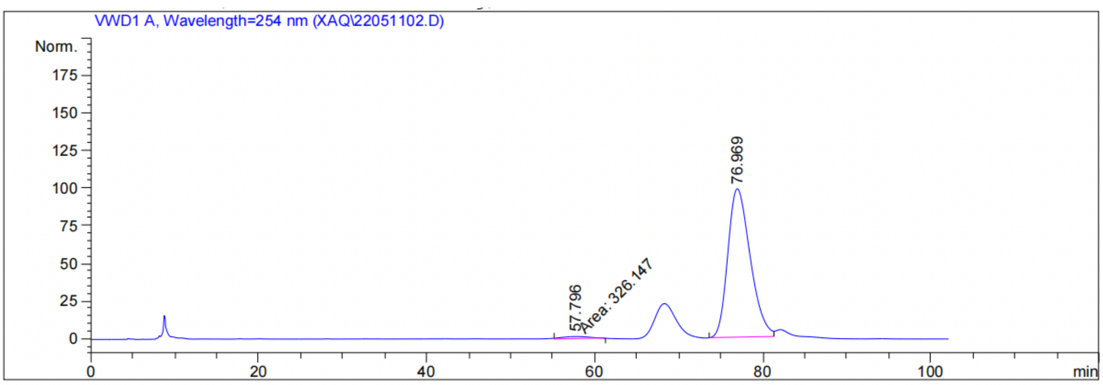

Peak #	RetTime [min]	Type	Width [min]	Area mAU *s	Height [mAU]	Area %
1	57.796	MM	3.7865	326.14658	1.43557	1.7499
2	76.969	BV	2.6782	1.83121e4	98.71461	98.2501

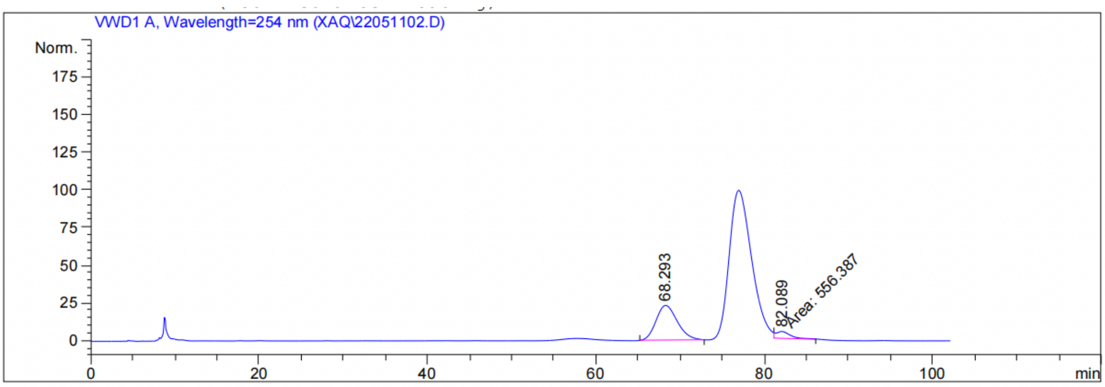

Peak #	RetTime [min]	Type	Width [min]	Area mAU *s	Height [mAU]	Area %
1	68.293	BB	2.0651	4053.25439	23.02895	87.9299
2	82.089	MM	2.0477	556.38739	4.52846	12.0701

Compound 3n

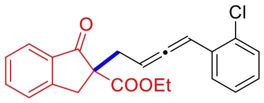

3n

Prepared according to the procedure within 72 h as light yellow liquid (58.6 mg, 80% yield, dr = 10 : 1). $[\alpha]_D^{15}$ = 300.81 (c 0.25, CH_2Cl_2); ^{1}H NMR (400 MHz, Chloroform-*d*) δ 7.78-7.76 (m, 1H), 7.61-7.57 (m, 1H), 7.46 – 7.33 (m, 3H), 7.29 (dd, *J* = 8.0, 1.4 Hz, 1H), 7.21-7.16 (m, 1H), 7.13-7.08 (m, 1H), 6.54 (dt, *J* = 6.5, 2.7 Hz, 1H), 5.50 (q, *J* = 7.0 Hz, 1H), 4.09 (qd, *J* = 7.1, 1.4 Hz, 2H), 3.73 (d, *J* = 17.4 Hz, 1H), 3.22 (d, *J* = 17.3 Hz, 1H), 2.99 (ddd, *J* = 14.7, 7.2, 2.8 Hz, 1H), 2.73 (ddd, *J* = 14.6, 7.1, 2.8 Hz, 1H), 1.16 (t, *J* = 7.1 Hz, 3H); ^{13}C NMR (101 MHz, Chloroform-*d*) δ 207.3, 201.8, 170.4, 153.2, 135.4, 135.3, 132.1, 131.8, 129.7, 128.3, 128.0, 127.8, 126.7, 126.4, 124.8, 91.8, 90.2, 61.8, 60.2, 36.5, 33.9, 14.0. HRMS (ESI) m/z Calcd. for $C_{22}H_{19}ClNaO_3$ ($[M+Na]^+$) 389.0915, Found 389.0912. Enantiomeric excess was determined to be 97% (determined by HPLC using chiral OJ-H column, hexane/2-propanol = 50/1, λ = 254 nm, 30 ºC, 0.8 mL/min, t_{major} = 48.5 min, t_{minor} = 38.6 min).

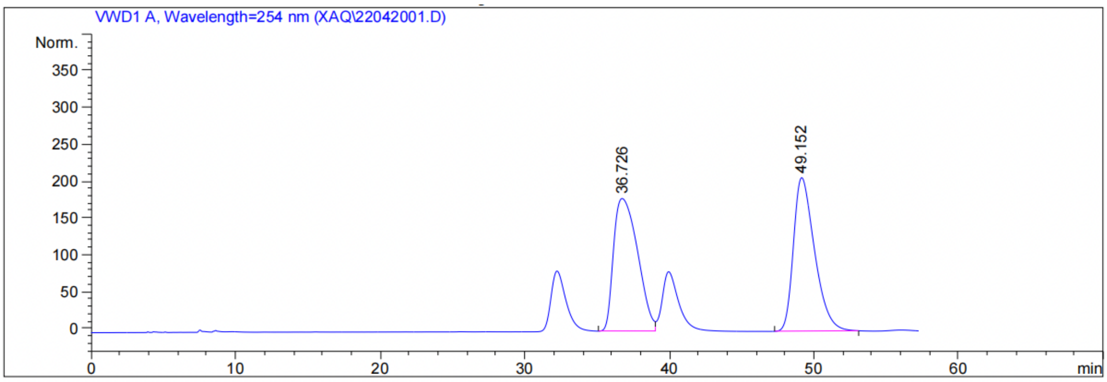

Peak #	RetTime [min]	Type	Width [min]	Area mAU *s	Height [mAU]	Area %
1	36.726	PV	1.8920	2.08295e4	179.95653	49.5341
2	49.152	BB	1.5231	2.12213e4	208.36488	50.4659

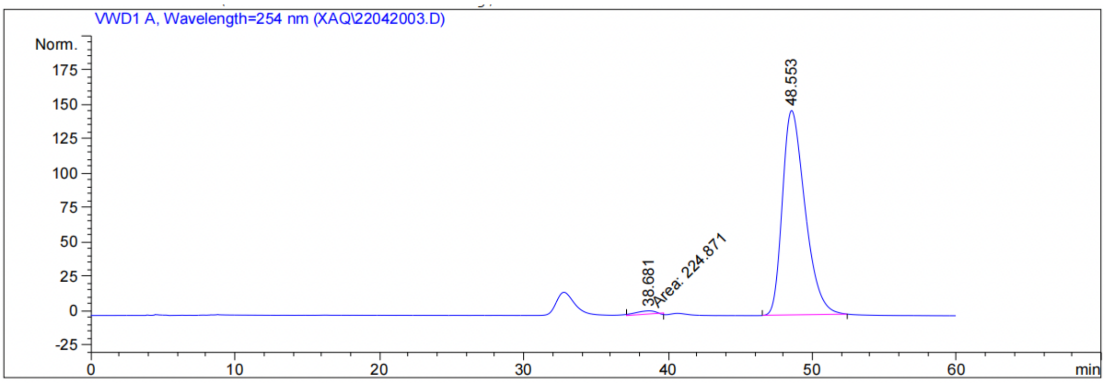

Peak #	RetTime [min]	Type	Width [min]	Area mAU *s	Height [mAU]	Area %
1	38.681	MM	1.1659	224.87071	2.30580	1.3634
2	48.553	BB	1.6435	1.62680e4	148.84500	98.6366

Compound 3o

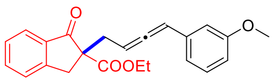

3o

Prepared according to the procedure within 48 h as light yellow liquid (71.6 mg, 99% yield, dr = 9 : 1). $[\alpha]_D^{21}$ = 64.833 (c 0.51, CH_2Cl_2); ^{1}H NMR (400 MHz, Chloroform-*d*) δ 7.76 (d, J = 7.7 Hz, 1H), 7.59-7.55 (m, 1H), 7.45 – 7.33 (m, 2H), 7.20-7.16 (m, 1H), 6.86 – 6.68 (m, 3H), 6.05 (dt, J = 6.1, 2.8 Hz, 1H), 5.48 (q, J = 6.8 Hz, 1H), 4.09 (qd, J = 7.2, 1.1 Hz, 2H), 3.81 (s, 3H), 3.74 (d, J = 17.3 Hz, 1H), 3.24 (d, J = 17.4 Hz, 1H), 3.00 (ddd, J = 14.7, 6.9, 2.9 Hz, 1H), 2.67 (ddd, J = 14.7, 7.2, 2.6 Hz, 1H), 1.16 (t, J = 7.1 Hz, 3H); ^{13}C NMR (101 MHz, Chloroform-*d*) δ 206.5, 201.9, 170.4, 159.9, 153.2, 135.5, 135.4, 135.2, 129.5, 127.8, 126.5, 124.8, 119.5, 113.0, 111.8, 95.5, 90.2, 61.8, 60.3, 55.2, 36.5, 34.2, 14.0. HRMS (ESI) m/z Calcd. for $C_{23}H_{22}NaO_4$ ($[M+Na]^+$) 385.1410, Found 385.1407. Enantiomeric excess was determined to be 97% (determined by HPLC using chiral IB-H column, hexane/2-propanol = 50/1, λ = 254 nm, 30 °C, 0.8 mL/min, t_{major} = 30.8 min, t_{minor} = 26.8 min).

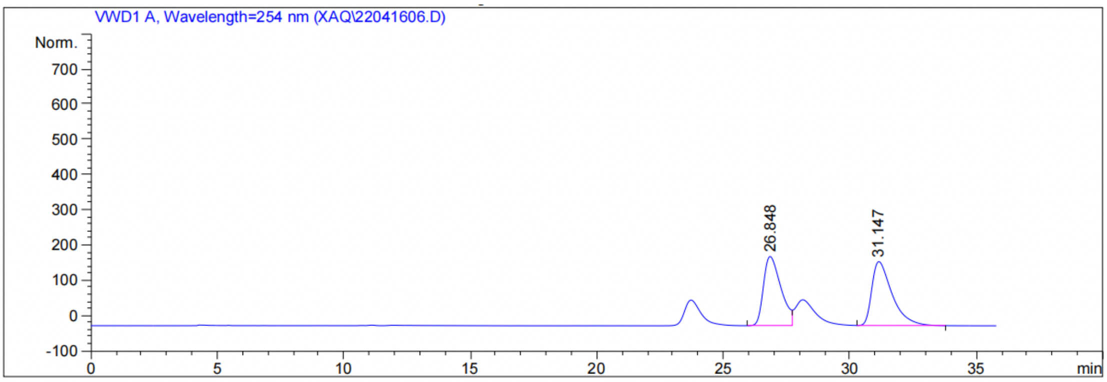

Peak #	RetTime [min]	Type	Width [min]	Area mAU *s	Height [mAU]	Area %
1	26.848	BV	0.7339	9445.51660	196.54930	48.1381
2	31.147	BB	0.8447	1.01762e4	181.94124	51.8619

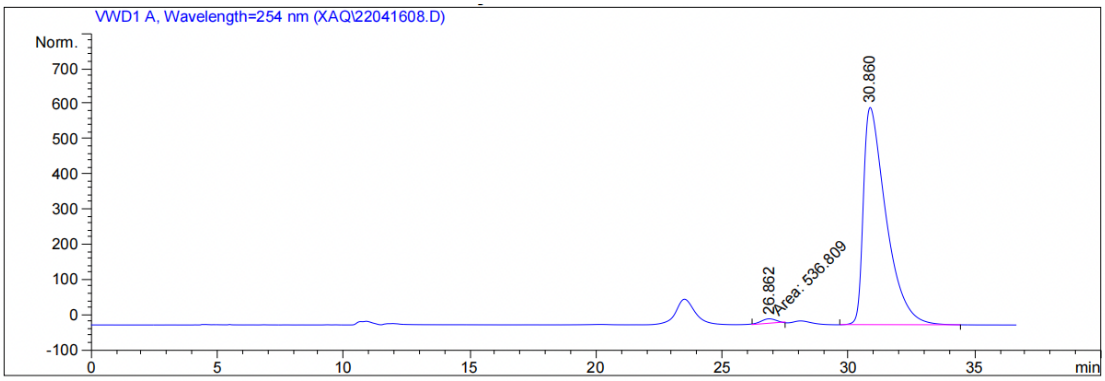

Peak #	RetTime [min]	Type	Width [min]	Area mAU *s	Height [mAU]	Area %
1	26.862	MM	0.6872	536.80902	13.01984	1.3684
2	30.860	BB	0.9290	3.86922e4	618.72870	98.6316

Compound 3p

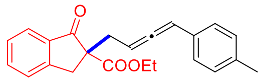

3p

Prepared according to the procedure within 72 h as light yellow liquid (68.5 mg, 99% yield, dr = 13 : 1). $[\alpha]_D^{13}$ = 64.270 (c 0.45, CH_2Cl_2); ^{1}H NMR (400 MHz, Chloroform-*d*) δ 7.77 (d, J = 7.6 Hz, 1H), 7.65 – 7.52 (m, 1H), 7.48 – 7.33 (m, 3H), 7.09 (s, 3H), 6.05 (dt, J = 6.4, 2.7 Hz, 1H), 5.45 (q, J = 6.8 Hz, 1H), 4.10 (q, J = 7.1 Hz, 2H), 3.74 (d, J = 17.4 Hz, 1H), 3.24 (d, J = 17.4 Hz, 1H), 3.00 (ddd, J = 14.6, 7.0, 2.8 Hz, 1H), 2.66 (ddd, J = 14.5, 7.2, 2.6 Hz, 1H), 2.32 (s, 3H), 1.17 (t, J = 7.1 Hz, 3H); ^{13}C NMR (101 MHz, Chloroform-*d*) δ 206.3, 201.9, 170.4, 153.3, 136.8, 135.4, 135.2, 131.0, 129.3, 127.7, 126.7, 126.5, 124.8, 95.3, 89.9, 61.8, 60.4, 36.5, 34.3, 21.2, 14.0. HRMS (ESI) m/z Calcd. for $C_{23}H_{22}NaO_3$ ($[M+Na]^+$) 369.1461, Found 369.1455. Enantiomeric excess was determined to be 93% (determined by HPLC using chiral AD-OD-H column, hexane/2-propanol = 50/1, λ = 254 nm, 30 ºC, 0.6 mL/min, t_{major} = 59.1 min, t_{minor} = 54.5 min).

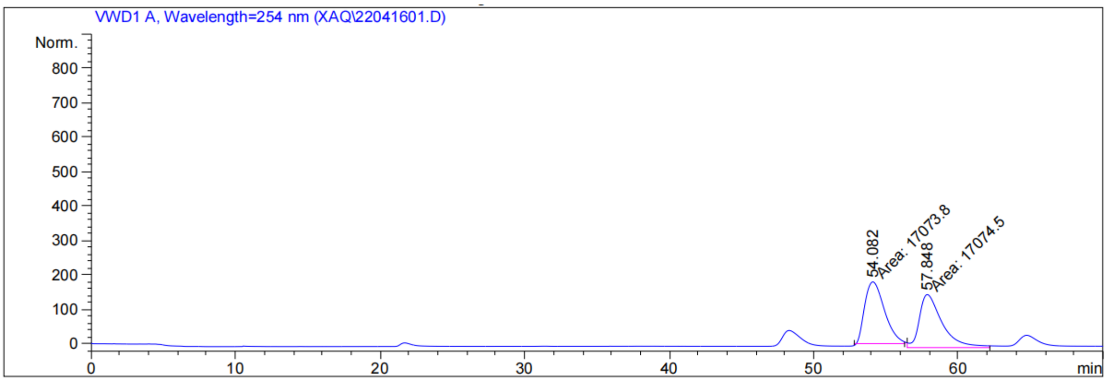

Peak #	RetTime [min]	Type	Width [min]	Area mAU *s	Height [mAU]	Area %
1	54.082	MM	1.5859	1.70738e4	179.42807	49.9990
2	57.848	MM	1.8436	1.70745e4	154.35524	50.0010

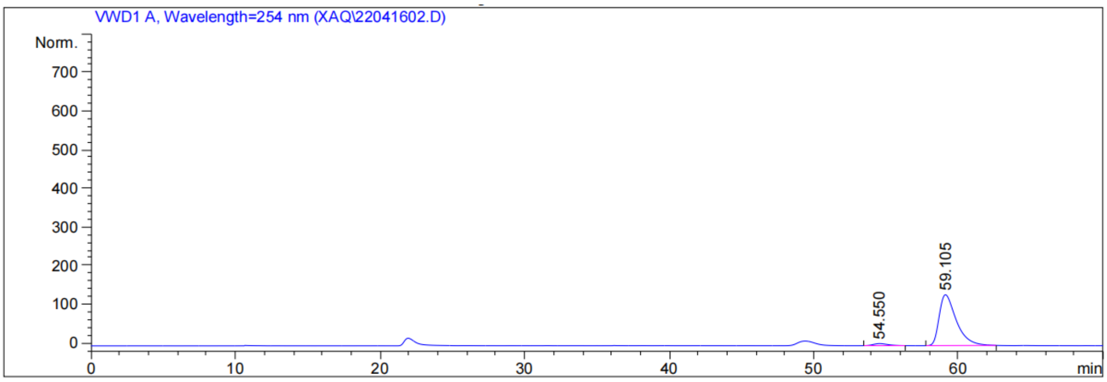

Peak #	RetTime [min]	Type	Width [min]	Area mAU *s	Height [mAU]	Area %
1	54.550	BB	0.9812	367.00092	5.27097	3.2719
2	59.105	BB	1.2287	1.08499e4	131.55251	96.7281

Compound 3q

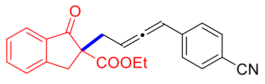

3q

Prepared according to the procedure within 72 h as light yellow liquid (57.9 mg, 81% yield, dr = 12 : 1). $[\alpha]_D^{15}$ = 88.378 (c 0.37, CH_2Cl_2); ^{1}H NMR (400 MHz, Chloroform-d) δ 7.69 (d, J = 7.7 Hz, 1H), 7.51 (dd, J = 7.5, 1.2 Hz, 1H), 7.46 (d, J = 8.1 Hz, 2H), 7.39 – 7.28 (m, 2H), 7.23 – 7.14 (m, 2H), 6.01 (dt, J = 6.0, 2.8 Hz, 1H), 5.50 (q, J = 6.9 Hz, 1H), 4.01 (q, J = 7.1 Hz, 2H), 3.65 (d, J = 17.3 Hz, 1H), 3.11 (d, J = 17.3 Hz, 1H), 2.90 (ddd, J = 14.8, 7.0, 2.9 Hz, 1H), 2.69 (ddd, J = 14.8, 7.3, 2.7 Hz, 1H), 1.08 (t, J = 7.1 Hz, 3H); ^{13}C NMR (101 MHz, Chloroform-d) δ 207.6, 201.6, 170.3, 153.0, 139.3, 135.5, 135.3, 132.3, 127.9, 127.2, 126.4, 124.8, 119.0, 110.2, 94.8, 91.0, 61.9, 60.0, 36.5, 33.6, 14.0. HRMS (ESI) m/z Calcd. for $C_{23}H_{19}NNaO_3$ ($[M+Na]^+$) 380.1257, Found 380.1248. Enantiomeric excess was determined to be 97% (determined by HPLC using chiral OD-H column, hexane/2-propanol = 95/5, λ = 254 nm, 30 °C, 0.8 mL/min, t_{major} = 41.0 min, t_{minor} = 36.8 min).

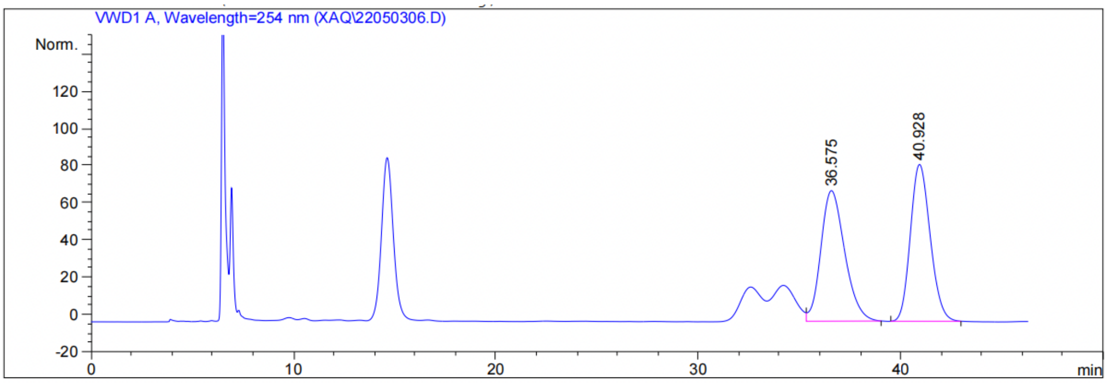

Peak #	RetTime [min]	Type	Width [min]	Area mAU *s	Height [mAU]	Area %
1	36.575	VB	1.2594	5768.87939	70.14637	50.0797
2	40.928	BB	1.0542	5750.52832	84.23426	49.9203

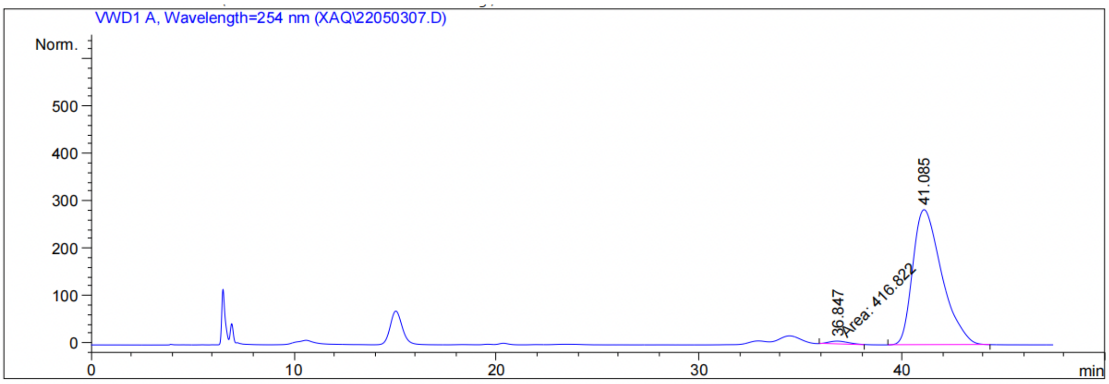

Peak #	RetTime [min]	Type	Width [min]	Area mAU *s	Height [mAU]	Area %
1	36.847	MM	1.1566	416.82187	6.00620	1.4457
2	41.085	BB	1.5275	2.84160e4	285.28763	98.5543

Compound 3r

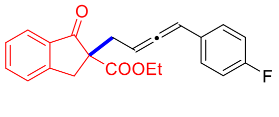

3r

Prepared according to the procedure within 48 h as light yellow liquid (60.9 mg, 87% yield, dr = 10 : 1). $[\alpha]_D^{17}$ = 79.750 (*c* 0.40, CH_2Cl_2); ^{1}H NMR (400 MHz, Chloroform-*d*) δ 7.76 (d, *J* = 7.7 Hz, 1H), 7.60 – 7.56 (m, 1H), 7.44 – 7.35 (m, 2H), 7.17 – 7.10 (m, 2H), 7.01 – 6.93 (m, 2H), 6.04 (dt, *J* = 6.3, 2.8 Hz, 1H), 5.47 (q, *J* = 6.8 Hz, 1H), 4.09 (q, *J* = 7.1 Hz, 2H), 3.73 (d, *J* = 17.4 Hz, 1H), 3.21 (d, *J* = 17.3 Hz, 1H), 2.98 (ddd, *J* = 14.8, 6.9, 2.8 Hz, 1H), 2.70 (ddd, *J* = 14.7, 7.1, 2.7 Hz, 1H), 1.16 (t, *J* = 7.1 Hz, 3H); ^{13}C NMR (101 MHz, Chloroform-*d*) δ 206.2, 201.8, 170.4, 162.0 (d, *J* = 247.5 Hz), 153.2, 135.3 (d, *J* = 6.1 Hz), 130.0, 128.3, 128.2, 127.8, 126.4, 124.7, 115.5 (d, *J* = 21.2 Hz), 94.6, 90.3, 61.8, 60.2, 36.5, 34.1, 14.0. ^{19}F NMR (376 MHz, Chloroform-*d*) δ -102.3 (q, *J* = 6.5 Hz); HRMS (ESI) m/z Calcd. for $C_{22}H_{19}FNaO_3$ ($[M+Na]^+$) 373.1210, Found 373.1203. Enantiomeric excess was determined to be 97% (determined by HPLC using chiral AD-OD-H column, hexane/2-propanol = 50/1, λ = 254 nm, 30 ºC, 0.6 mL/min, t_{major} = 64.5 min, t_{minor} = 61.0 min).

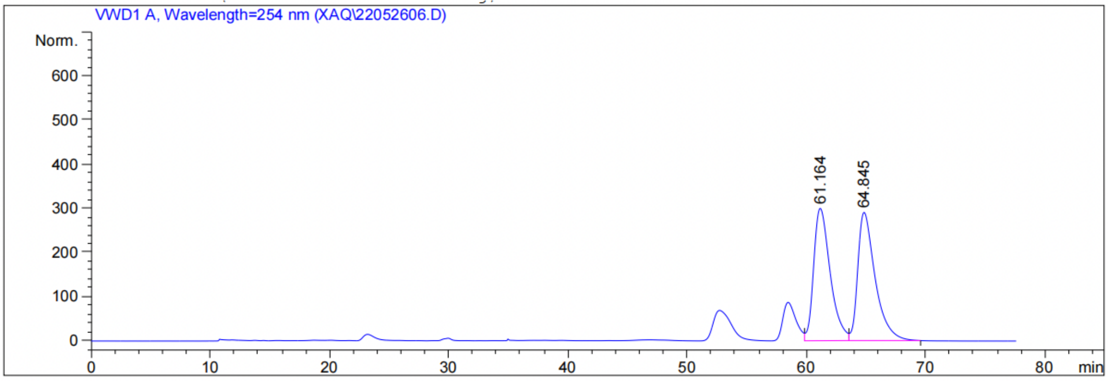

Peak #	RetTime [min]	Type	Width [min]	Area mAU *s	Height [mAU]	Area %
1	61.164	VV	1.4440	2.89756e4	300.06607	49.5049
2	64.845	VB	1.5193	2.95552e4	290.77267	50.4951

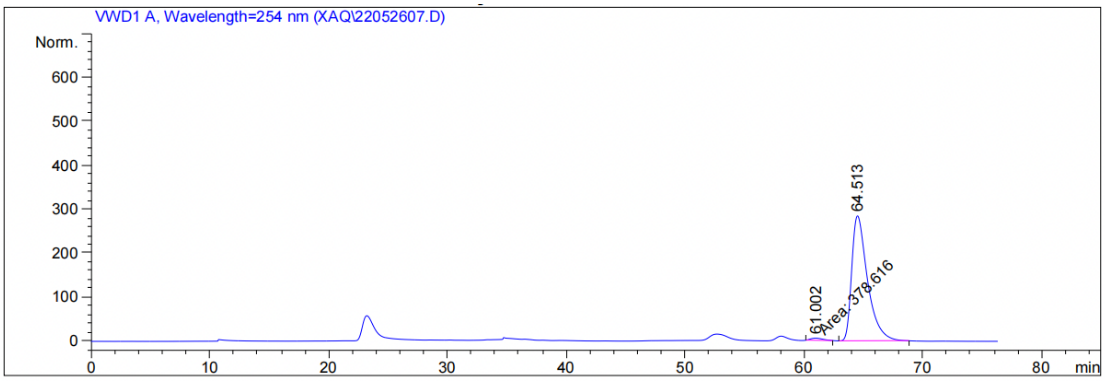

Peak #	RetTime [min]	Type	Width [min]	Area mAU *s	Height [mAU]	Area %
1	61.002	MM	1.2162	378.61636	5.18860	1.4198
2	64.513	VB	1.3747	2.62890e4	284.58508	98.5802

Compound 3s

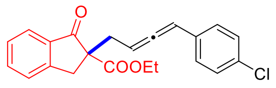

3s

Prepared according to the procedure within 48 h as light yellow liquid (61.5 mg, 84% yield, dr = 11 : 1). $[\alpha]_D^{17}$ = 72.922 (c 0.42, CH_2Cl_2); ^{1}H NMR (400 MHz, Chloroform-d) δ 7.76 (d, J = 7.7 Hz, 1H), 7.60-7.56 (m, 1H), 7.46 – 7.34 (m, 2H), 7.30 – 7.19 (m, 2H), 7.15 – 7.06 (m, 2H), 6.03 (dt, J = 6.2, 2.8 Hz, 1H), 5.49 (q, J = 6.9 Hz, 1H), 4.09 (q, J = 7.1 Hz, 2H), 3.73 (d, J = 17.4 Hz, 1H), 3.20 (d, J = 17.3 Hz, 1H), 2.98 (ddd, J = 14.7, 6.9, 2.9 Hz, 1H), 2.70 (ddd, J = 14.7, 7.2, 2.7 Hz, 1H), 1.16 (t, J = 7.1 Hz, 3H); ^{13}C NMR (101 MHz, Chloroform-d) δ 206.5, 201.8, 170.4, 153.1, 135.4, 135.3, 132.6, 132.6, 128.7, 128.0, 127.8, 126.4, 124.7, 94.7, 90.5, 61.8, 60.2, 36.5, 34.0, 14.0. HRMS (ESI) m/z Calcd. for $C_{22}H_{19}ClNaO_3$ ($[M+Na]^+$) 389.0915, Found 389.0907. Enantiomeric excess was determined to be 91% (determined by HPLC using chiral IC-IB-H column, hexane/2-propanol = 9/1, λ = 254 nm, 30 °C, 0.6 mL/min, t_{major} = 41.9 min, t_{minor} = 37.0 min).

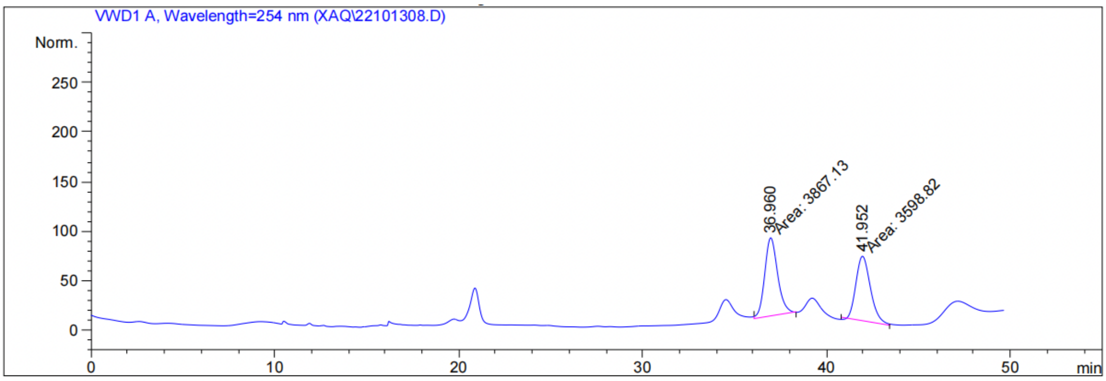

Peak #	RetTime [min]	Type	Width [min]	Area mAU *s	Height [mAU]	Area %
1	36.960	MM	0.8155	3867.12744	79.03625	51.7969
2	41.952	MM	0.9184	3598.81641	65.31152	48.2031

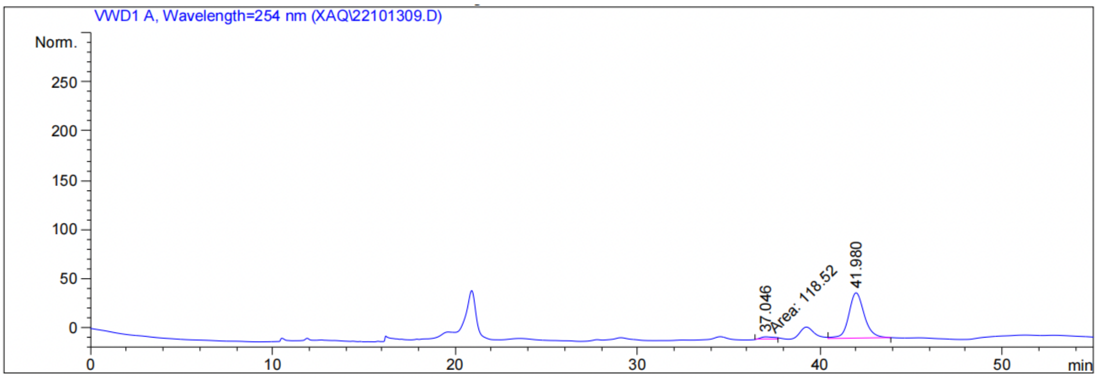

Peak #	RetTime [min]	Type	Width [min]	Area mAU *s	Height [mAU]	Area %
1	37.046	MM	0.8529	118.51983	2.31596	4.1205
2	41.980	VB	0.9064	2757.85767	46.27449	95.8795

Compound 3t

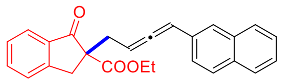

3t

Prepared according to the procedure within 72 h as light yellow liquid (55.8 mg, 73% yield, dr = 9 : 1). $[\alpha]_D^{16}$ = 94.309 (c 0.25, CH_2Cl_2); ^{1}H NMR (400 MHz, Chloroform-d) δ 7.84 – 7.70 (m, 4H), 7.54 (d, J = 7.9 Hz, 2H), 7.44 (d, J = 6.0 Hz, 2H), 7.40 – 7.35 (m, 3H), 6.24 (dd, J = 6.7, 3.1 Hz, 1H), 5.55 (d, J = 6.9 Hz, 1H), 4.10 (q, J = 7.4 Hz, 2H), 3.76 (d, J = 17.4 Hz, 1H), 3.27 (d, J = 17.3 Hz, 1H), 3.04 (dd, J = 15.3, 6.7 Hz, 1H), 2.73 (dd, J = 15.3, 7.6 Hz, 1H), 1.16 (t, J = 7.1 Hz, 3H); ^{13}C NMR (101 MHz, Chloroform-d) δ 207.0, 201.8, 170.4, 153.2, 135.4, 135.3, 133.7, 132.7, 131.6, 128.5, 128.2, 127.7, 126.4, 126.2, 125.7, 125.7, 125.5, 124.7, 124.7, 95.9, 90.3, 61.8, 60.4, 36.5, 34.2, 14.0. HRMS (ESI) m/z Calcd. for $C_{26}H_{22}NaO_3$ ($[M+Na]^+$) 405.1461, Found 405.1455. Enantiomeric excess was determined to be 97% (determined by HPLC using chiral AD-H column, hexane/2-propanol = 50/1, λ = 254 nm, 30 ºC, 0.8 mL/min, t_{major} = 49.1 min, t_{minor} = 45.2 min).

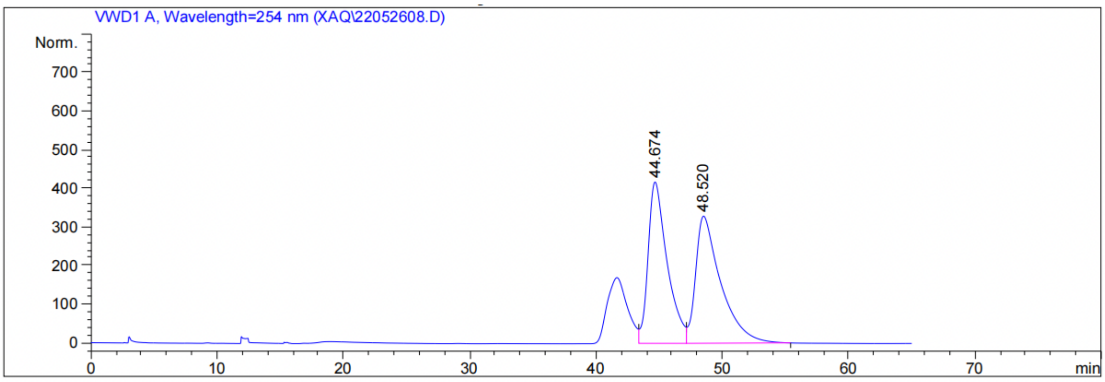

Peak #	RetTime [min]	Type	Width [min]	Area mAU *s	Height [mAU]	Area %
1	44.674	VV	1.5141	4.38809e4	418.14081	49.0461
2	48.520	VB	1.9516	4.55877e4	329.27954	50.9539

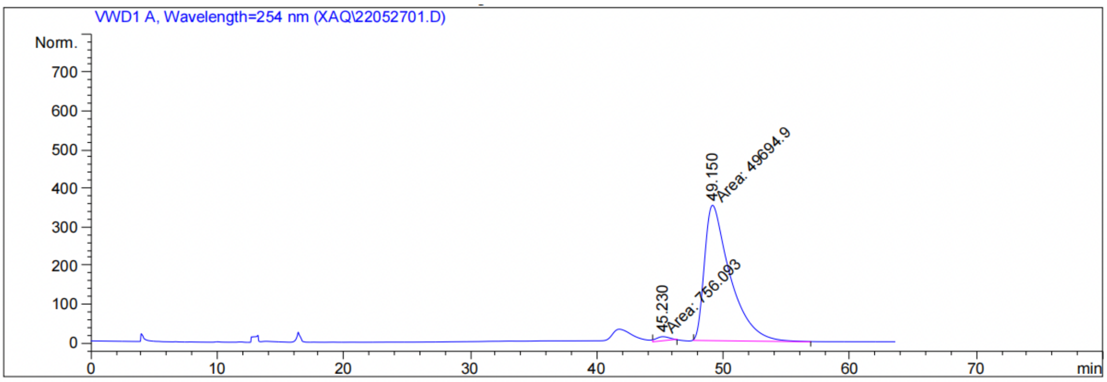

Peak #	RetTime [min]	Type	Width [min]	Area mAU *s	Height [mAU]	Area %
1	45.230	MM	1.1907	756.09338	10.58366	1.4987
2	49.150	MM	2.3613	4.96949e4	350.76199	98.5013

Compound 3u

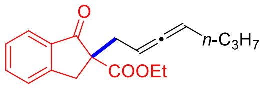

3u

Prepared according to the procedure within 84 h as light yellow liquid (50.7 mg, 85% yield, dr = 2 : 1). $[\alpha]_D^{16}$ = 49.275 (c 0.14, CH_2Cl_2); ^{1}H NMR (400 MHz, Chloroform-*d*) δ 7.76 (d, *J* = 7.7 Hz, 1H), 7.65 – 7.57 (m, 1H), 7.48 (d, *J* = 7.7 Hz, 1H), 7.40-7.37 (m, 1H), 5.14 – 4.83 (m, 2H), 4.24 – 4.07 (m, 2H), 3.68 (dd, *J* = 17.3, 2.6 Hz, 1H), 3.23 (d, *J* = 17.3 Hz, 1H), 2.83 (dtd, *J* = 15.1, 7.6, 2.5 Hz, 1H), 2.54 (dddd, *J* = 14.5, 7.4, 5.2, 2.6 Hz, 1H), 1.87 (qd, *J* = 7.1, 3.6 Hz, 2H), 1.36 (qd, *J* = 7.4, 2.1 Hz, 2H), 1.21 (t, *J* = 7.1 Hz, 3H), 0.88 (t, *J* = 7.4, 3H); ^{13}C NMR (101 MHz, Chloroform-*d*) δ 205.7, 202.1, 170.6, 153.4, 135.4, 135.3, 127.6, 126.4, 124.7, 91.4, 85.6, 61.6, 60.6, 36.2, 34.6, 30.9, 22.3, 14.1, 13.6. HRMS (ESI) m/z Calcd. for $C_{19}H_{22}NaO_3$ ($[M+Na]^+$) 321.1461, Found 321.1453. Enantiomeric excess was determined to be 79%/71% (determined by HPLC using chiral AS-AD-H column, hexane/2-propanol = 50/1, λ = 254 nm, 30 ºC, 0.6 mL/min, t_{major} = 30.5/26.7 min, t_{minor} = 25.6/28.2 min).

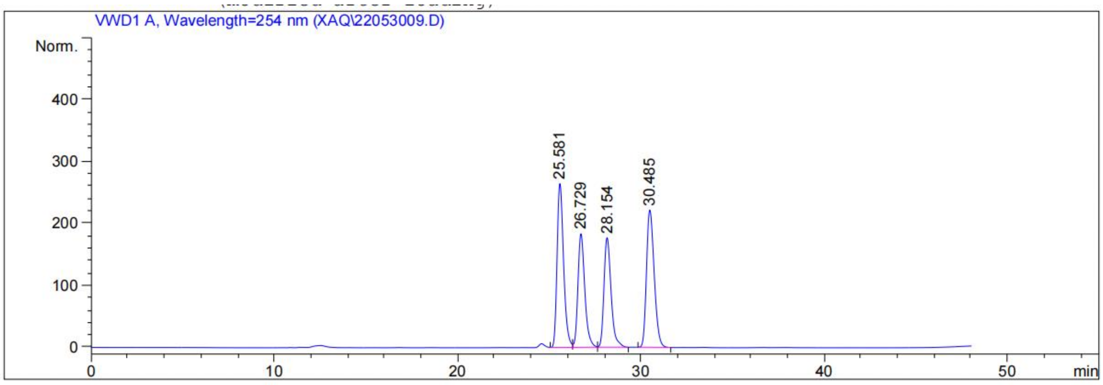

Peak #	RetTime [min]	Type	Width [min]	Area mAU *s	Height [mAU]	Area %
1	25.581	VV	0.3795	6528.91553	264.65936	28.8985
2	26.729	VV	0.3995	4793.75439	183.45493	21.2183
3	28.154	VP	0.4162	4788.67285	176.94559	21.1958
4	30.485	BB	0.4546	6481.22900	221.69411	28.6874

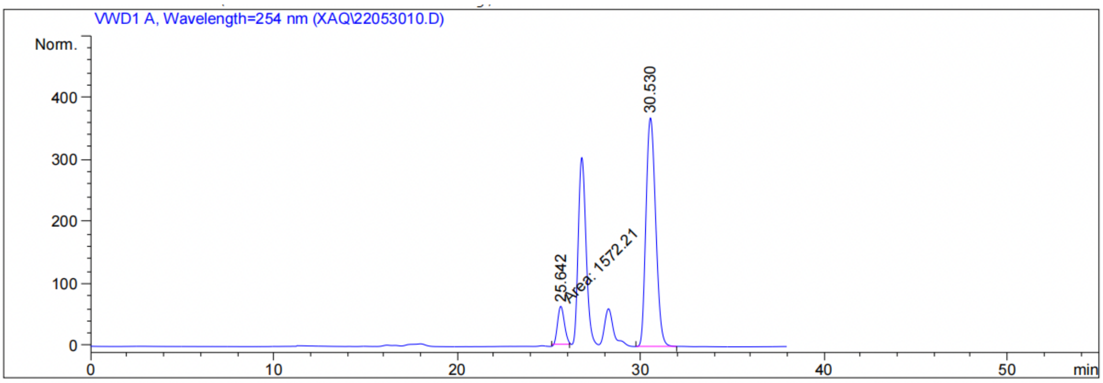

Peak #	RetTime [min]	Type	Width [min]	Area mAU *s	Height [mAU]	Area %
1	25.642	MM	0.4281	1572.21033	61.21218	10.3759
2	30.530	PB	0.5855	1.35803e4	368.73114	89.6241

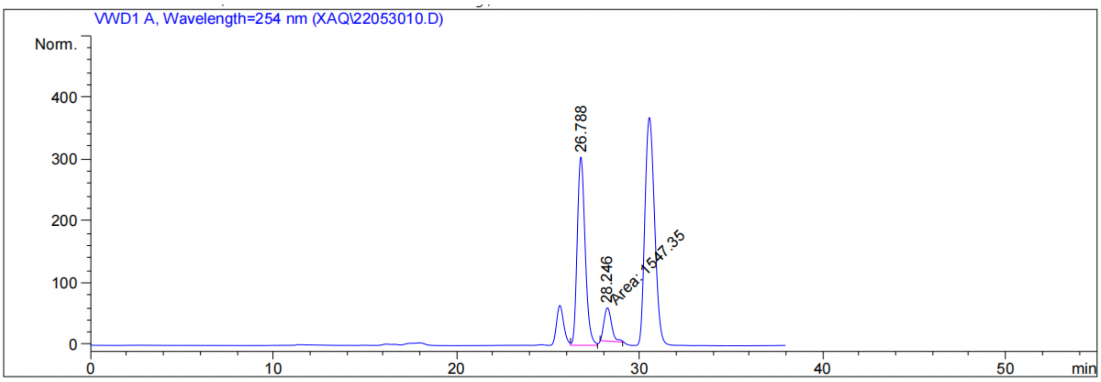

Peak #	RetTime [min]	Type	Width [min]	Area mAU *s	Height [mAU]	Area %
1	26.788	VV	0.4686	9160.58008	304.76843	85.5495
2	28.246	MM	0.4774	1547.35376	54.02264	14.4505

Compound 3v

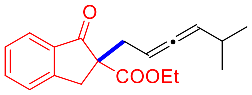

3v

Prepared according to the procedure within 84 h as light yellow liquid (49.5 mg, 83% yield, dr = 2 : 1). $[\alpha]_D^{16}$ = 67.677 (*c* 0.20, CH_2Cl_2); ^{1}H NMR (600 MHz, Chloroform-*d*) δ 7.76 (dd, *J* = 7.7, 3.9 Hz, 1H), 7.65 – 7.59 (m, 1H), 7.48 (dd, *J* = 7.8, 2.9 Hz, 1H), 7.44 – 7.35 (m, 1H), 5.15 – 4.89 (m, 2H), 4.32 – 4.08 (m, 2H), 3.68 (d, *J* = 17.2 Hz, 1H), 3.24 (dd, *J* = 17.3, 3.7 Hz, 1H), 2.94 – 2.77 (m, 1H), 2.55 (ddt, *J* = 14.2, 7.6, 2.0 Hz, 1H), 2.20 (dp, *J* = 9.7, 3.1 Hz, 1H), 1.20 (t, *J* = 7.1 Hz, 3H), 0.94 (ddd, *J* = 6.5, 4.6, 1.6 Hz, 6H); ^{13}C NMR (151 MHz, Chloroform-*d*) δ 204.2, 202.2, 170.7, 153.4, 135.3, 129.0, 127.7, 126.4, 124.8, 98.9, 86.8, 61.7, 60.6, 36.2, 34.8, 27.9, 22.4, 14.1. HRMS (ESI) m/z Calcd. for $C_{19}H_{22}NaO_3$ ($[M+Na]^+$) 321.1461, Found 321.1453. Enantiomeric excess was determined to be 77%/55% (determined by HPLC using chiral AS-AD-H column, hexane/2-propanol = 50/1, λ = 254 nm, 30 °C, 0.6 mL/min, t_{major} = 27.5/24.7 min, t_{minor} = 23.8/26.2 min).

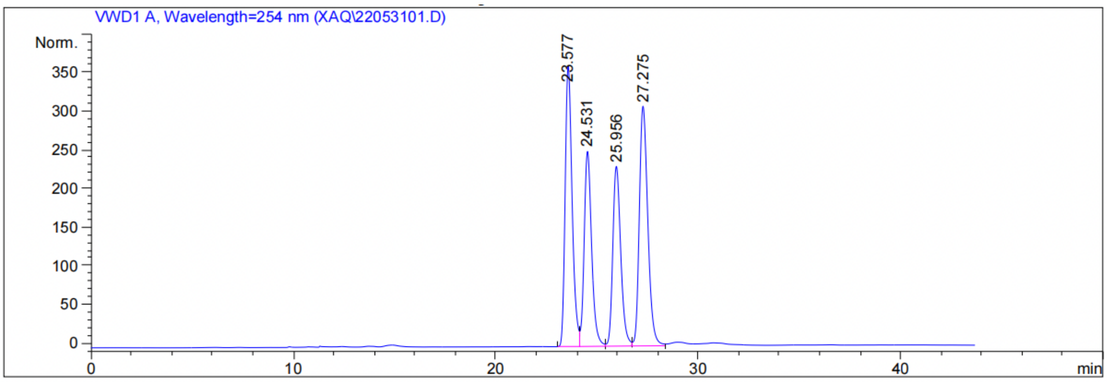

Peak #	RetTime [min]	Type	Width [min]	Area mAU *s	Height [mAU]	Area %
1	23.577	BV	0.3693	8710.25684	362.31714	28.4345
2	24.531	VV	0.3930	6481.42627	252.23541	21.1585
3	25.956	VV	0.4235	6381.93896	232.61856	20.8337
4	27.275	VB	0.4454	9059.07324	310.31287	29.5732

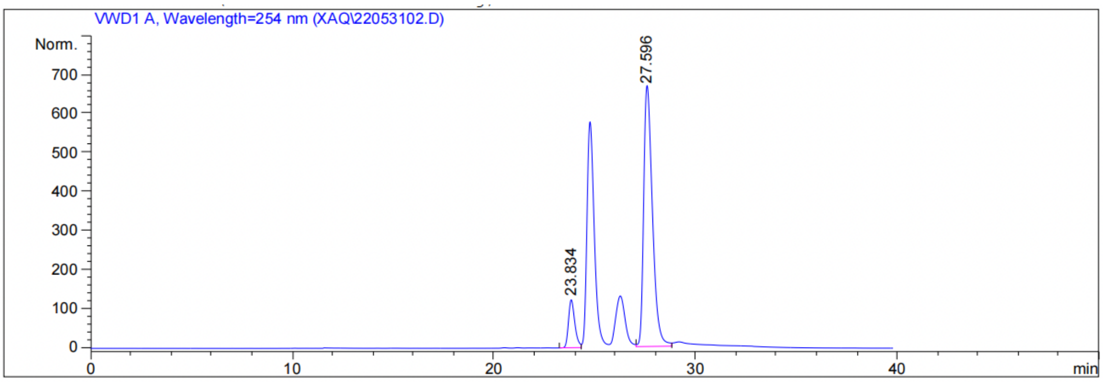

Peak #	RetTime [min]	Type	Width [min]	Area mAU *s	Height [mAU]	Area %
1	23.834	PV	0.3339	2696.69287	123.23420	11.7322
2	27.596	VB	0.4549	2.02887e4	670.48602	88.2678

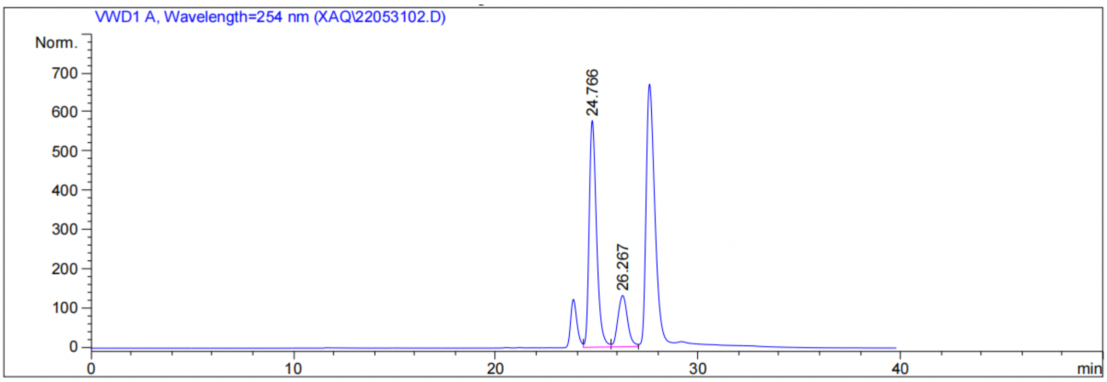

Peak #	RetTime [min]	Type	Width [min]	Area mAU *s	Height [mAU]	Area %
1	24.766	VV	0.3917	1.47558e4	579.51996	77.1425
2	26.267	VV	0.5180	4372.16357	131.32794	22.8575

Compound 3w

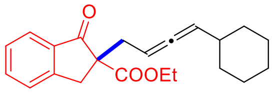

3w

Prepared according to the procedure within 84 h as light yellow liquid (52.8 mg, 78% yield, dr = 2 : 1). $[\alpha]_D^{16}$ = 43.017 (*c* 0.18, CH_2Cl_2); ^{1}H NMR (400 MHz, Chloroform-*d*) δ 7.80 – 7.72 (m, 1H), 7.64-7.6 (m, 1H), 7.48 (d, *J* = 7.7 Hz, 1H), 7.44 – 7.34 (m, 1H), 4.99 (dtt, *J* = 23.8, 6.8, 3.1 Hz, 2H), 4.15 (qd, *J* = 7.1, 1.5 Hz, 2H), 3.68 (d, *J* = 17.4 Hz, 1H), 3.24 (d, *J* = 17.3 Hz, 1H), 2.83 (dddd, *J* = 14.1, 6.9, 4.1, 2.6 Hz, 1H), 2.56 (dddd, *J* = 14.9, 7.9, 5.5, 2.7 Hz, 1H), 1.86 (dddd, *J* = 11.2, 8.7, 5.9, 3.1 Hz, 1H), 1.66 (tt, *J* = 18.8, 7.4 Hz, 5H), 1.39 – 1.10 (m, 6H), 1.00 (tdd, *J* = 13.6, 7.7, 3.7 Hz, 2H); ^{13}C NMR (101 MHz, Chloroform-*d*) δ 204.5, 202.2, 170.7, 153.4, 135.4, 129.0, 128.4,

127.7, 126.4, 124.8, 97.5, 86.4, 61.7, 60.6, 37.2, 36.2, 34.8, 32.9, 26.1, 14.1. HRMS (ESI) m/z Calcd. for $C_{22}H_{26}NaO_3$ ($[M+Na]^+$) 361.1774, Found 361.1765. Enantiomeric excess was determined to be 73%/55% (determined by HPLC using chiral AS-AD-H column, hexane/2-propanol = 50/1, λ = 254 nm, 30 °C, 0.6 mL/min, t_{major} = 29.9/26.9 min, t_{minor} = 26.3/27.9 min).

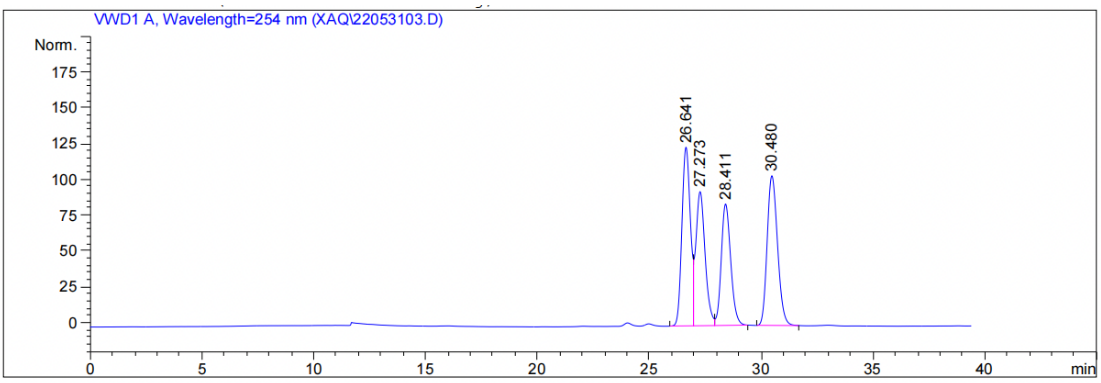

Peak #	RetTime [min]	Type	Width [min]	Area mAU *s	Height [mAU]	Area %
1	26.641	BV	0.4097	3308.96313	124.82241	27.8361
2	27.273	VV	0.4328	2704.63257	93.71778	22.7523
3	28.411	VB	0.4487	2500.18848	84.82932	21.0324
4	30.480	BB	0.5061	3373.52319	104.54383	28.3792

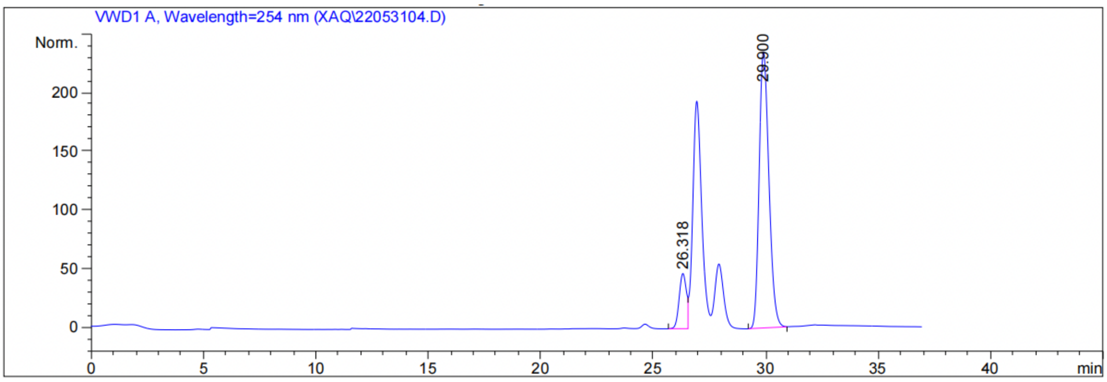

Peak #	RetTime [min]	Type	Width [min]	Area mAU *s	Height [mAU]	Area %
1	26.318	BV	0.3480	1064.20813	47.11448	13.0148
2	29.900	PB	0.4691	7112.71826	235.29587	86.9852

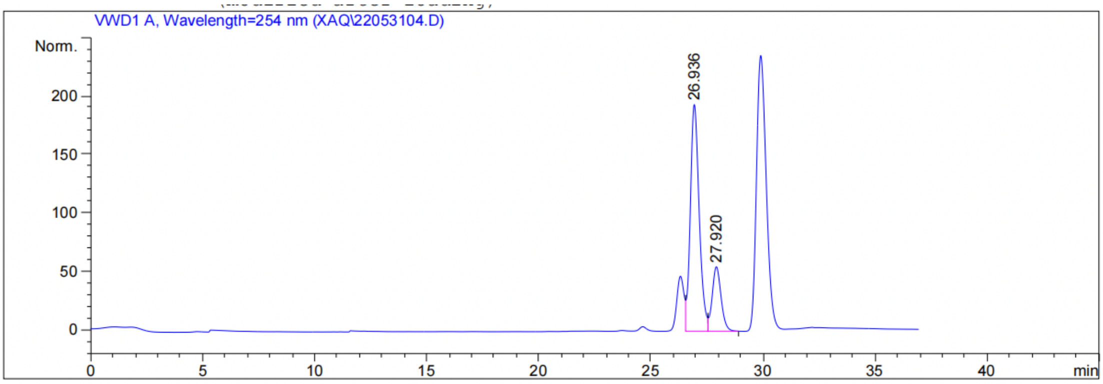

Peak #	RetTime [min]	Type	Width [min]	Area mAU *s	Height [mAU]	Area %
1	26.936	VV	0.4179	5352.04004	194.01195	77.7711
2	27.920	VB	0.4221	1529.74561	55.23497	22.2289

Compound 3x

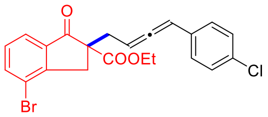

3x

Prepared according to the procedure within 48 h as light yellow liquid (87.9 mg, 99% yield, dr = 10 : 1). $[\alpha]_D^{16}$ = 101.589 (c 1.20, CH_2Cl_2); ^1H NMR (600 MHz, Chloroform-d) δ 7.72-7.69 (m, 2H), 7.26 – 7.21 (m, 3H), 7.07 (d, J = 8.3 Hz, 2H), 6.01 (dt, J = 6.0, 2.8 Hz, 1H), 5.50 (q, J = 6.8 Hz, 1H), 4.11 (qd, J = 7.1, 3.2 Hz, 2H), 3.62 (d, J = 17.8 Hz, 1H), 3.14 (d, J = 17.8 Hz, 1H), 2.98 (ddd, J = 14.9, 6.7, 2.9 Hz, 1H), 2.72 (ddd, J = 14.9, 7.1, 2.8 Hz, 1H), 1.18 (t, J = 7.1 Hz, 3H). ^{13}C NMR (101 MHz, Chloroform-d) δ 206.4, 201.2, 170.0, 152.7, 138.0, 137.3, 132.7, 132.4, 129.6, 128.7, 127.9, 123.4, 122.0, 95.1, 90.2, 62.0, 60.2, 37.6, 33.9, 14.0. HRMS (ESI) m/z Calcd. for $C_{22}H_{18}BrClNaO_3$ ($[M+Na]^+$) 467.0020, Found 467.0022. Enantiomeric excess was determined to be 96% (determined by HPLC using chiral AD-OJ-H column, hexane/2-propanol = 50/1, λ = 254 nm, 30 °C, 0.6 mL/min, t_{major} = 92.3 min, t_{minor} = 83.9 min).

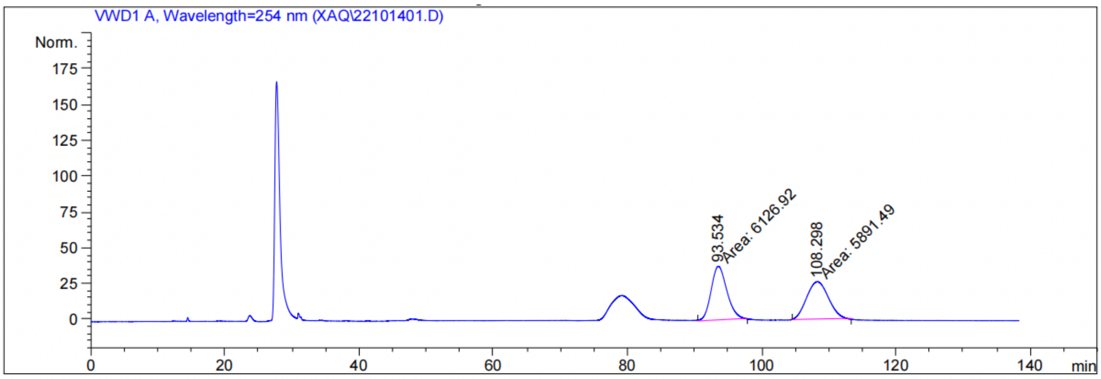

Peak #	RetTime [min]	Type	Width [min]	Area mAU *s	Height [mAU]	Area %
1	93.534	MM	2.7255	6126.92480	37.46648	50.9795
2	108.298	MM	3.7582	5891.48975	26.12732	49.0205

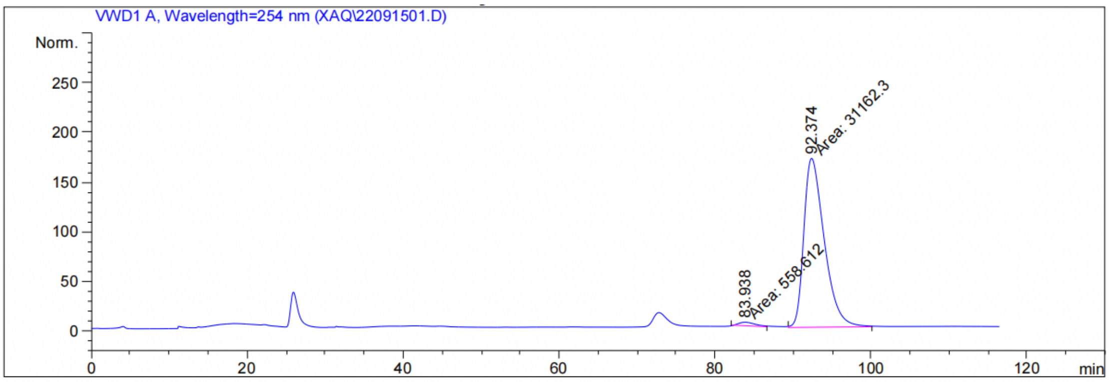

Peak #	RetTime [min]	Type	Width [min]	Area mAU *s	Height [mAU]	Area %
1	83.938	MM	2.5199	558.61176	3.69472	1.7610
2	92.374	MM	3.0470	3.11623e4	170.45348	98.2390

Gram scale synthesis of the product 3j

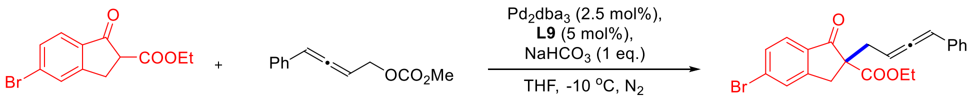

1j, 2.50 mmol **2a**, 3.0 mmol **3j**, 1.01 g
dr = 12:1, 98% yield, 97% ee

L9 (76mg, 5 mol%) and Pd_2dba_3 (57mg, 2.5 mol%) were stirred in THF (50 mL) in a Schlenk flask under a nitrogen atmosphere at room temperature for 10 min. To this Schlenk tube were added **1j** (705 mg, 2.50 mmol, 1.0 equiv), $NaHCO_3$ (210 mg, 2.50 mmol, 1.0 equiv) and **2a** (612 mg, 3.0 mmol, 1.2 equiv), then the reaction mixture was stirred at -10 ºC for 48 h. When compound **1j** was consumed as checked by TLC, the reaction was stopped and purified by column chromatography on silica gel directly to give the product **3j** 1.01 g as light yellow liquid (dr = 12:1, 98% yield, 97% ee).

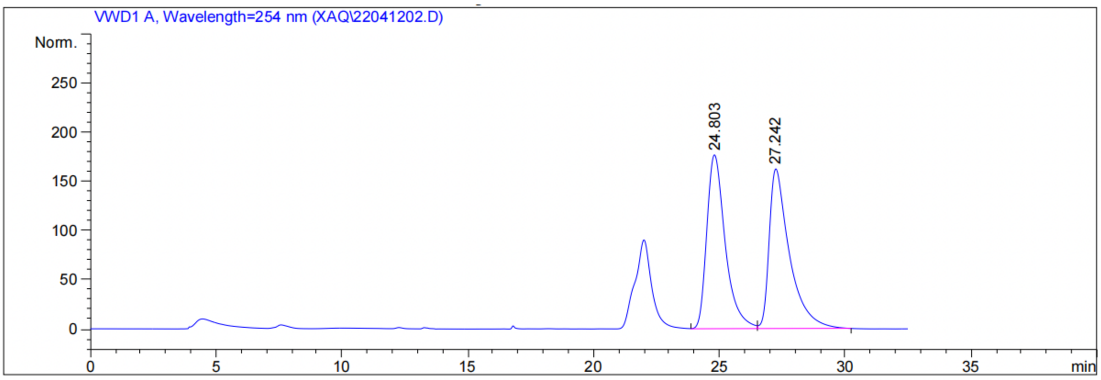

Peak #	RetTime [min]	Type	Width [min]	Area mAU *s	Height [mAU]	Area %
1	24.803	BV	0.7751	9018.91211	176.54369	49.7866
2	27.242	VB	0.8145	9096.20996	162.18997	50.2134

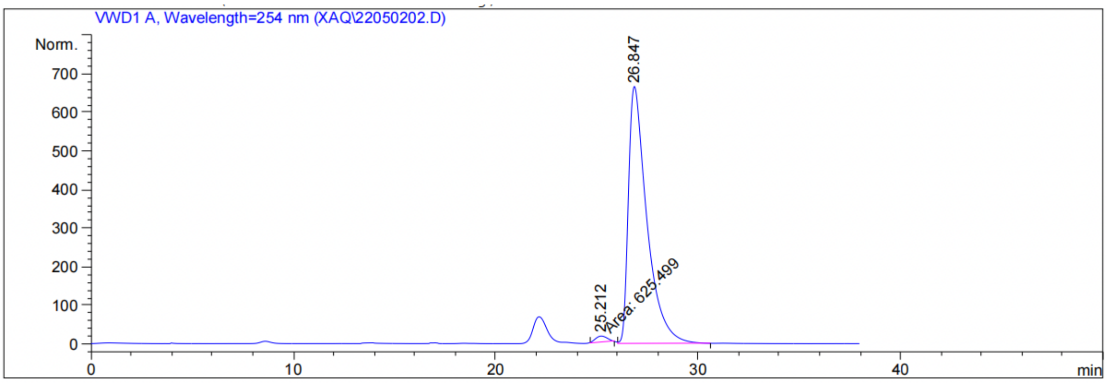

Peak #	RetTime [min]	Type	Width [min]	Area mAU *s	Height [mAU]	Area %
1	25.212	MM	0.6702	625.49915	15.55571	1.4623
2	26.847	VB	0.9345	4.21510e4	665.04877	98.5377

Synthesis of the product 4a

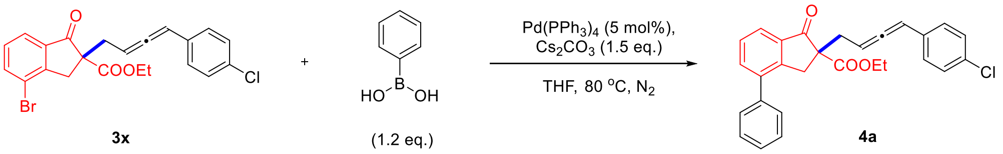

A Schlenk flask under a nitrogen atmosphere was charged with compound **3x** (222 mg, 0.50 mmol, 1.0 eq), phenylboronic acid (73.2 mg, 0.60 mmol, 1.2 eq), Cs_2CO_3 (244 mg, 0.75 mmol, 1.5 eq) and $Pd(PPh_3)_4$ (28.9 mg, 25.0 µmol, 5.0 mol%). THF (5 mL) added and the mixture was heated to 80 °C for 18 h, when compound **3x** was consumed as checked by TLC, the mixture was cooled to rt and diluted with Et_2O (15 mL). The mixture was washed with water (15 mL). The aq. layer was extracted with Et_2O (2 x 25 mL) and the combined org. layers were dried, filtered and concentrated. The crude product was purified by column chromatography (petroleum ether/ethyl acetate = 20:1) yielding the title compound **4a** as a slightly yellow oil.

Compound 4a

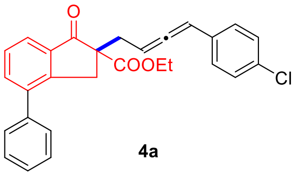

4a

Prepared according to the procedure within 18 h as slightly yellow oil (154 mg, 70% yield, dr = 9 : 1). $[\alpha]_D^{16}$ = 23.014 (*c* 0.37, CH_2Cl_2); ^{1}H NMR (400 MHz, Chloroform-*d*) δ 7.79 (dd, *J* = 18.7, 7.5 Hz, 1H), 7.63 (dd, *J* = 16.9, 7.2 Hz, 1H), 7.55 – 7.42 (m, 5H), 7.38 (dd, *J* = 6.6, 3.1 Hz, 1H), 7.32 – 7.27 (m, 1H), 7.16 (dd, *J* = 8.6, 2.1 Hz, 2H), 7.07 – 7.03 (m, 1H), 6.10 – 5.91 (m, 1H), 5.61 – 5.48 (m, 1H), 4.30 – 4.12 (m, 2H), 3.81 (dd, *J* = 17.5, 11.5 Hz, 1H), 3.22 (dd, *J* = 17.5, 9.8 Hz, 1H), 3.01 (tdd, *J* = 11.7, 7.0, 3.0 Hz, 1H), 2.70 (dddd, *J* = 30.5, 14.6, 7.2, 2.7 Hz, 1H), 1.23 (t, *J* = 7.1 Hz, 3H). ^{13}C NMR (101 MHz, Chloroform-*d*) δ 206.5, 201.8, 170.4, 150.6, 140.2, 138.7, 135.6, 132.6, 128.8, 128.7, 128.4, 128.4, 128.4, 127.9, 127.8, 123.8, 115.3, 94.5, 90.5, 61.9, 60.5, 36.5, 34.2, 14.1. HRMS (ESI) m/z Calcd. for $C_{28}H_{23}ClNaO_3$ ($[M+Na]^+$) 465.1228, Found 465.1225. Enantiomeric excess was determined to be 92% (determined by HPLC using chiral IF-OD-H column, hexane/2-propanol = 95/5, λ = 254 nm, 30 °C, 0.6 mL/min, t_{major} = 41.3 min, t_{minor} = 44.3 min).

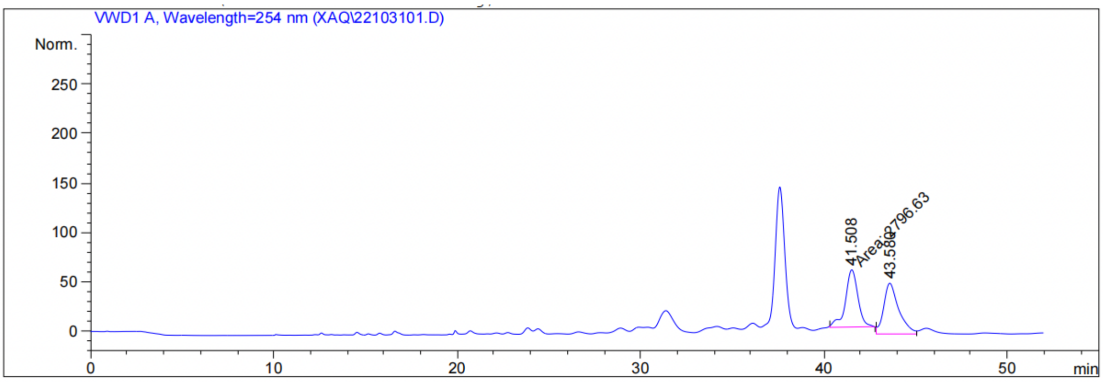

Peak #	RetTime [min]	Type	Width [min]	Area mAU *s	Height [mAU]	Area %
1	41.508	MM	0.8005	2796.62793	58.22775	48.6870
2	43.580	VV	0.8338	2947.47241	51.48830	51.3130

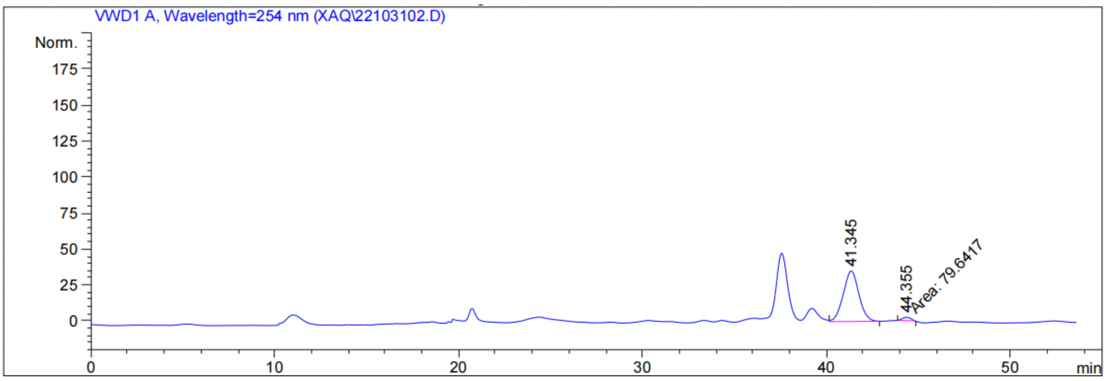

Peak #	RetTime [min]	Type	Width [min]	Area mAU *s	Height [mAU]	Area %
1	41.345	VB	0.8859	2082.29346	35.16706	96.3162
2	44.355	MM	0.5499	79.64166	2.41374	3.6838

4. NMR spectra for compounds

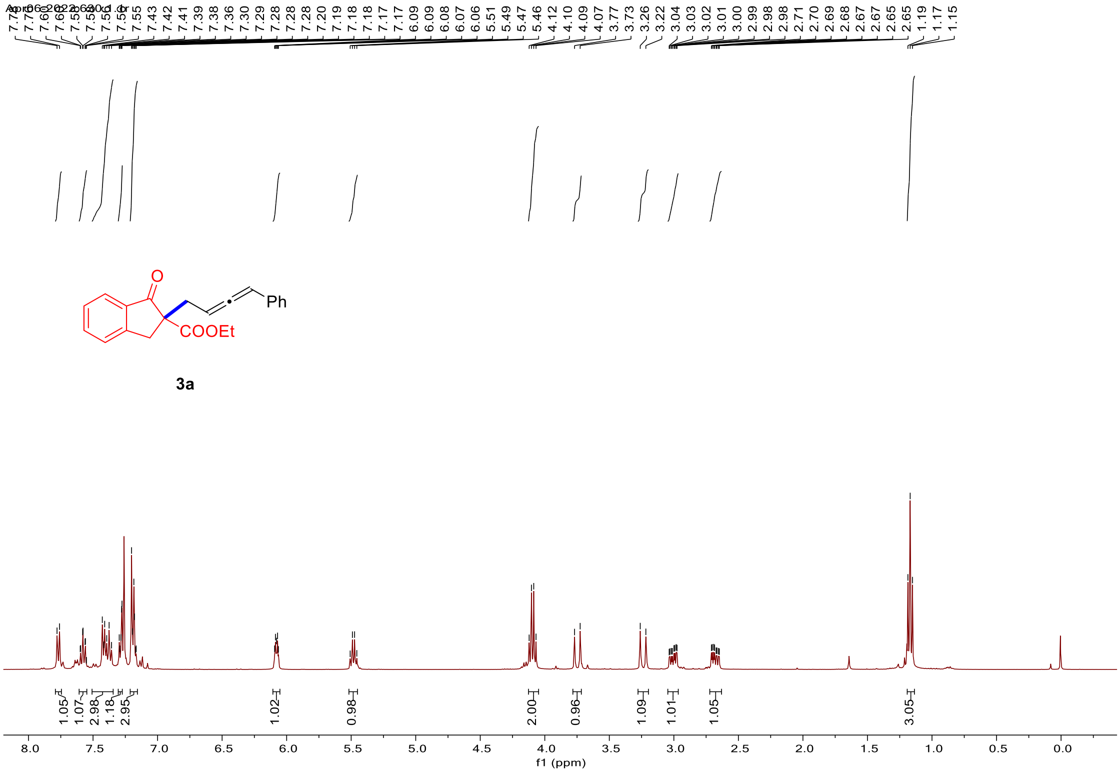

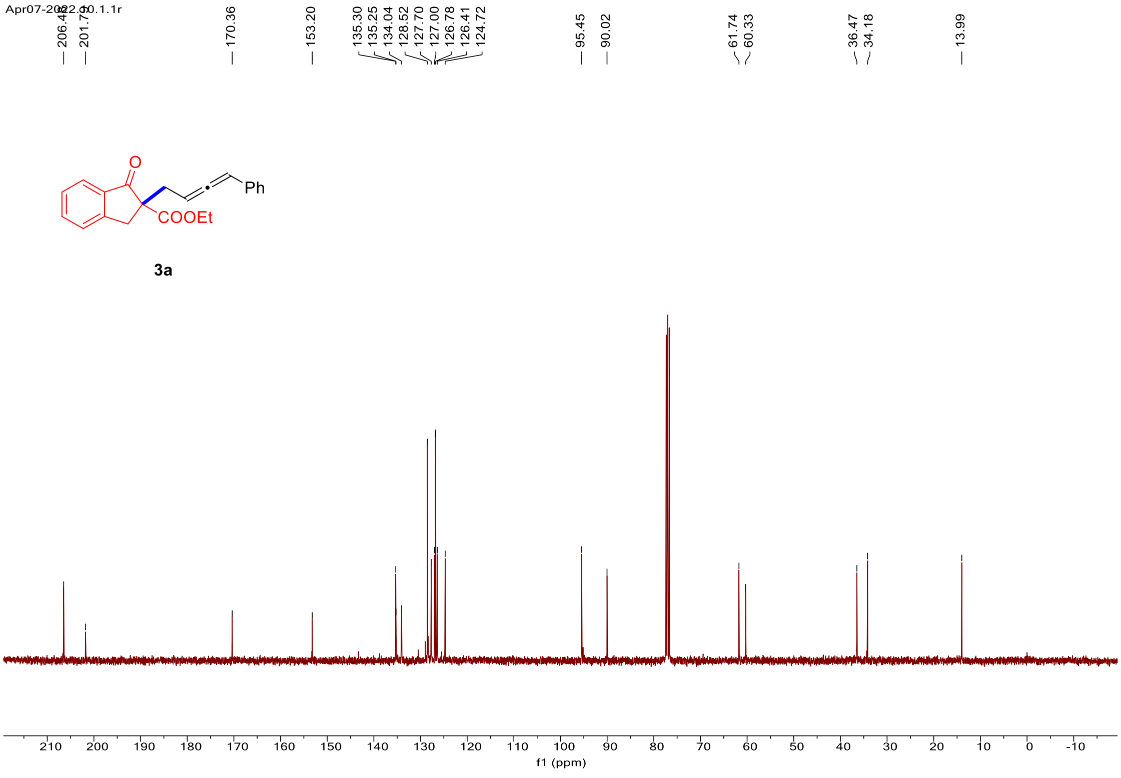

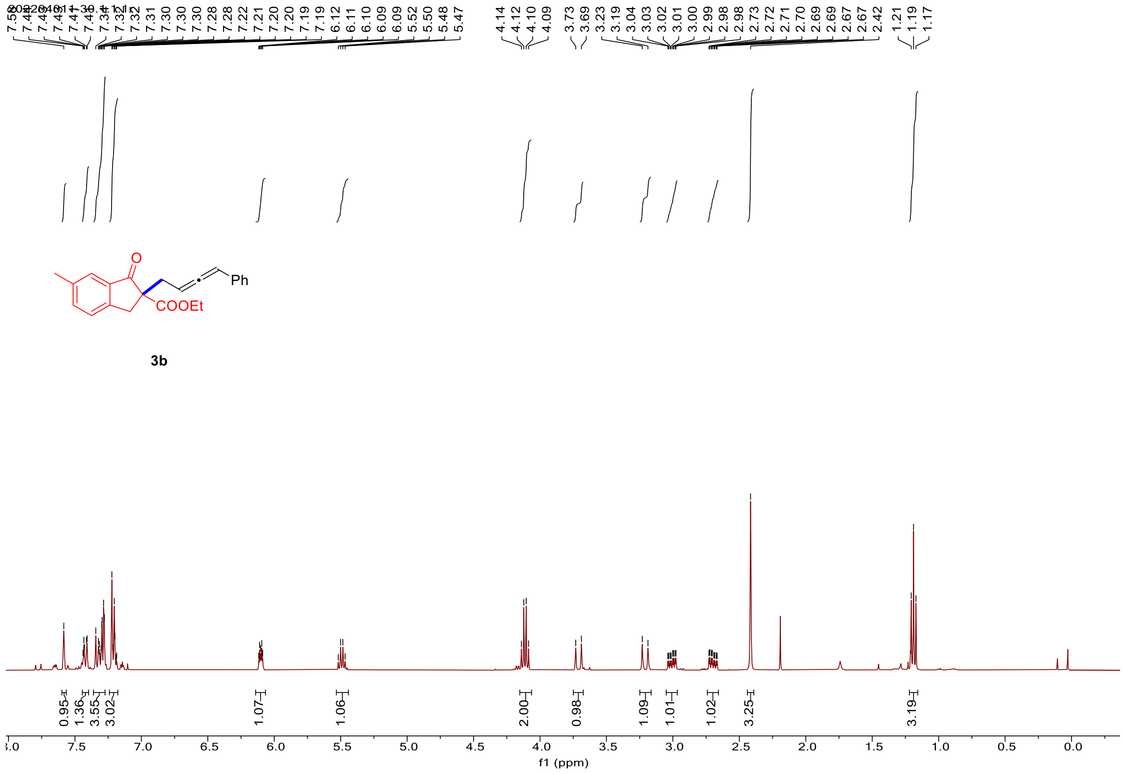
7.41
7.34
7.32
7.31
7.30
7.30
7.28
7.22
7.21
7.20
7.20
7.19
7.19
6.12
6.11
6.10
6.09
6.09
5.52
5.50
5.48
5.47
4.14
4.12
4.10
4.09
3.73
3.69
3.23
3.19
3.04
3.03
3.02
3.01
3.00
2.99
2.98
2.98
2.73
2.72
2.71
2.70
2.69
2.67
2.67
2.42
1.21
1.19
1.17
O
Ph
COOEt
3b
0.95
1.36
3.55
3.02
1.07
1.06
2.00
0.98
1.09
1.01
1.02
3.25
3.19
f1 (ppm)

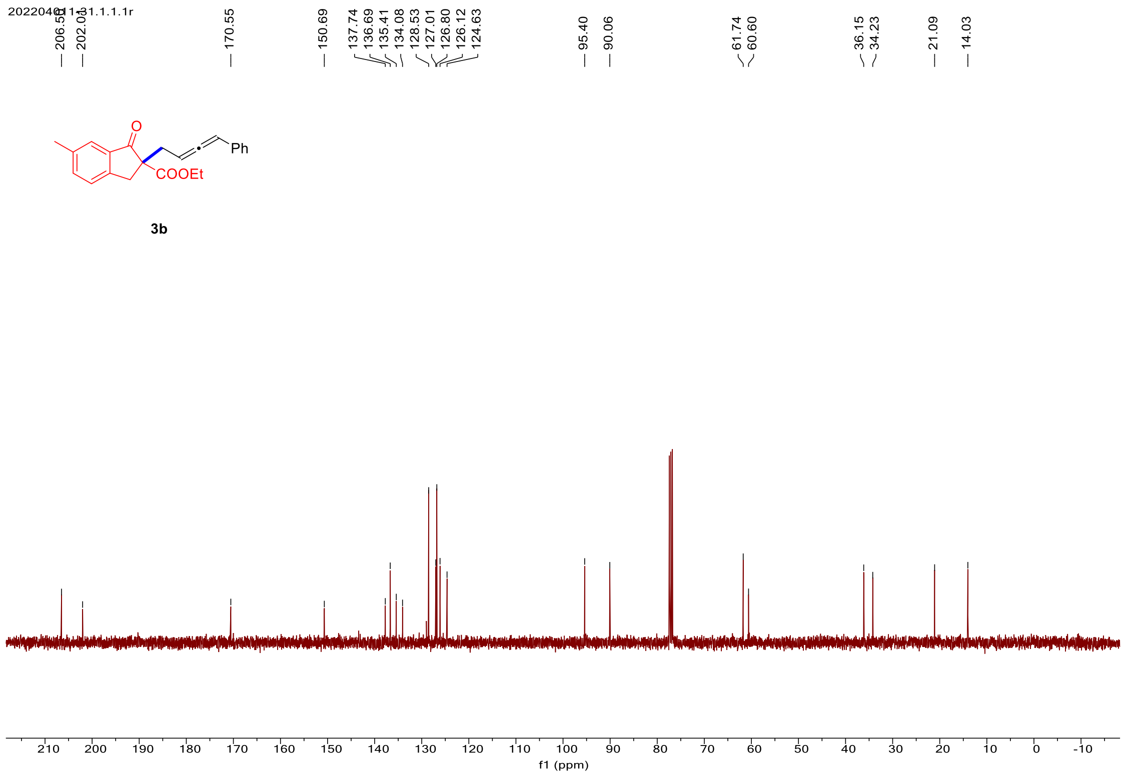
2022040111-31.1.1.1r
206.55
202.01
170.55
150.69
137.74
136.69
135.41
134.08
128.53
127.01
126.80
126.12
124.63
95.40
90.06
61.74
60.60
36.15
34.23
21.09
14.03
O
Ph
COOEt
3b
f1 (ppm)

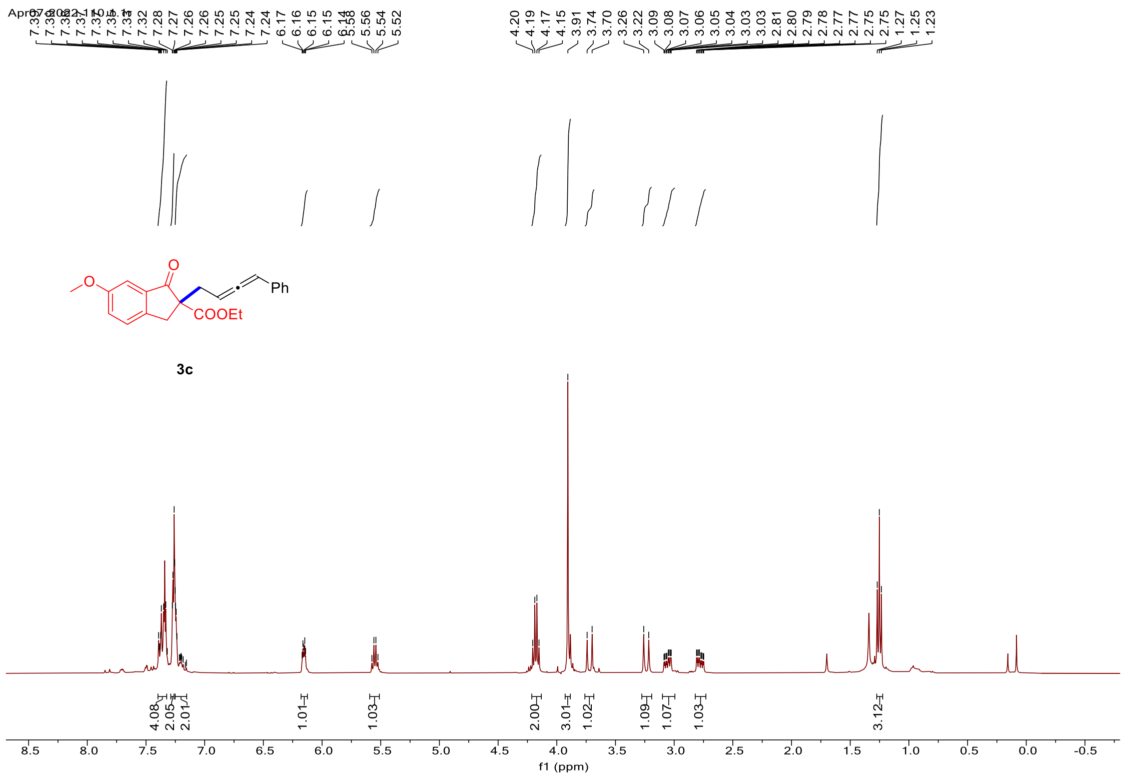
COOEt
Ph
O
3c
f1 (ppm)
4.08
2.05
2.01
1.01
1.03
2.00
3.01
1.02
1.09
1.07
1.03
3.12

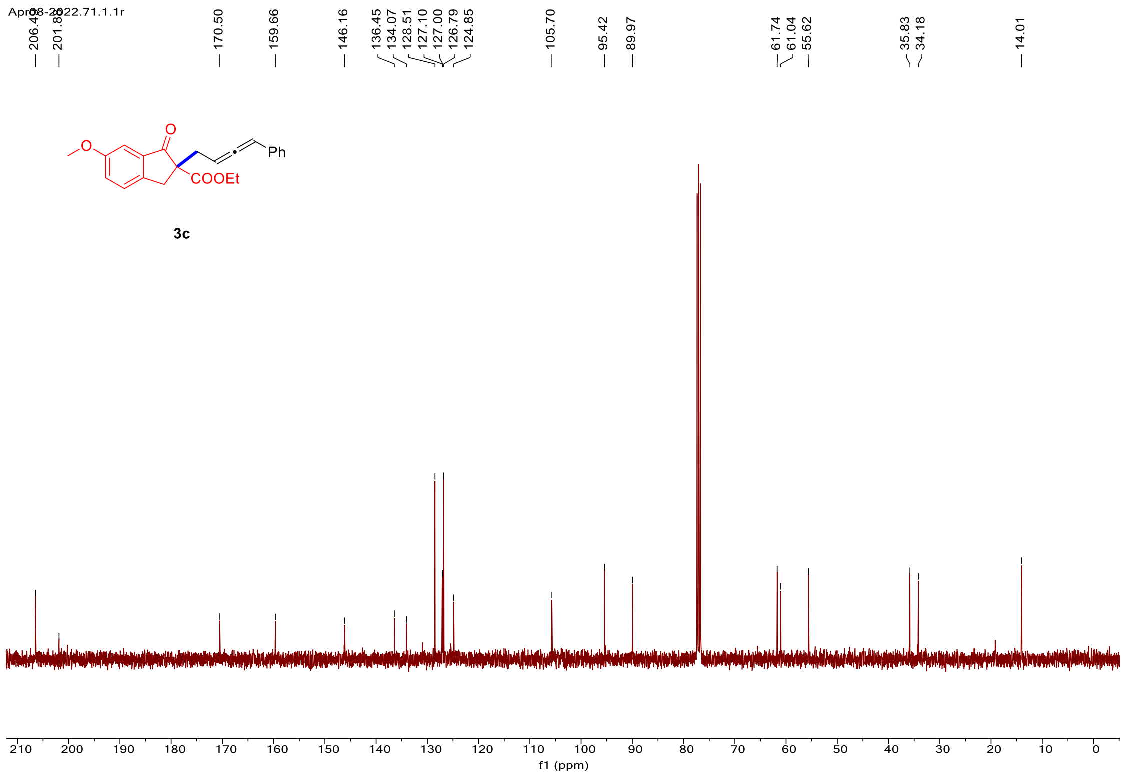
Apr08-2022.71.1.1r
170.50
159.66
146.16
136.45
134.07
128.51
127.10
127.00
126.79
124.85
105.70
95.42
89.97
61.74
61.04
55.62
35.83
34.18
14.01
COOEt
Ph
O
3c
f1 (ppm)

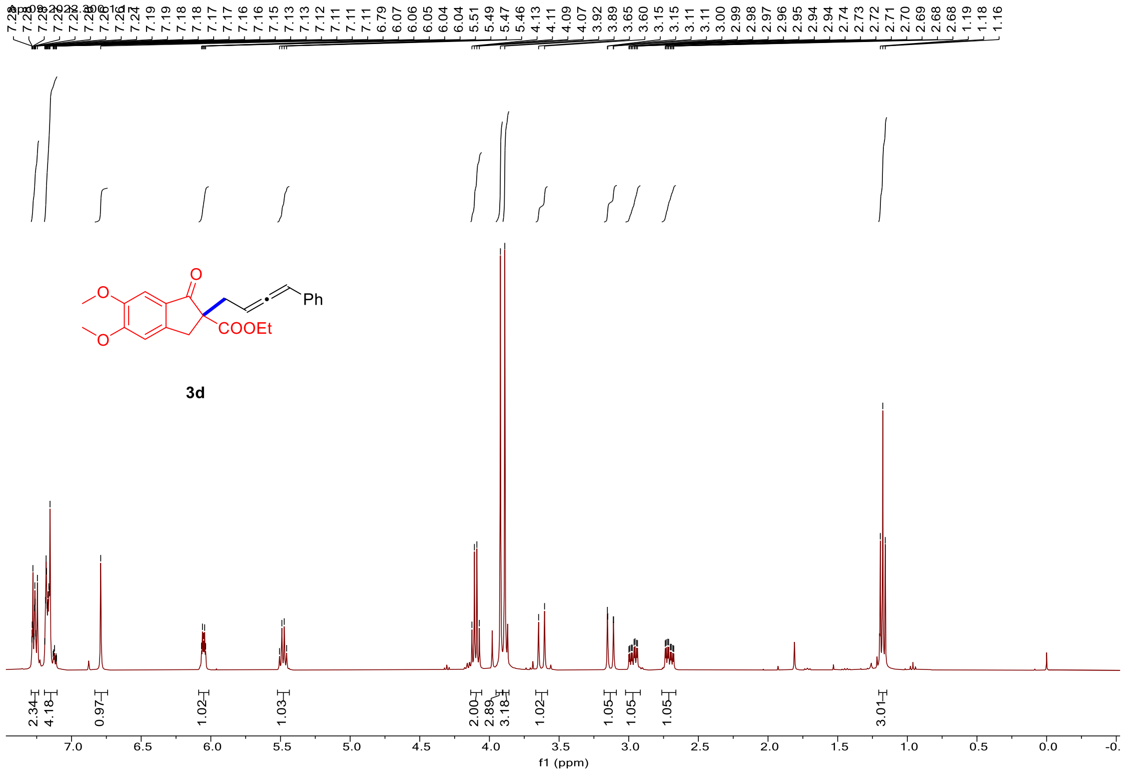
COOEt
Ph
O
3d
f1 (ppm)

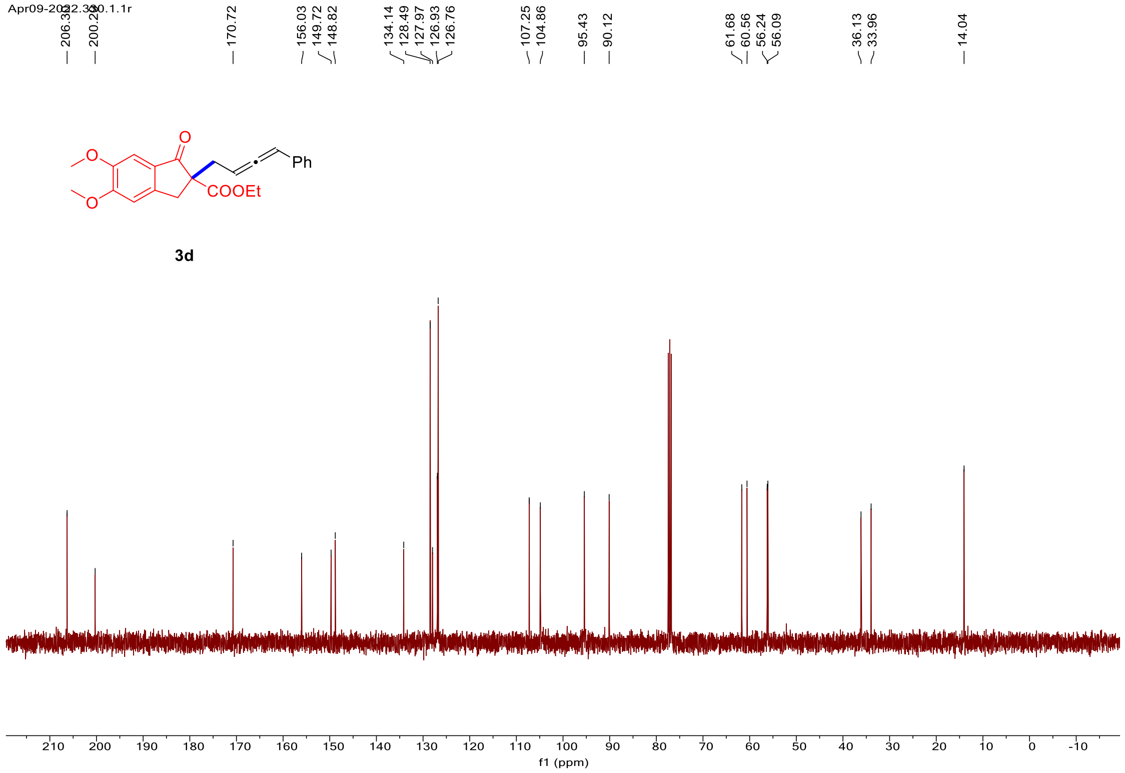
Apr09-2022.330.1.1r
170.72
156.03
149.72
148.82
134.14
128.49
127.97
126.93
126.76
107.25
104.86
95.43
90.12
61.68
60.56
56.24
56.09
36.13
33.96
14.04
COOEt
Ph
O
3d
f1 (ppm)

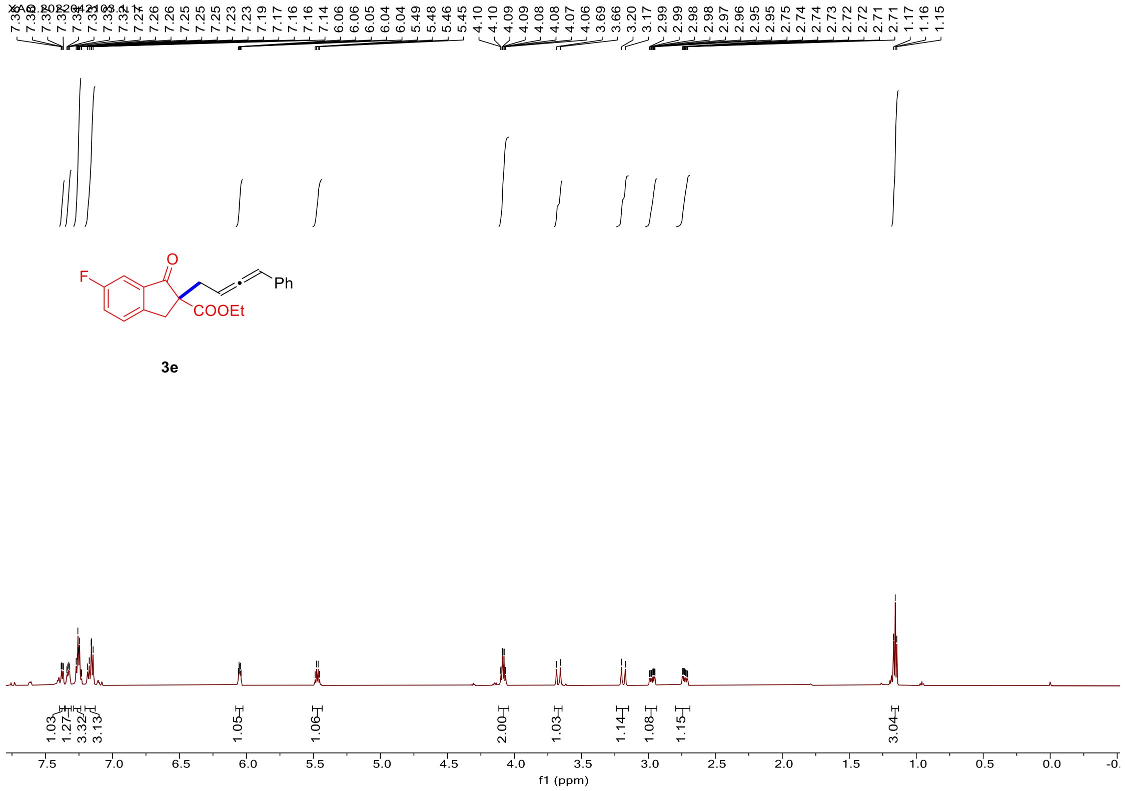
XAQ.2022042103.1.1r
7.36 7.36 7.35 7.34 7.33 7.32 7.27 7.26 7.26 7.25 7.25 7.23 7.23 7.19 7.17 7.16 7.14 6.06 6.06 6.05 6.04 6.04 5.49 5.48 5.46 5.45 4.10 4.10 4.09 4.09 4.08 4.08 4.07 4.06 3.69 3.66 3.20 3.17 2.99 2.99 2.99 2.98 2.98 2.97 2.96 2.96 2.95 2.75 2.75 2.74 2.74 2.73 2.72 2.72 2.71 2.71 1.17 1.16 1.15
F
O
Ph
COOEt
3e
1.03 1.27 3.32 3.13 1.05 1.06 2.00 1.03 1.14 1.08 1.15 3.04
7.5 7.0 6.5 6.0 5.5 5.0 4.5 4.0 3.5 3.0 2.5 2.0 1.5 1.0 0.5 0.0
f1 (ppm)

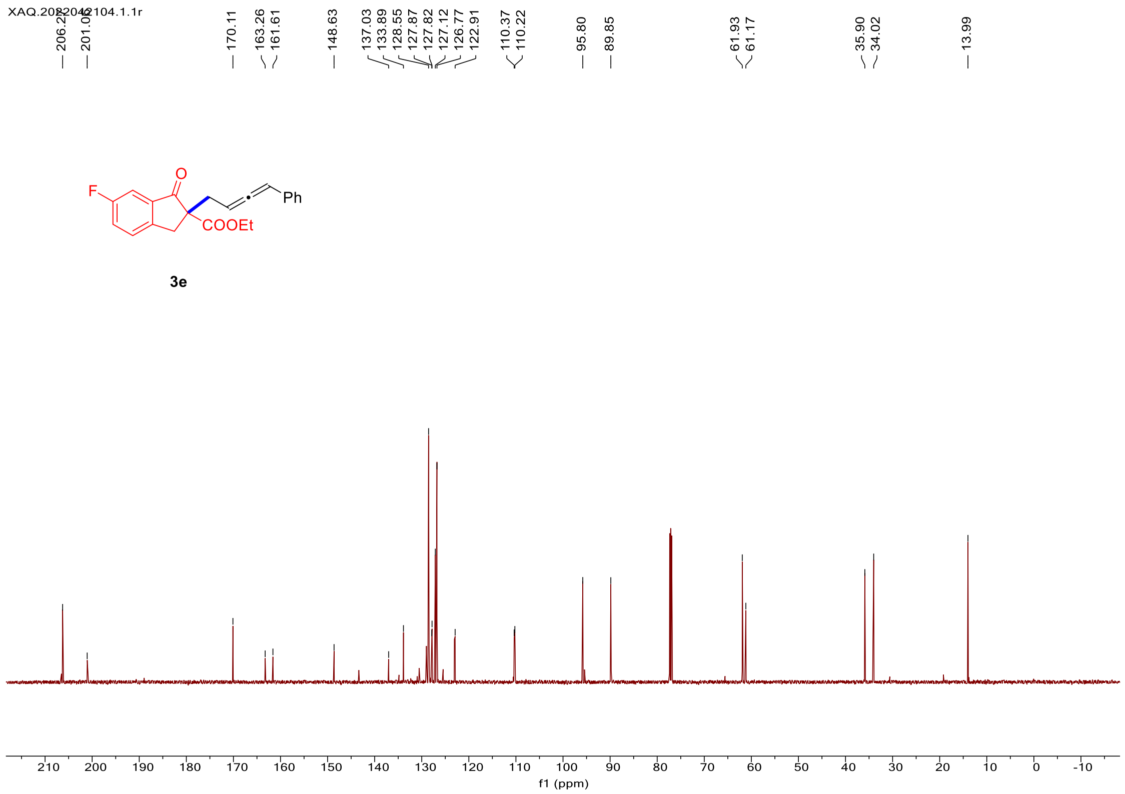
XAQ.2022042104.1.1r
206.2 201.0 170.11 163.26 161.61 148.63 137.03 133.89 128.55 127.87 127.82 127.12 126.77 122.91 110.37 110.22 95.80 89.85 61.93 61.17 35.90 34.02 13.99
F
O
Ph
COOEt
3e
210 200 190 180 170 160 150 140 130 120 110 100 90 80 70 60 50 40 30 20 10 0 -10
f1 (ppm)

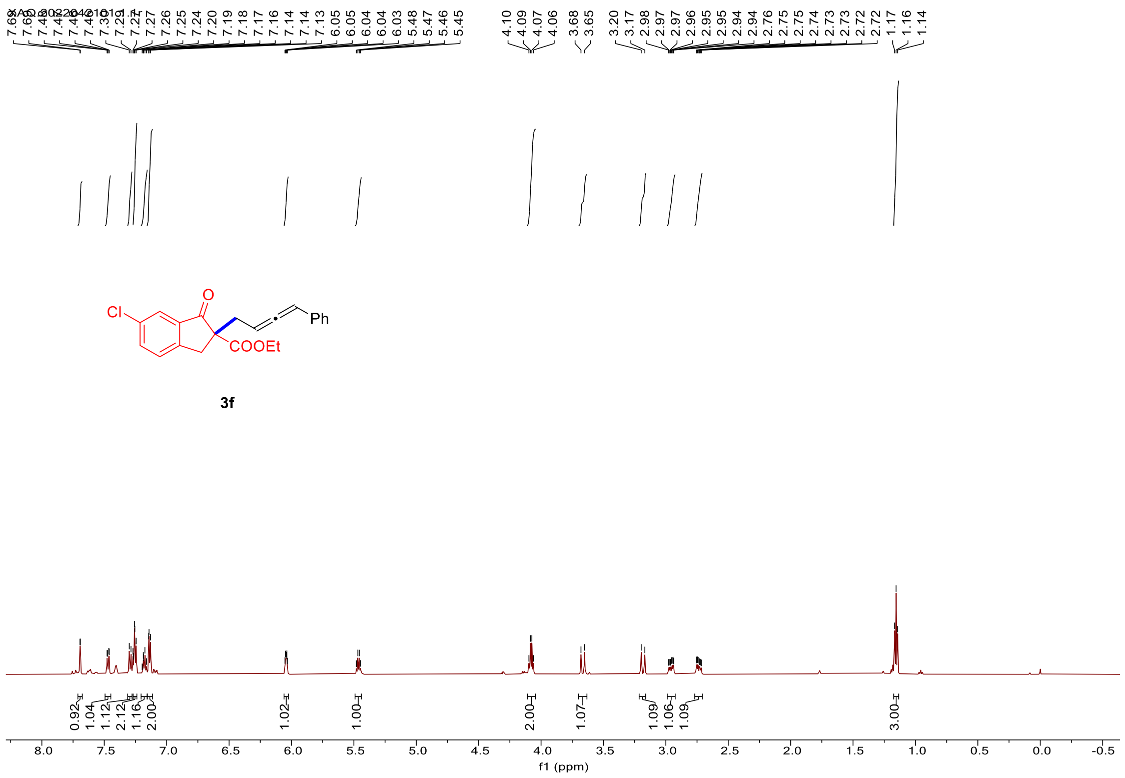
7.69 7.69 7.49 7.49 7.47 7.47 7.30 7.29 7.27 7.27 7.26 7.25 7.24 7.20 7.19 7.18 7.17 7.16 7.14 7.14 7.13 6.05 6.05 6.04 6.04 6.03 5.48 5.47 5.46 5.45
4.10 4.09 4.07 4.06 3.68 3.65 3.20 3.17 2.98 2.97 2.97 2.96 2.95 2.95 2.94 2.94 2.76 2.75 2.75 2.74 2.73 2.73 2.72 2.72 1.17 1.16 1.14
Cl
O
Ph
COOEt
0.92 1.04 1.12 2.12 1.16 2.00 1.02 1.00 2.00 1.07 1.09 1.06 1.09 3.00
8.0 7.5 7.0 6.5 6.0 5.5 5.0 4.5 4.0 3.5 3.0 2.5 2.0 1.5 1.0 0.5 0.0 -0.5
f1 (ppm)

3f

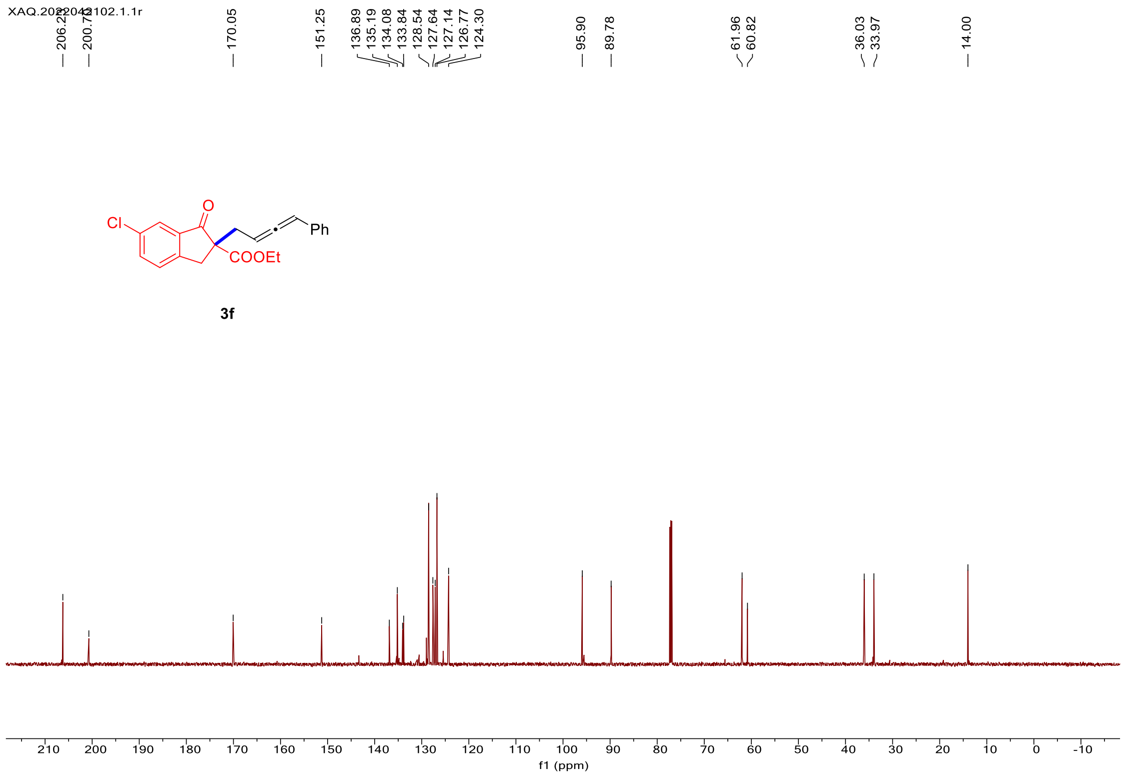
XAQ.2022042102.1.1r
206.22 200.78 170.05 151.25 136.89 135.19 134.08 133.84 128.54 127.64 127.14 126.77 124.30 95.90 89.78 61.96 60.82 36.03 33.97 14.00
Cl
O
Ph
COOEt
210 200 190 180 170 160 150 140 130 120 110 100 90 80 70 60 50 40 30 20 10 0 -10
f1 (ppm)

3f

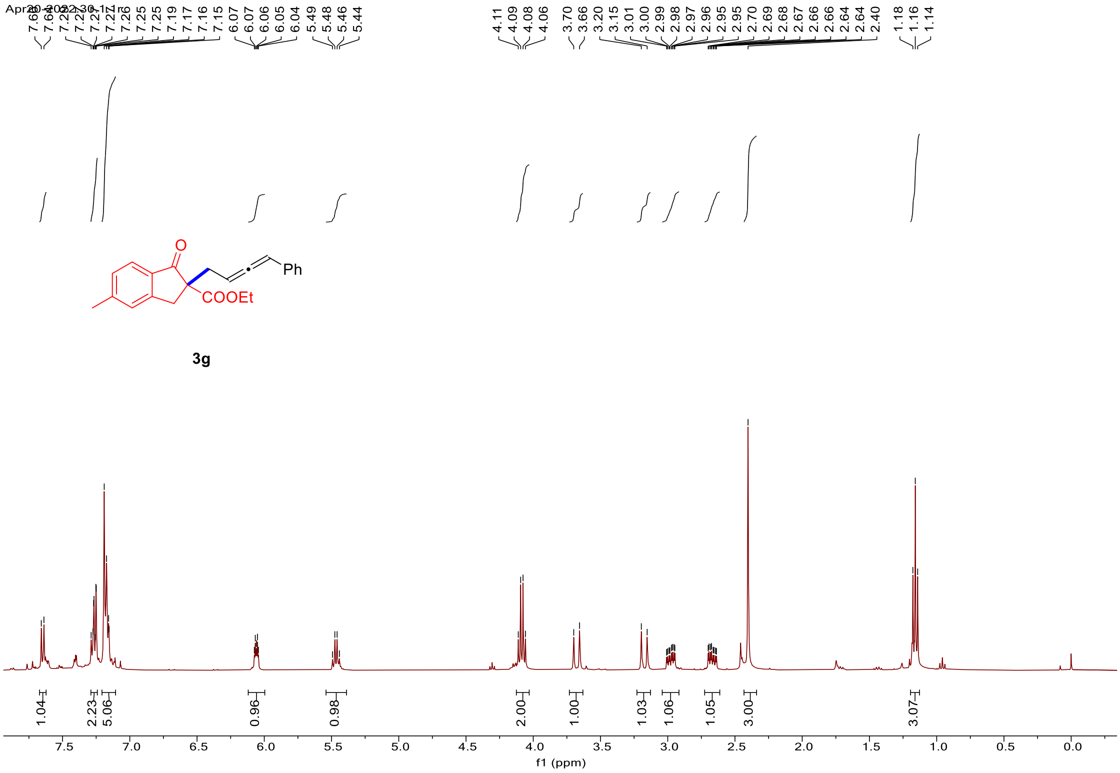

4.11 4.09 4.08 4.06 3.70 3.66 3.20 3.15 3.01 3.00 2.99 2.98 2.97 2.96 2.95 2.95 2.70 2.69 2.68 2.67 2.66 2.64 2.64 2.40 1.18 1.16 1.14
7.26 7.25 7.19 7.17 7.16 7.15 6.07 6.07 6.06 6.05 6.04 5.49 5.48 5.46 5.44
O
Ph
COOEt
3g
1.04 2.23 5.06 0.96 0.98 2.00 1.00 1.03 1.06 1.05 3.00 3.07
f1 (ppm)

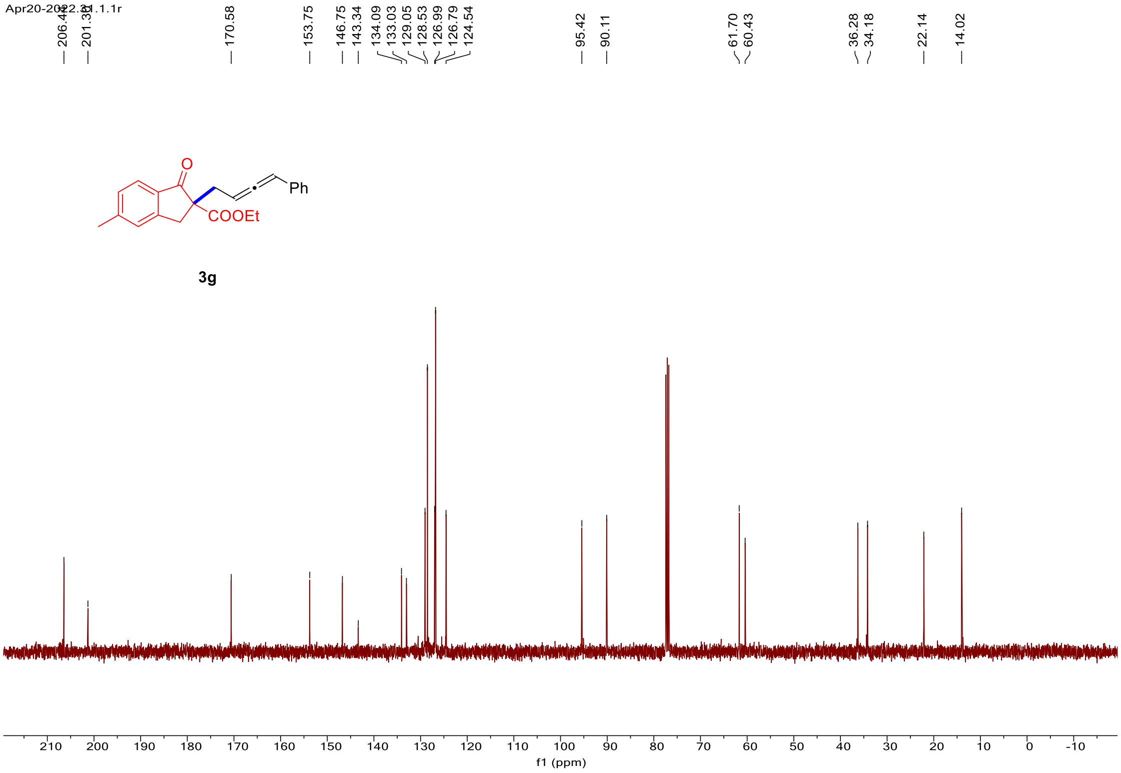

Apr20-2022.31.1.1r
206.42 201.39 170.58 153.75 146.75 143.34 134.09 133.03 129.05 128.53 126.99 126.79 124.54 95.42 90.11 61.70 60.43 36.28 34.18 22.14 14.02
O
Ph
COOEt
3g
f1 (ppm)

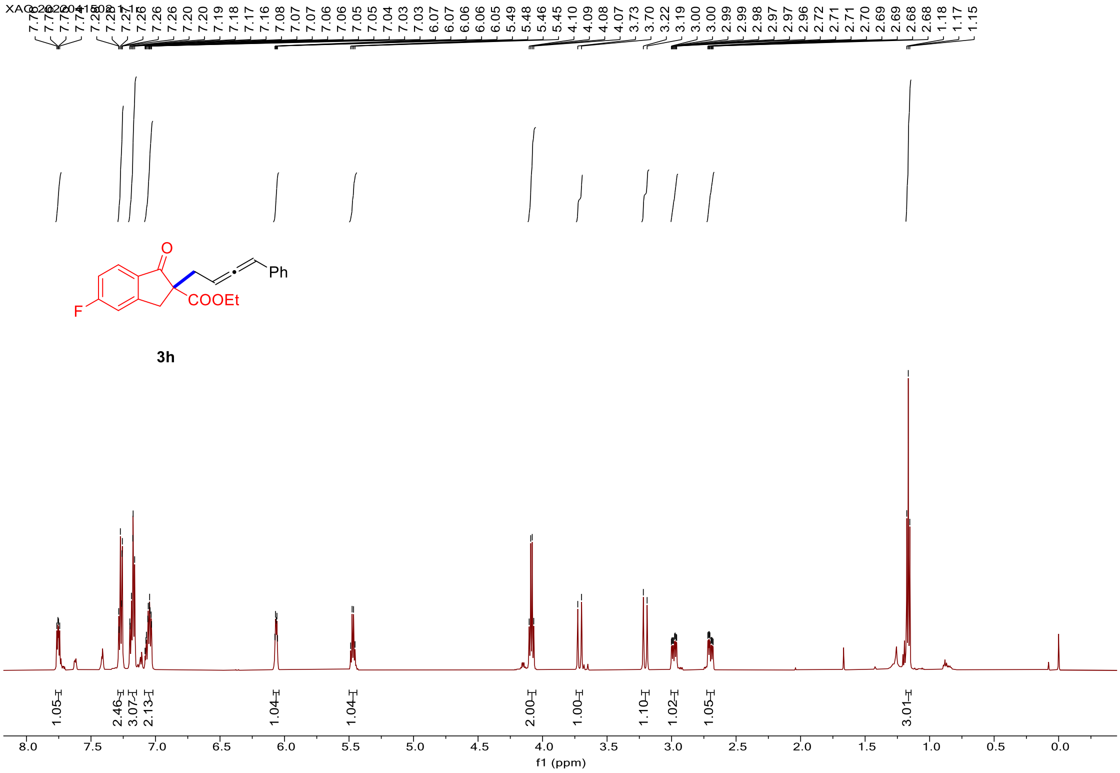
XAQ.2022041505.1.1
7.26 7.26 7.20 7.20 7.19 7.18 7.17 7.16 7.08 7.07 7.07 7.06 7.06 7.05 7.05 7.04 7.03 6.07 6.07 6.06 6.06 6.05 5.49 5.48 5.46 5.45 4.10 4.09 4.08 4.07 3.73 3.70 3.22 3.19 3.00 3.00 2.99 2.99 2.98 2.97 2.97 2.96 2.72 2.71 2.71 2.70 2.69 2.69 2.68 1.18 1.17 1.15
O
Ph
F
COOEt
3h
1.05 2.46 3.07 2.13 1.04 1.04 2.00 1.00 1.10 1.02 1.05 3.01
8.0 7.5 7.0 6.5 6.0 5.5 5.0 4.5 4.0 3.5 3.0 2.5 2.0 1.5 1.0 0.5 0.0
f1 (ppm)

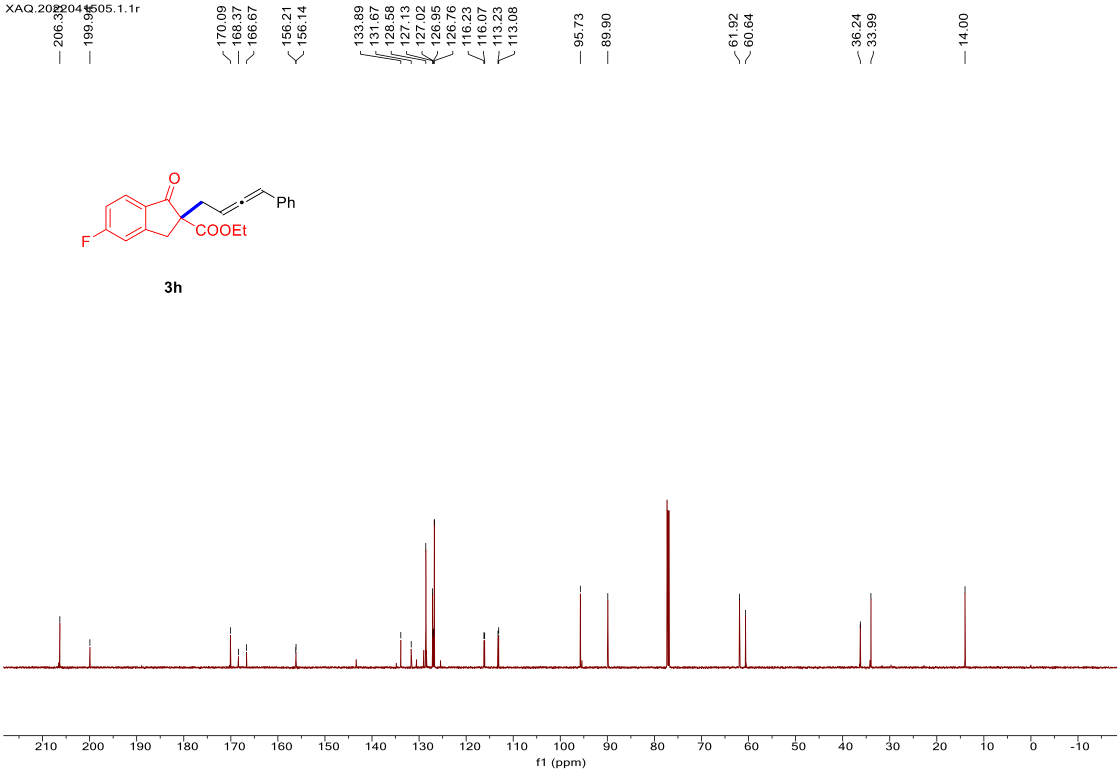
XAQ.2022041505.1.1r
206.3 199.9 170.09 168.37 166.67 156.21 156.14 133.89 131.67 128.58 127.13 127.02 126.95 126.76 116.23 116.07 113.23 113.08 95.73 89.90 61.92 60.64 36.24 33.99 14.00
O
Ph
F
COOEt
3h
210 200 190 180 170 160 150 140 130 120 110 100 90 80 70 60 50 40 30 20 10 0 -10
f1 (ppm)

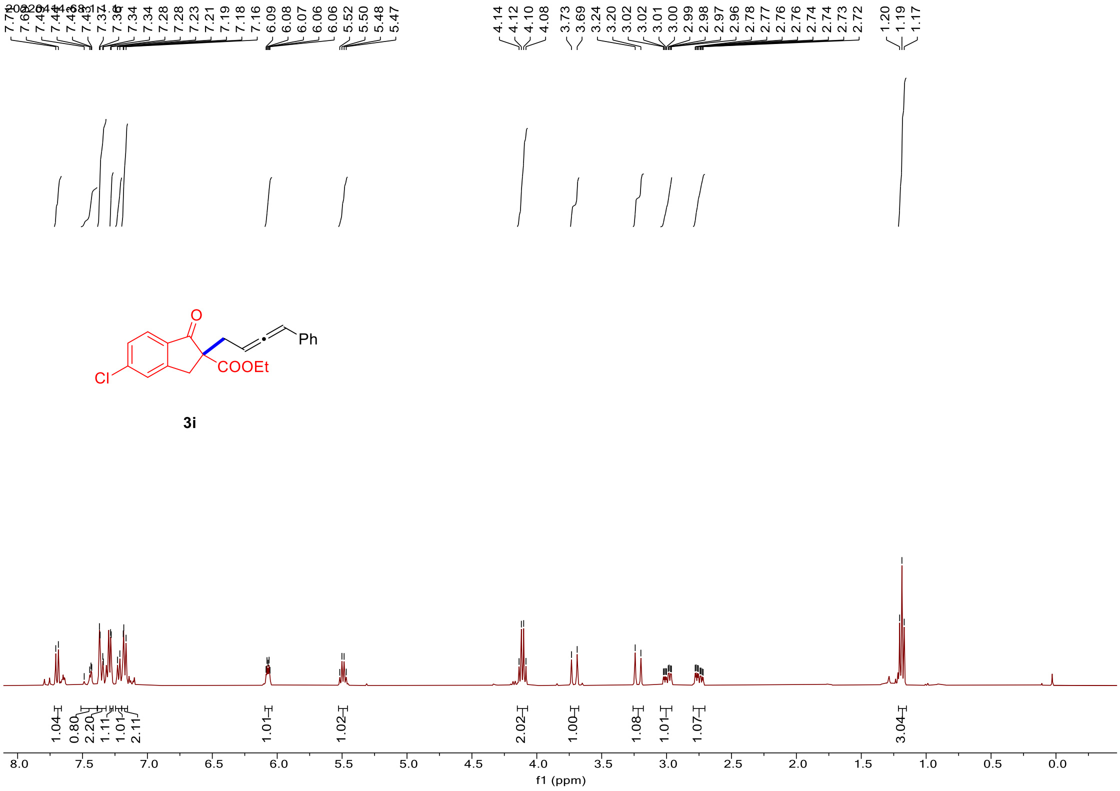
7.44
7.41
7.34
7.34
7.28
7.28
7.23
7.21
7.19
7.18
7.16
6.09
6.08
6.07
6.06
6.06
5.52
5.50
5.48
5.47
4.14
4.12
4.10
4.08
3.73
3.69
3.24
3.20
3.02
3.01
3.00
2.99
2.98
2.97
2.96
2.78
2.77
2.76
2.76
2.74
2.73
2.72
1.20
1.19
1.17
Ph
COOEt
Cl
O
3i
1.04
0.80
2.20
1.11
1.01
2.11
1.01
1.02
2.02
1.00
1.08
1.01
1.07
3.04
f1 (ppm)

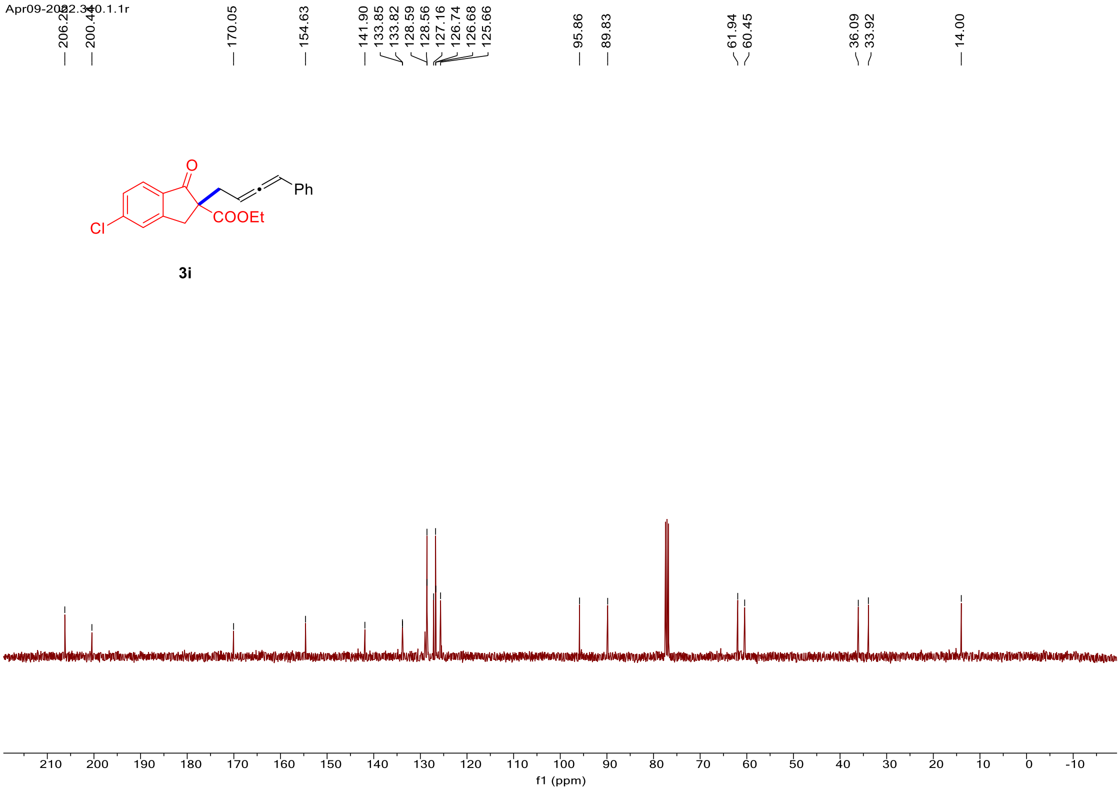
Apr09-2022.340.1.1r
206.2
200.4
170.05
154.63
141.90
133.85
133.82
128.59
128.56
127.16
126.74
126.68
125.66
95.86
89.83
61.94
60.45
36.09
33.92
14.00
Ph
COOEt
Cl
O
3i
f1 (ppm)

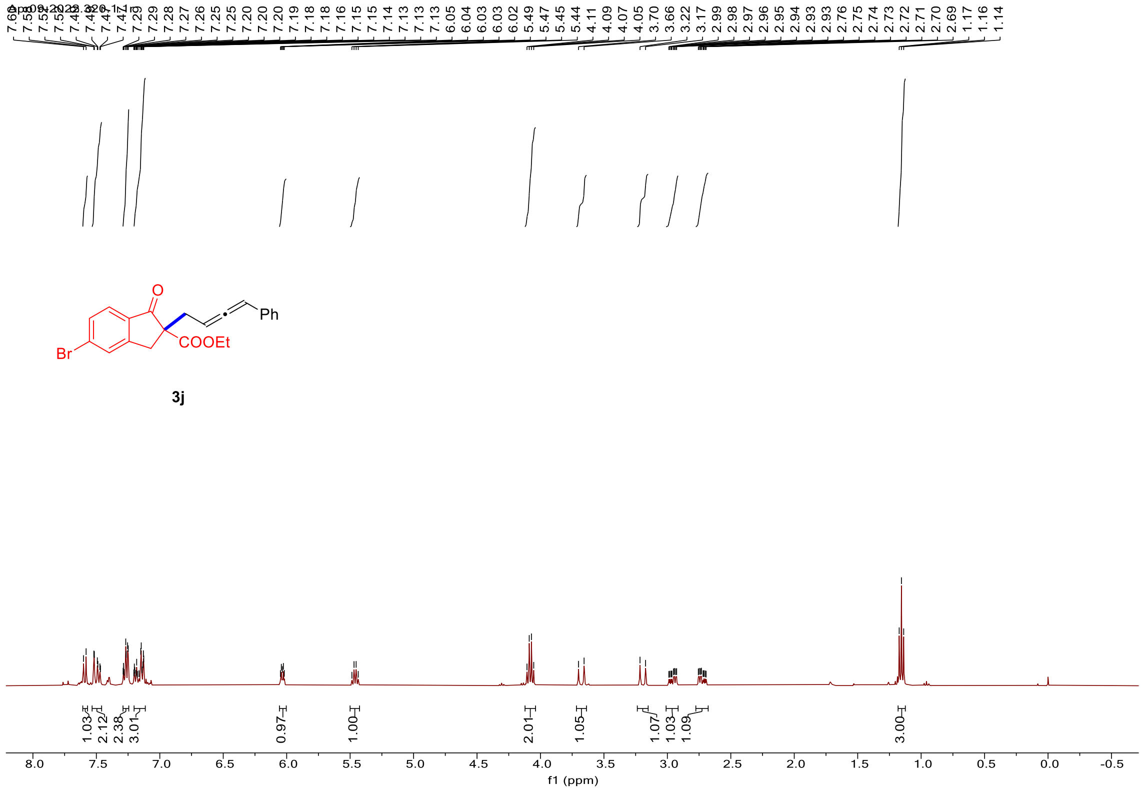
7.59
7.52
7.49
7.47
7.29
7.29
7.28
7.27
7.26
7.25
7.25
7.20
7.20
7.20
7.19
7.18
7.18
7.16
7.15
7.15
7.14
7.13
7.13
7.13
6.05
6.04
6.03
6.03
6.02
5.49
5.47
5.45
5.44
4.11
4.09
4.07
4.05
3.70
3.66
3.22
3.17
2.99
2.98
2.97
2.96
2.95
2.94
2.93
2.93
2.76
2.75
2.74
2.73
2.72
2.71
2.70
2.69
1.17
1.16
1.14
O
Ph
Br
COOEt
3j
1.03
2.12
2.38
3.01
0.97
1.00
2.01
1.05
1.07
1.03
1.09
3.00
f1 (ppm)

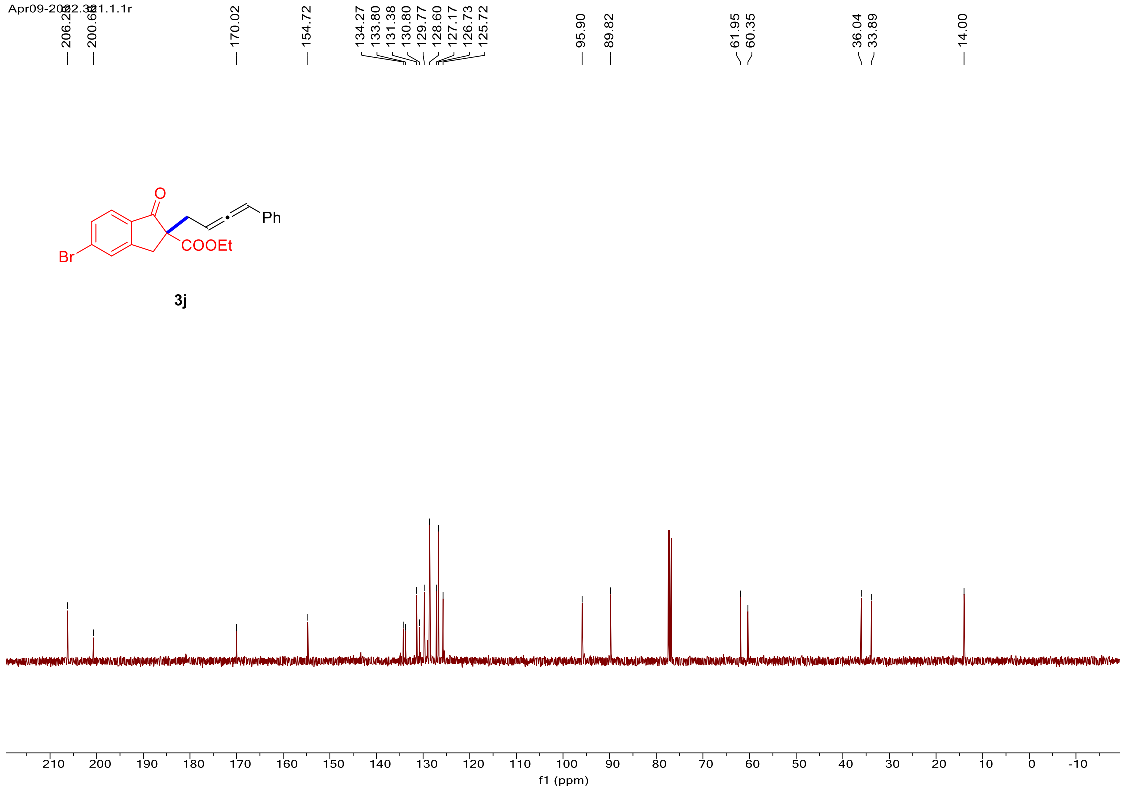
206.2
200.6
170.02
154.72
134.27
133.80
131.38
130.80
129.77
128.60
127.17
126.73
125.72
95.90
89.82
61.95
60.35
36.04
33.89
14.00
O
Ph
Br
COOEt
3j
f1 (ppm)

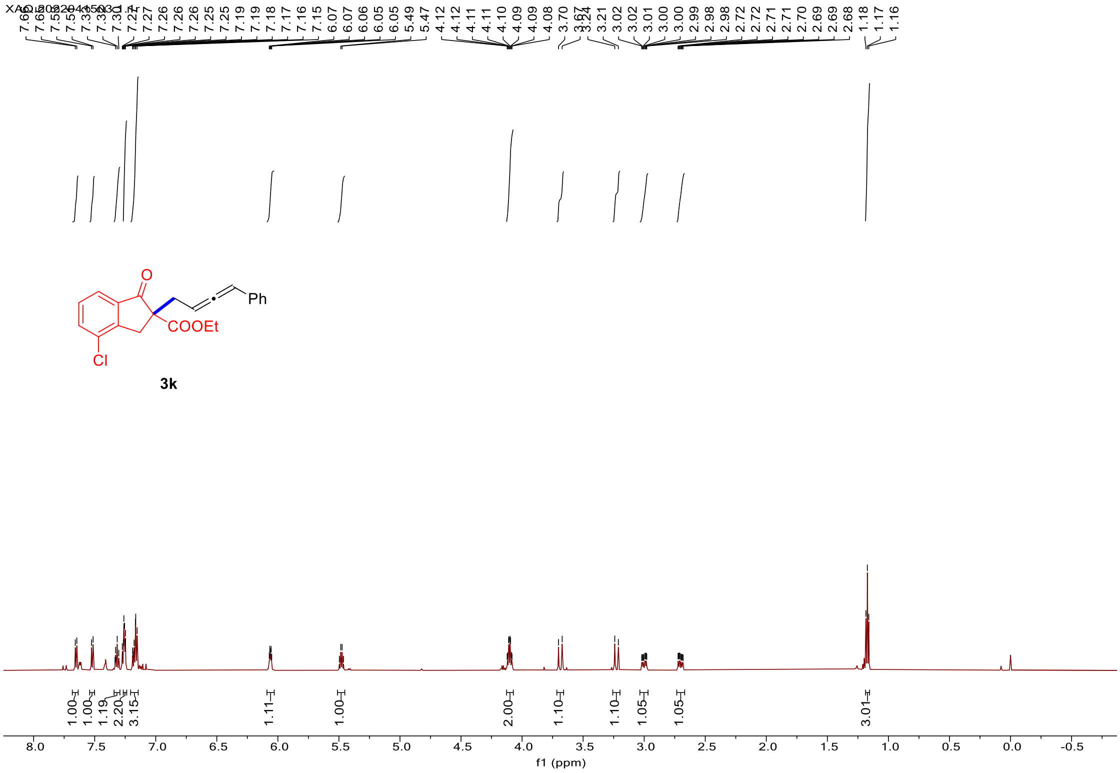
7.62 7.60 7.52 7.50 7.33 7.31 7.30 7.27 7.26 7.26 7.26 7.25 7.25 7.19 7.19 7.18 7.17 7.16 7.15 6.07 6.07 6.06 6.05 6.05 5.49 5.47 4.12 4.12 4.11 4.11 4.10 4.09 4.09 4.08 3.70 3.67 3.24 3.21 3.02 3.02 3.01 3.00 3.00 2.99 2.98 2.98 2.72 2.72 2.71 2.71 2.70 2.69 2.69 2.68 1.18 1.17 1.16
O
Ph
COOEt
Cl
3k
1.00 1.00 1.19 2.20 3.15 1.11 1.00 2.00 1.10 1.10 1.05 1.05 3.01
8.0 7.5 7.0 6.5 6.0 5.5 5.0 4.5 4.0 3.5 3.0 2.5 2.0 1.5 1.0 0.5 0.0 -0.5
f1 (ppm)

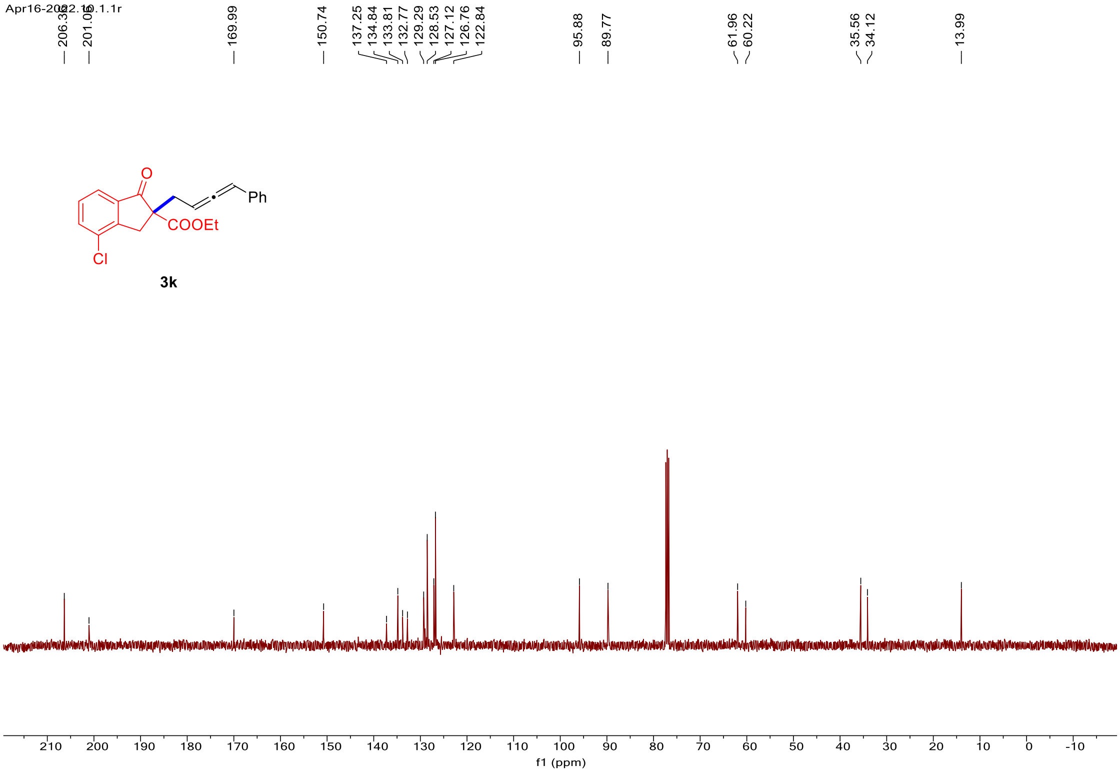
Apr16-2022.10.1.1r
206.36 201.06 169.99 150.74 137.25 134.84 133.81 132.77 129.29 128.53 127.12 126.76 122.84 95.88 89.77 61.96 60.22 35.56 34.12 13.99
O
Ph
COOEt
Cl
3k
210 200 190 180 170 160 150 140 130 120 110 100 90 80 70 60 50 40 30 20 10 0 -10
f1 (ppm)

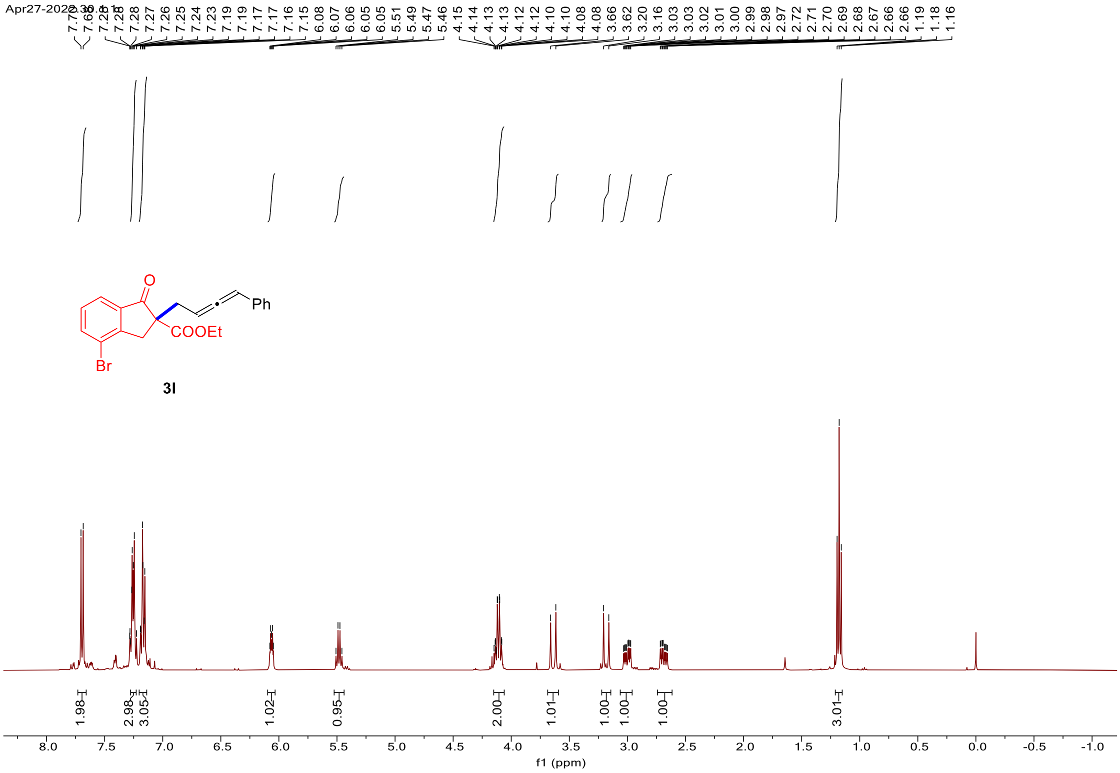
O
Ph
COOEt
Br
3l
1.98
2.98
3.05
1.02
0.95
2.00
1.01
1.00
1.00
1.00
3.01
f1 (ppm)

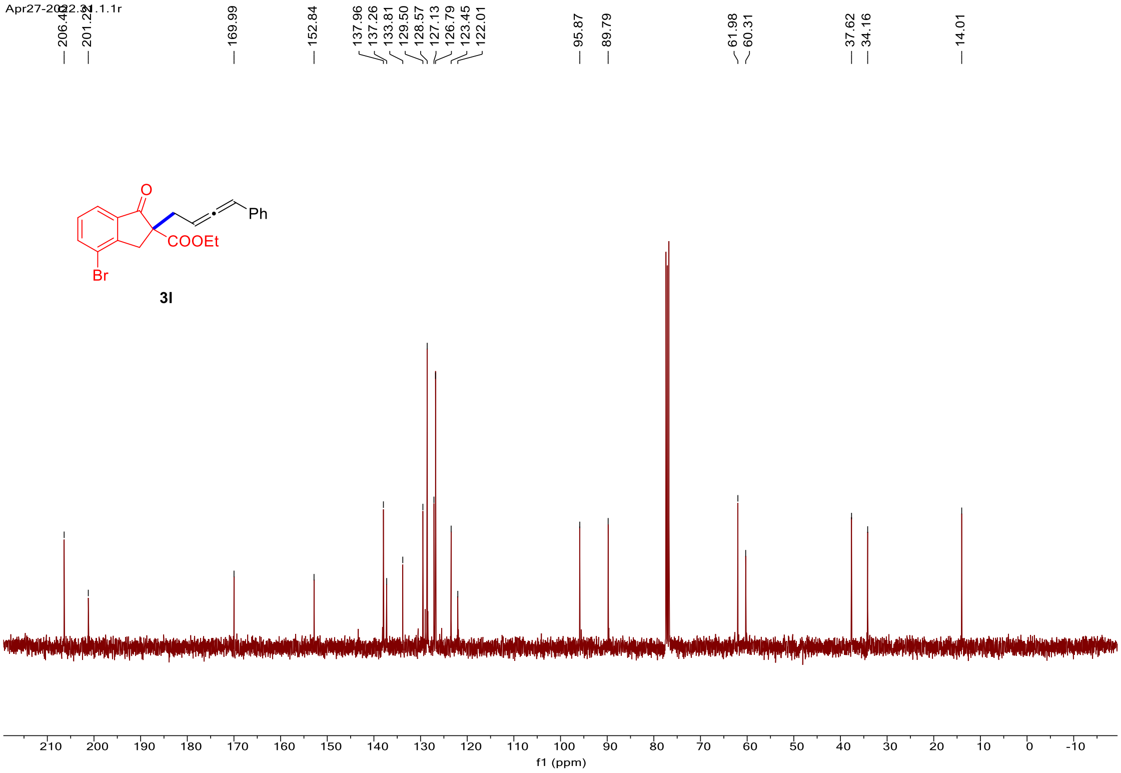
206.48
201.22
169.99
152.84
137.96
137.26
133.81
129.50
128.57
127.13
126.79
123.45
122.01
95.87
89.79
61.98
60.31
37.62
34.16
14.01
O
Ph
COOEt
Br
3l
f1 (ppm)

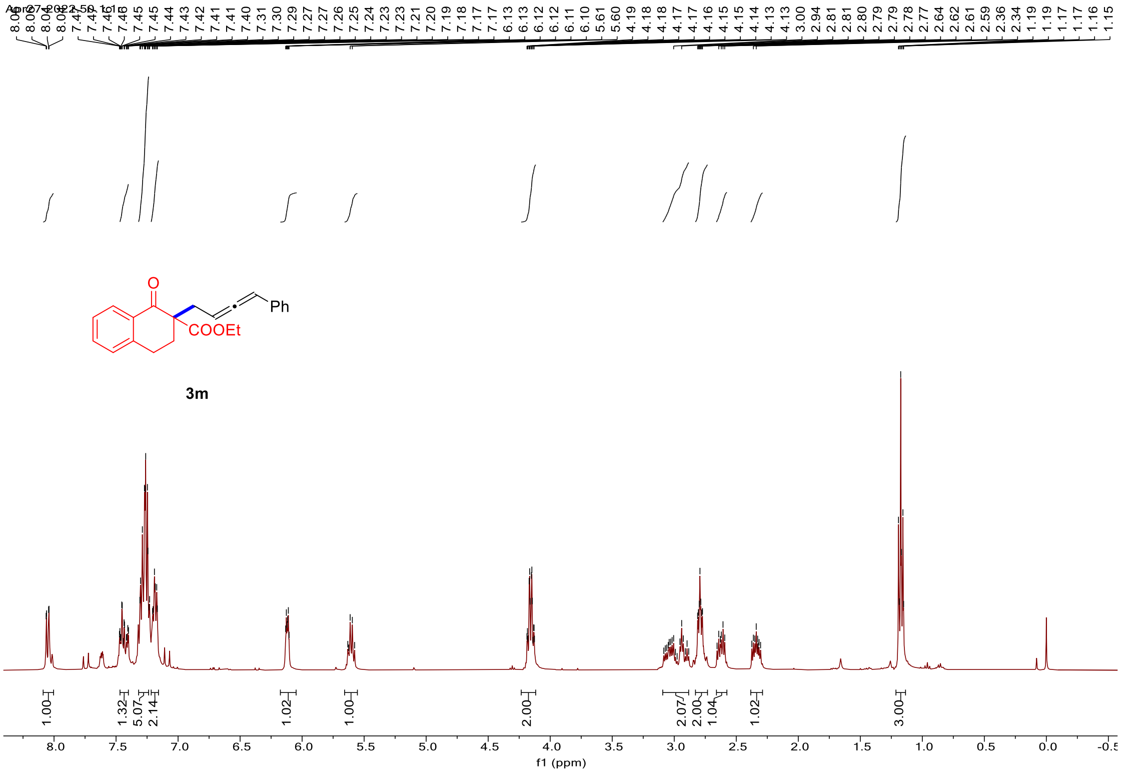
O
Ph
COOEt
3m
1.00
1.32
5.07
2.14
1.02
1.00
2.00
2.07
2.00
1.04
1.02
3.00
f1 (ppm)

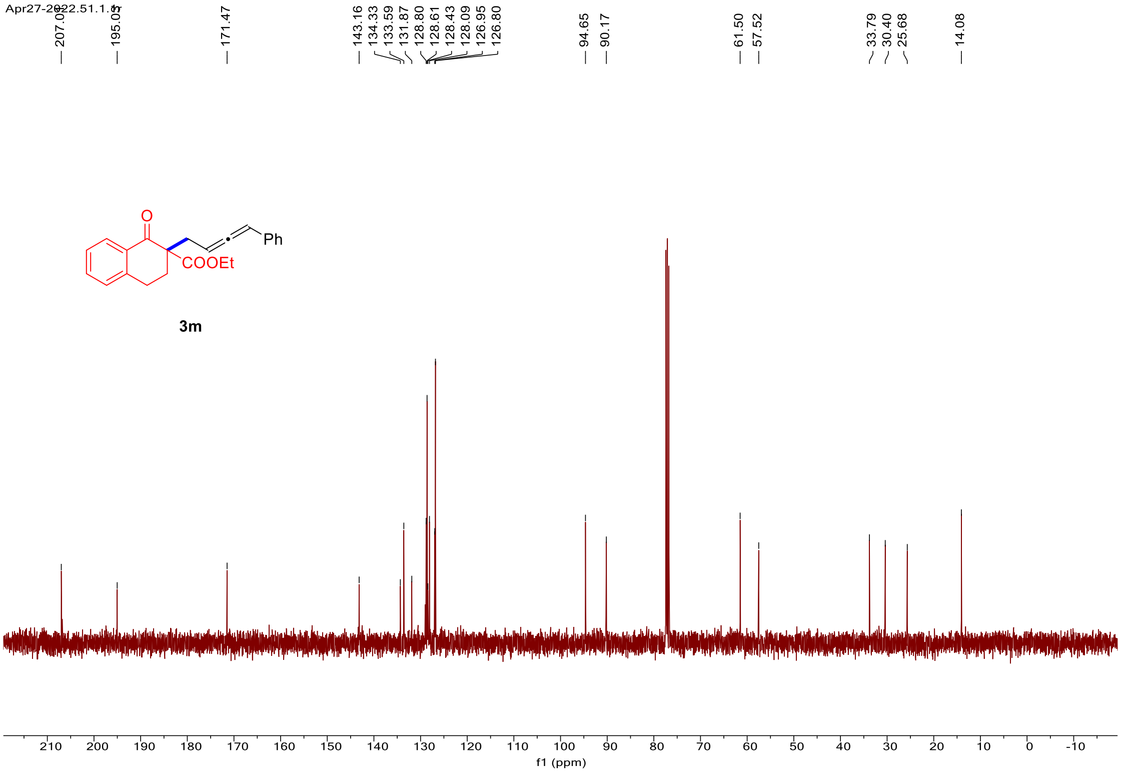
Apr27-2022.51.1.fid
207.09
195.05
171.47
143.16
134.33
133.59
131.87
128.80
128.61
128.43
128.09
126.95
126.80
94.65
90.17
61.50
57.52
33.79
30.40
25.68
14.08
O
Ph
COOEt
3m
f1 (ppm)

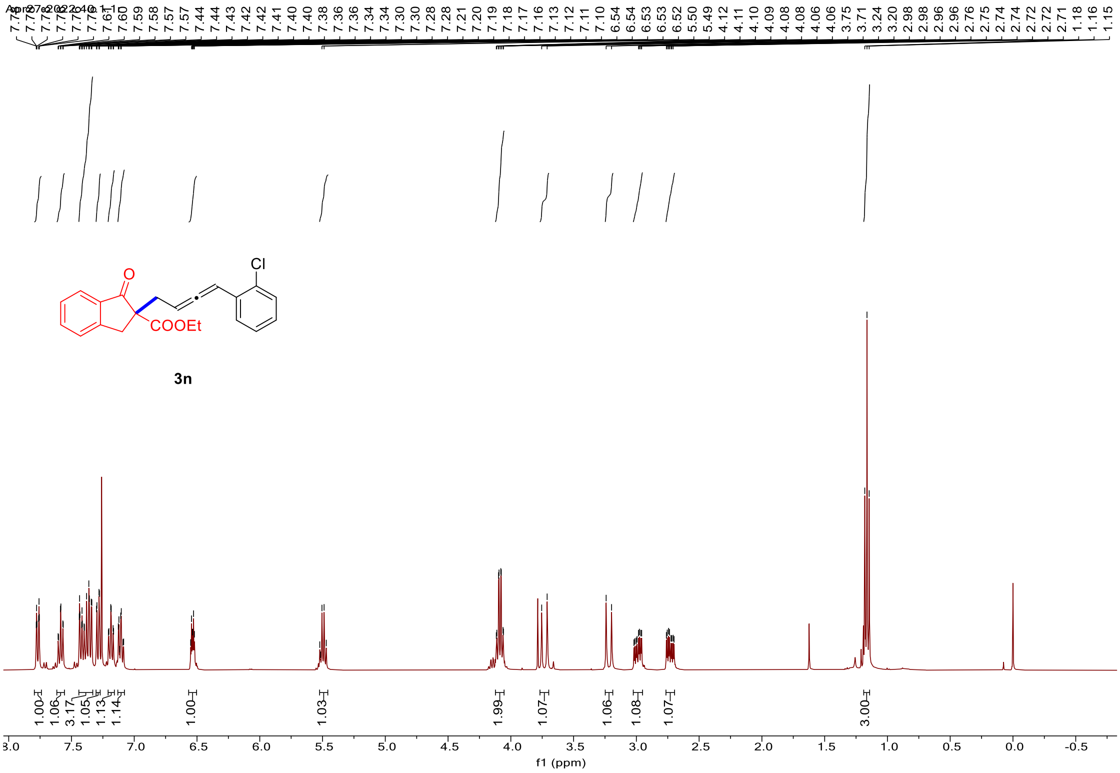
O
Cl
COOEt
3n
1.00
1.06
3.17
1.05
1.13
1.14
1.00
1.03
1.99
1.07
1.06
1.08
1.07
3.00
f1 (ppm)

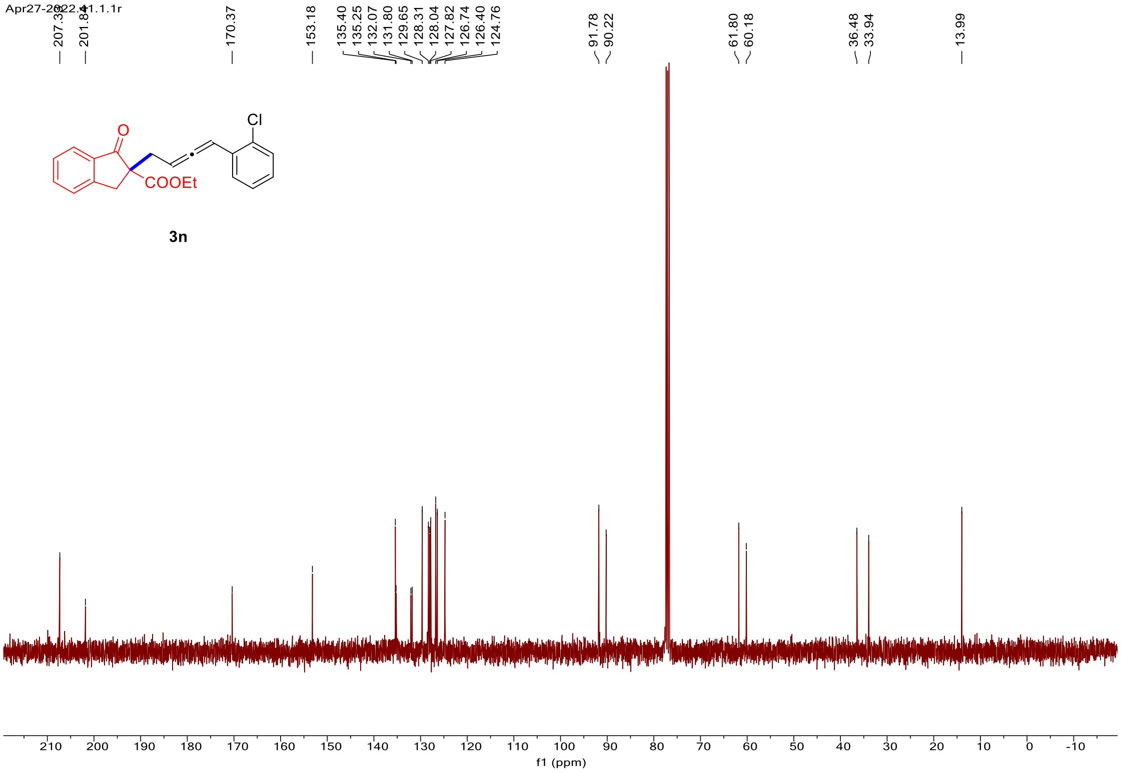
Apr27-2022.41.1.1r
207.39
201.84
170.37
153.18
135.40
135.25
132.07
131.80
129.65
128.31
128.04
127.82
126.74
126.40
124.76
91.78
90.22
61.80
60.18
36.48
33.94
13.99
O
Cl
COOEt
3n
f1 (ppm)

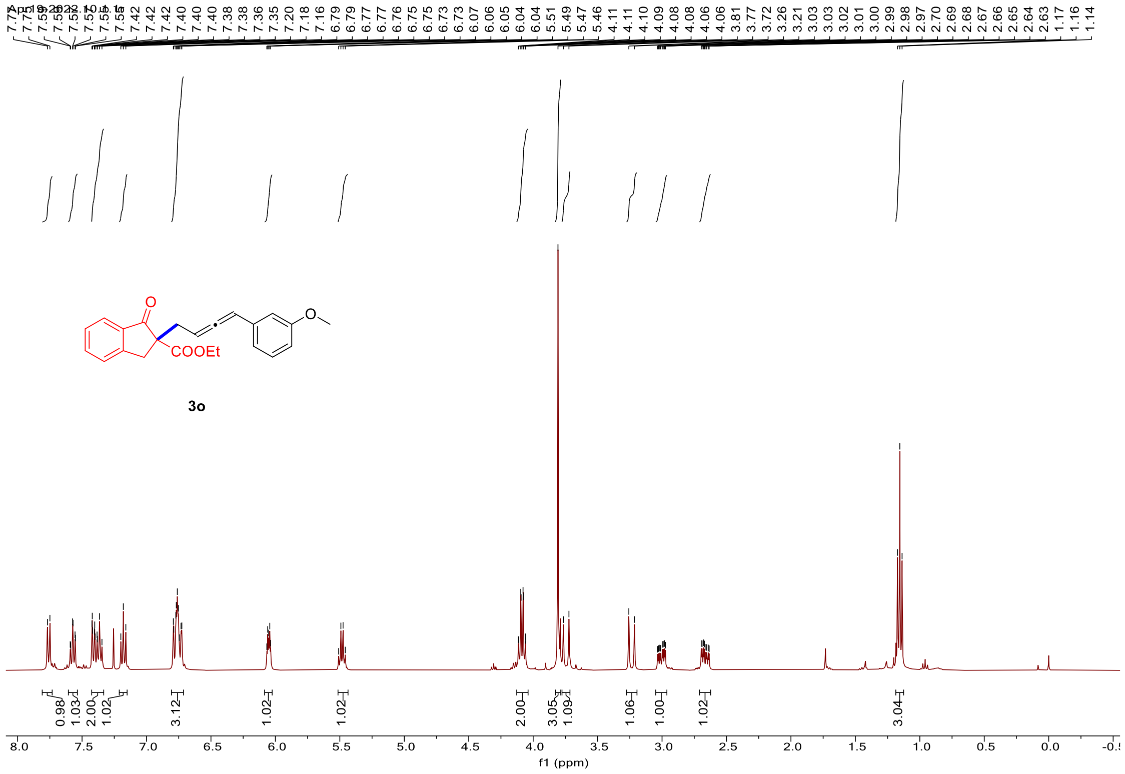
7.42 7.42 7.40 7.40 7.40 7.38 7.38 7.36 7.35 7.20 7.18 7.16 6.79 6.79 6.77 6.77 6.76 6.75 6.75 6.73 6.73 6.07 6.06 6.05 6.04 6.04 5.51 5.49 5.47 5.46 4.11 4.11 4.10 4.09 4.08 4.08 4.06 4.06 3.81 3.77 3.72 3.26 3.21 3.03 3.03 3.02 3.01 3.00 2.99 2.98 2.97 2.70 2.69 2.68 2.67 2.66 2.65 2.64 2.63 1.17 1.16 1.14
O
COOEt
O
3o
0.98
1.03
2.00
1.02
3.12
1.02
1.02
2.00
3.05
1.09
1.06
1.00
1.02
3.04
f1 (ppm)

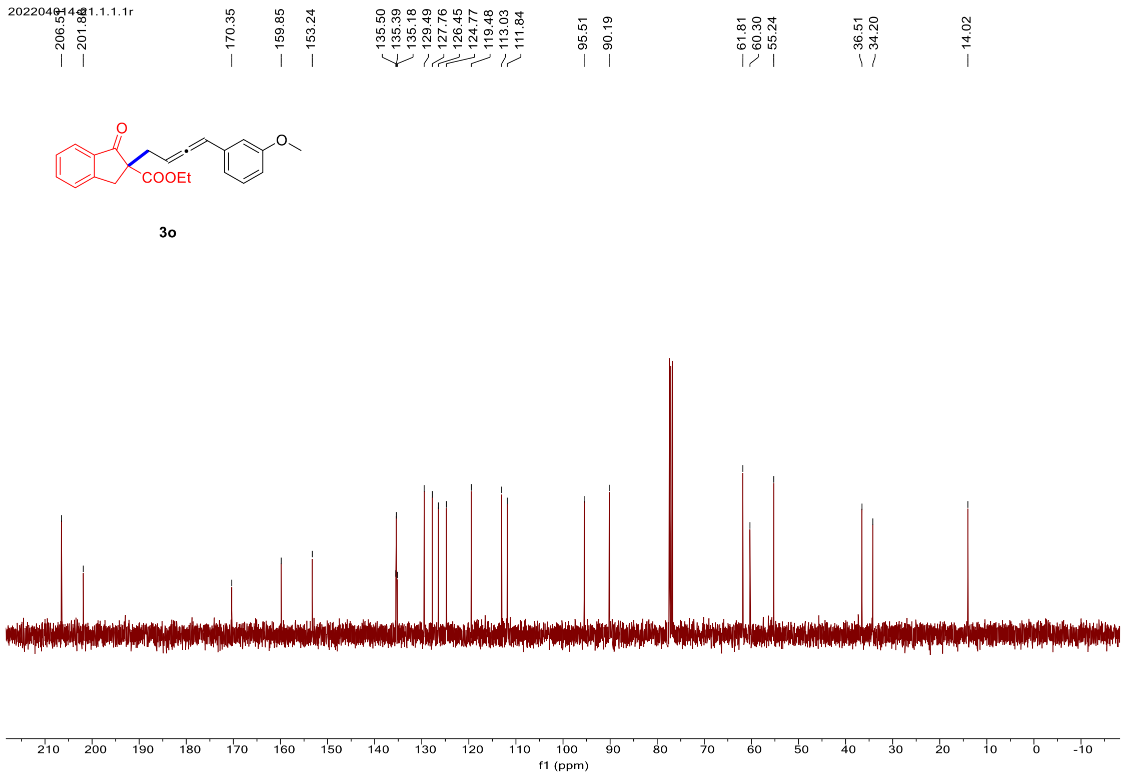
170.35 159.85 153.24 135.50 135.39 135.18 129.49 127.76 126.45 124.77 119.48 113.03 111.84 95.51 90.19 61.81 60.30 55.24 36.51 34.20 14.02
O
COOEt
O
3o
f1 (ppm)

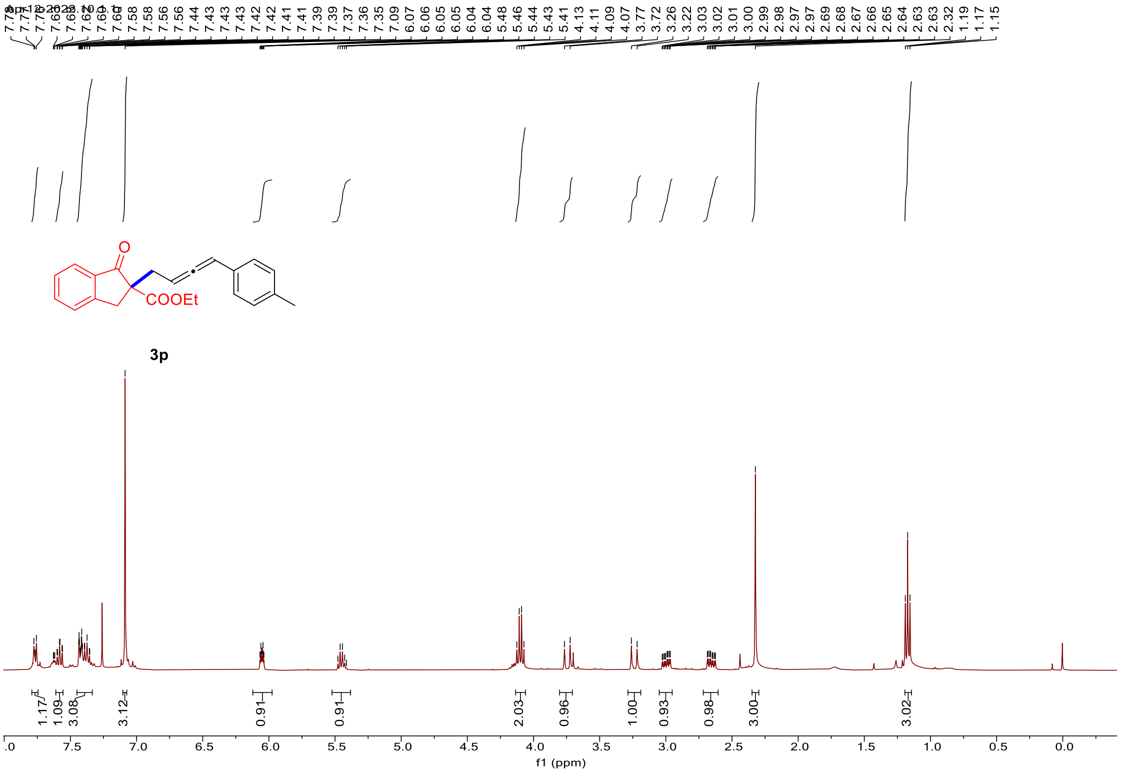
7.79
7.77
7.63
7.62
7.60
7.58
7.58
7.56
7.44
7.43
7.43
7.42
7.41
7.39
7.37
7.36
7.35
7.09
6.07
6.06
6.05
6.04
6.04
5.48
5.46
5.44
5.43
5.41
4.13
4.11
4.09
4.07
3.77
3.72
3.26
3.22
3.03
3.02
3.01
3.00
2.99
2.98
2.97
2.97
2.69
2.68
2.67
2.66
2.65
2.64
2.63
2.63
2.32
1.19
1.17
1.15
O
COOEt
3p
1.17
1.09
3.08
3.12
0.91
0.91
2.03
0.96
1.00
0.93
0.98
3.00
3.02
7.5
7.0
6.5
6.0
5.5
5.0
4.5
4.0
3.5
3.0
2.5
2.0
1.5
1.0
0.5
0.0
f1 (ppm)

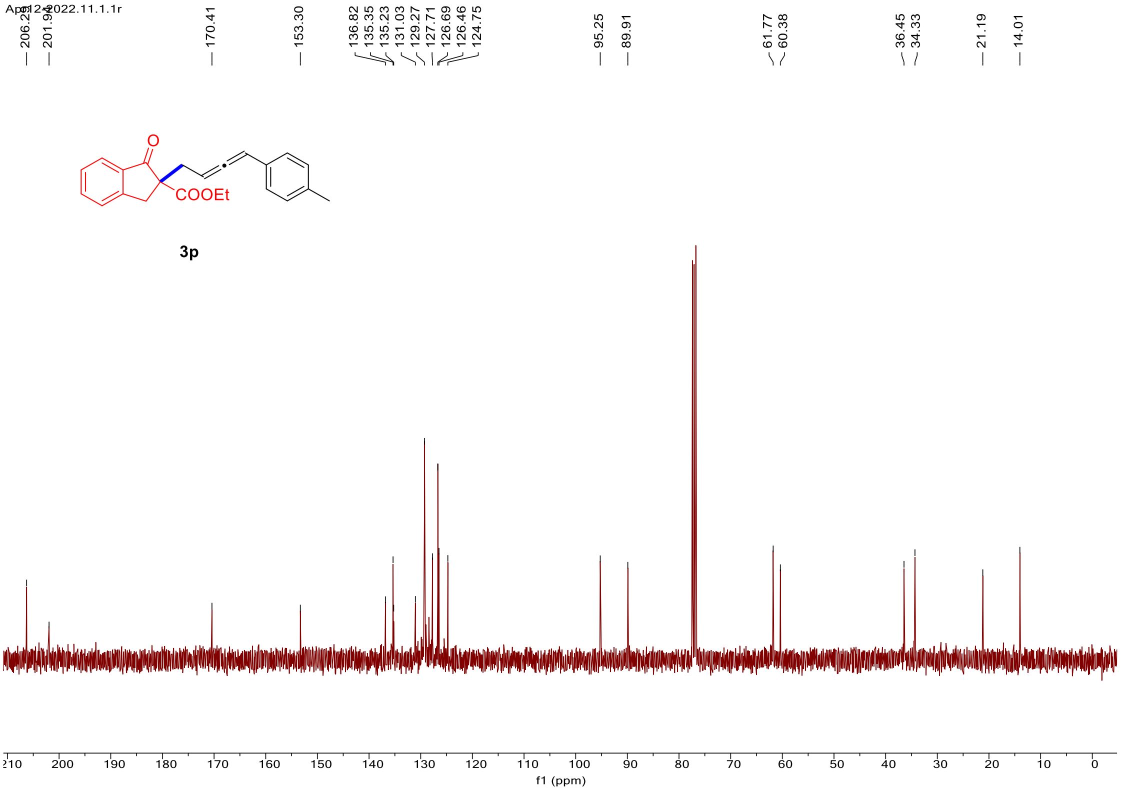
Apr12-2022.11.1.1r
206.21
201.96
170.41
153.30
136.82
135.35
135.23
131.03
129.27
127.71
126.69
126.46
124.75
95.25
89.91
61.77
60.38
36.45
34.33
21.19
14.01
O
COOEt
3p
200
190
180
170
160
150
140
130
120
110
100
90
80
70
60
50
40
30
20
10
0
f1 (ppm)

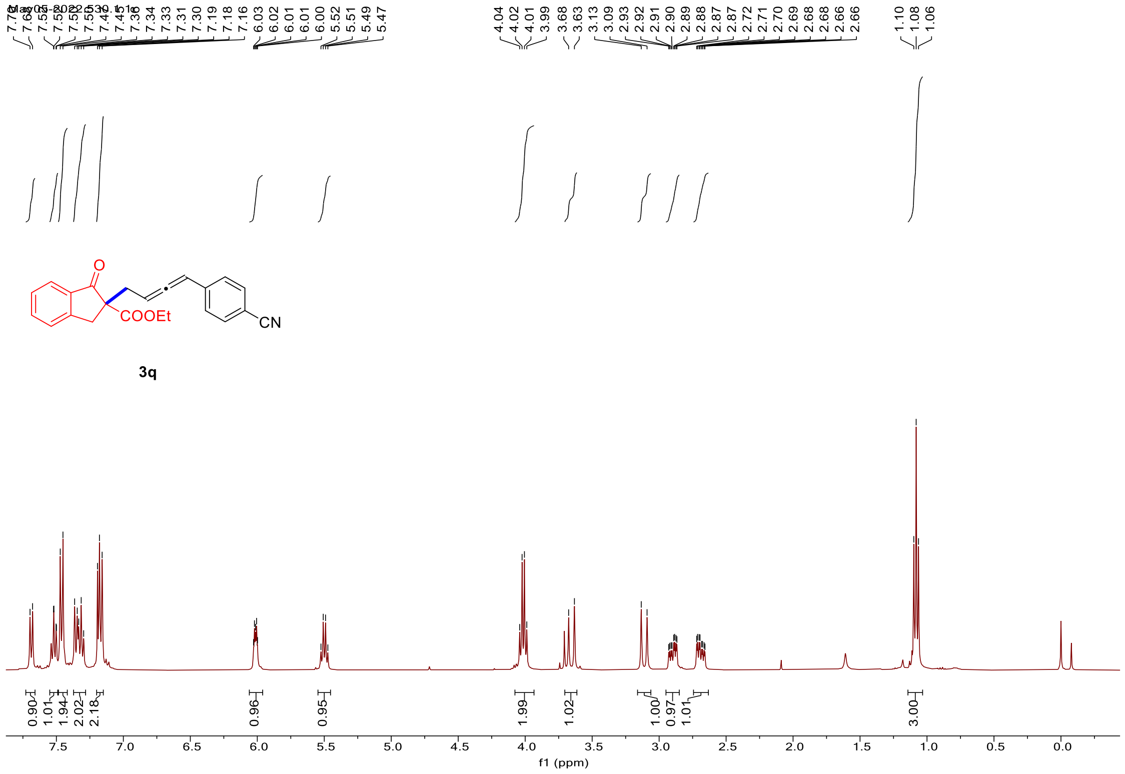
7.70
7.68
7.36
7.34
7.33
7.31
7.30
7.19
7.18
7.16
6.03
6.02
6.01
6.01
6.00
5.52
5.51
5.49
5.47
4.04
4.02
4.01
3.99
3.68
3.63
3.13
3.09
2.93
2.92
2.91
2.90
2.89
2.88
2.87
2.72
2.71
2.70
2.69
2.68
2.68
2.66
2.66
1.10
1.08
1.06
O
COOEt
CN
3q
0.90
1.01
1.94
2.02
2.18
0.96
0.95
1.99
1.02
1.00
0.97
1.01
3.00
f1 (ppm)

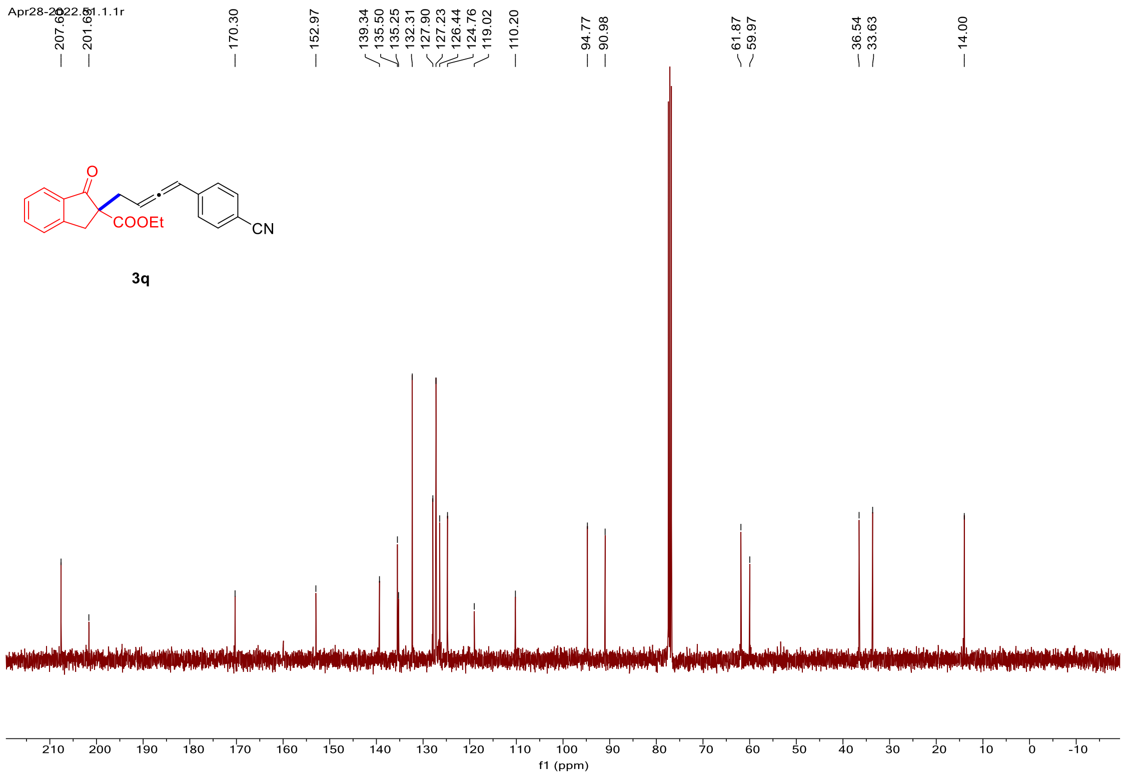
Apr28-2022.51.1.1r
207.60
201.69
170.30
152.97
139.34
135.50
135.25
132.31
127.90
127.23
126.44
124.76
119.02
110.20
94.77
90.98
61.87
59.97
36.54
33.63
14.00
O
COOEt
CN
3q
f1 (ppm)

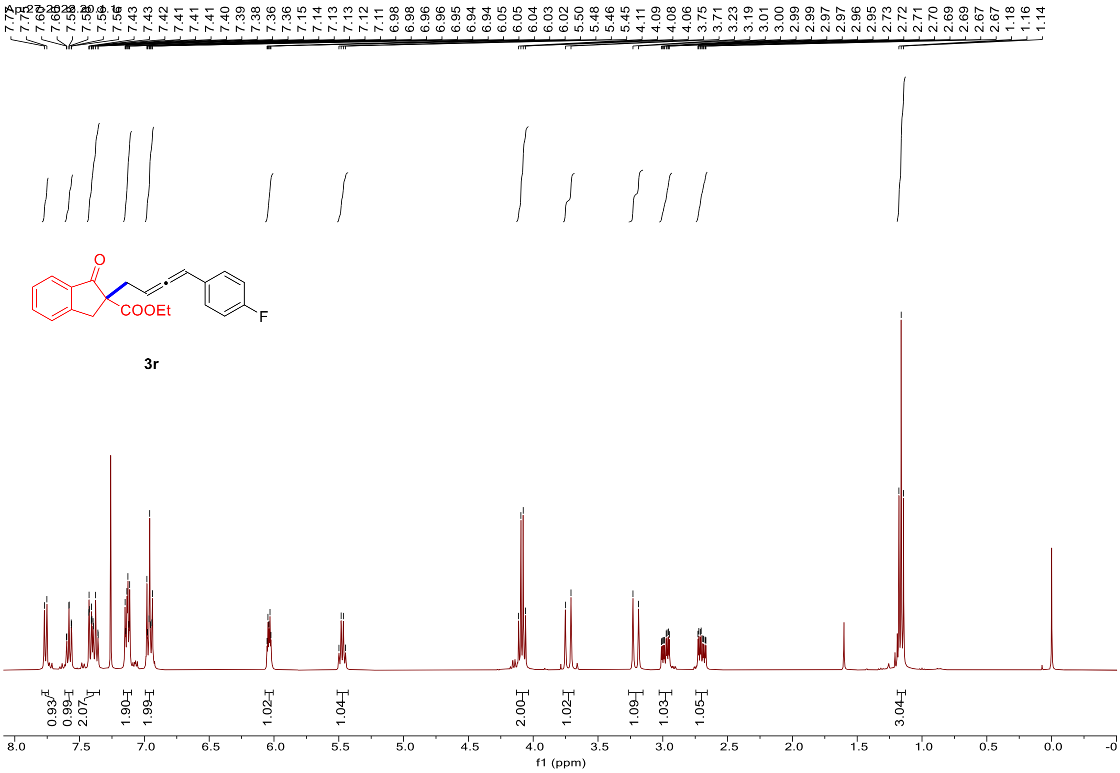
7.43 7.43 7.42 7.41 7.41 7.41 7.40 7.39 7.38 7.36 7.15 7.14 7.13 7.13 7.12 7.11 6.98 6.98 6.96 6.96 6.95 6.94 6.94 6.05 6.05 6.04 6.03 6.02 5.50 5.48 5.46 5.45 4.11 4.09 4.08 4.06 3.75 3.71 3.23 3.19 3.01 3.00 2.99 2.99 2.97 2.97 2.96 2.95 2.73 2.72 2.71 2.70 2.69 2.67 2.67 1.18 1.16 1.14
O
COOEt
F
3r
0.93
0.99
2.07
1.90
1.99
1.02
1.04
2.00
1.02
1.09
1.03
1.05
3.04
8.0 7.5 7.0 6.5 6.0 5.5 5.0 4.5 4.0 3.5 3.0 2.5 2.0 1.5 1.0 0.5 0.0
f1 (ppm)

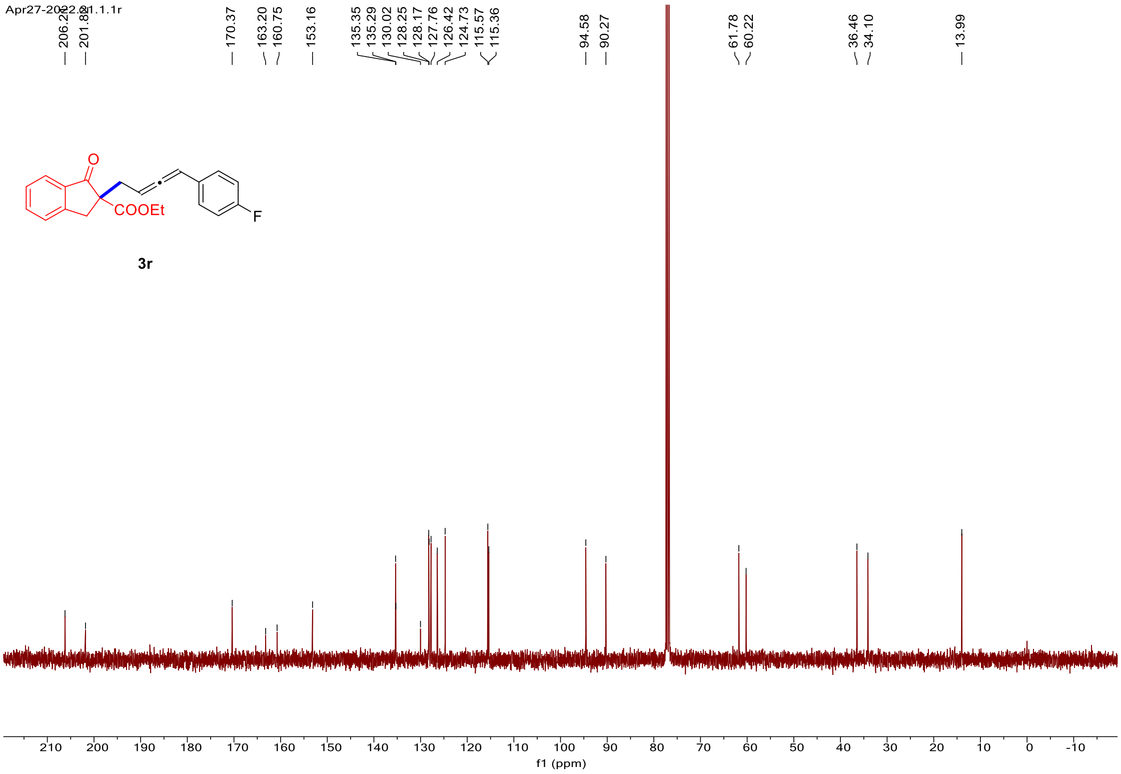
170.37 163.20 160.75 153.16 135.35 135.29 130.02 128.25 128.17 127.76 126.42 124.73 115.57 115.36 94.58 90.27 61.78 60.22 36.46 34.10 13.99
O
COOEt
F
3r
210 200 190 180 170 160 150 140 130 120 110 100 90 80 70 60 50 40 30 20 10 0 -10
f1 (ppm)

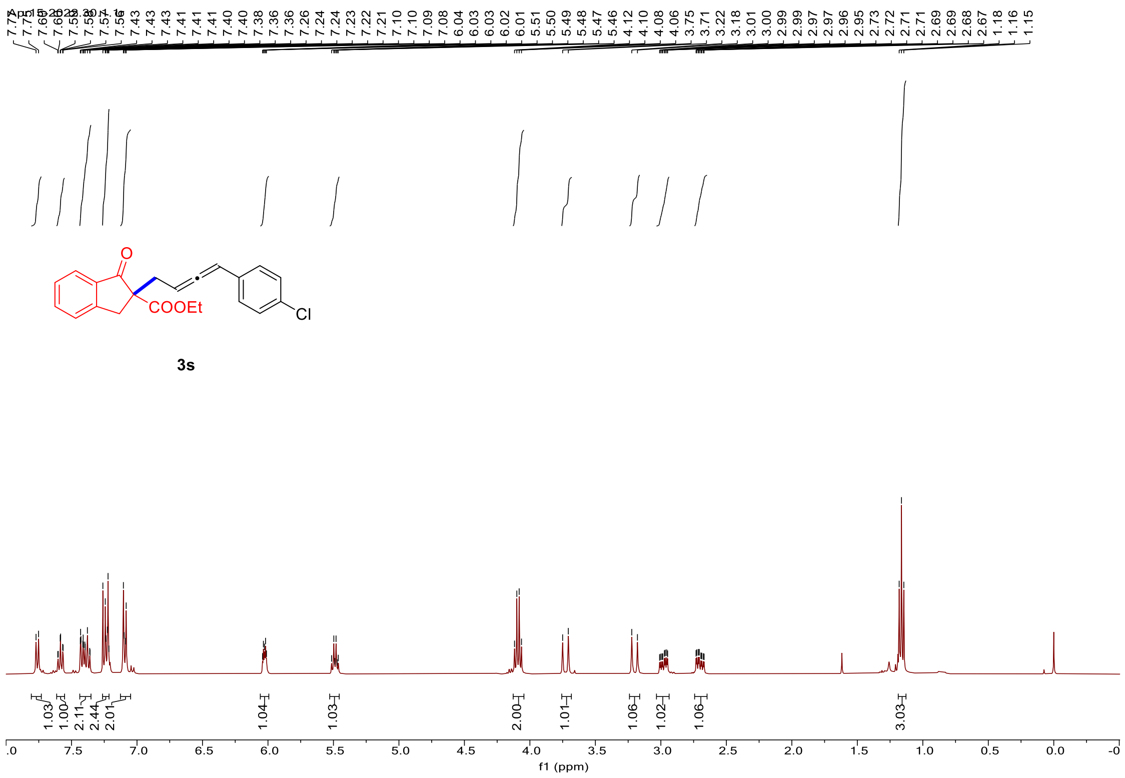
Apr15-2022.30.1.1r
7.77 7.75 7.61 7.60 7.58 7.56 7.54 7.43 7.43 7.41 7.41 7.40 7.40 7.38 7.36 7.36 7.26 7.24 7.24 7.23 7.22 7.21 7.10 7.10 7.09 7.08 6.04 6.03 6.03 6.02 6.01 5.51 5.50 5.49 5.48 5.47 5.46 4.12 4.10 4.08 4.06 3.75 3.71 3.22 3.18 3.01 3.00 2.99 2.99 2.97 2.97 2.96 2.95 2.73 2.72 2.71 2.71 2.69 2.69 2.68 2.67 1.18 1.16 1.15
O
COOEt
Cl
3s
1.03 1.00 2.11 2.44 2.01 1.04 1.03 2.00 1.01 1.06 1.02 1.06 3.03
f1 (ppm)

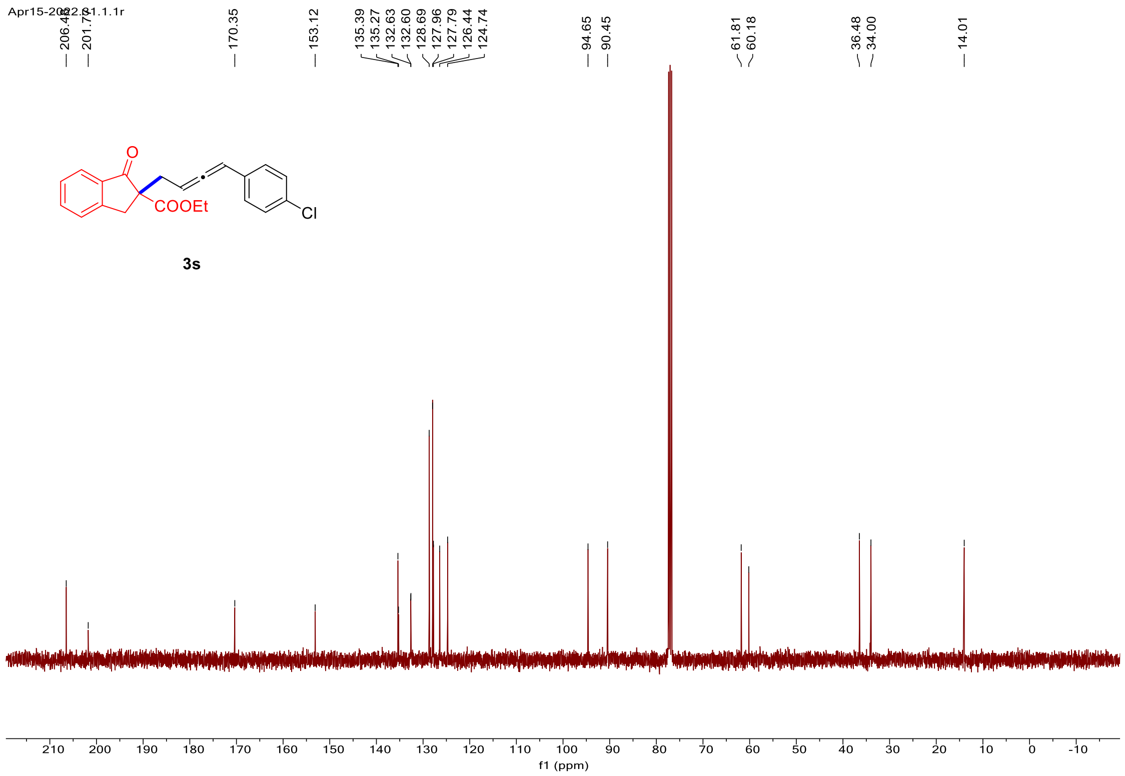
Apr15-2022.31.1.1r
206.42 201.77 170.35 153.12 135.39 135.27 132.63 132.60 128.69 127.96 127.79 126.44 124.74 94.65 90.45 61.81 60.18 36.48 34.00 14.01
O
COOEt
Cl
3s
f1 (ppm)

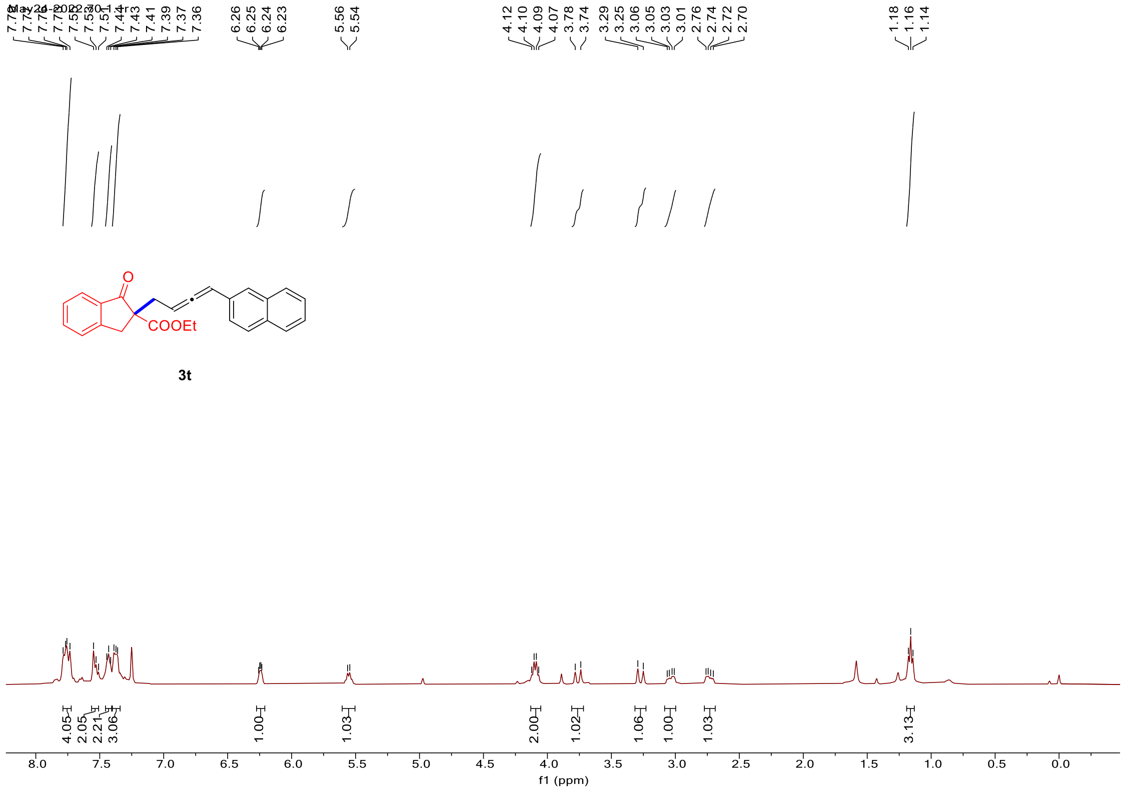
May24-2022.30.1.1r
7.79 7.77 7.74 7.72 7.70 7.52 7.53 7.51 7.44 7.43 7.41 7.39 7.37 7.36
6.26 6.25 6.24 6.23
5.56 5.54
4.12 4.10 4.09 4.07 3.78 3.74 3.29 3.25 3.06 3.05 3.03 3.01 2.76 2.74 2.72 2.70
1.18 1.16 1.14
O
COOEt
3t
4.05 2.05 2.21 3.06 1.00 1.03 2.00 1.02 1.06 1.00 1.03 3.13
f1 (ppm)

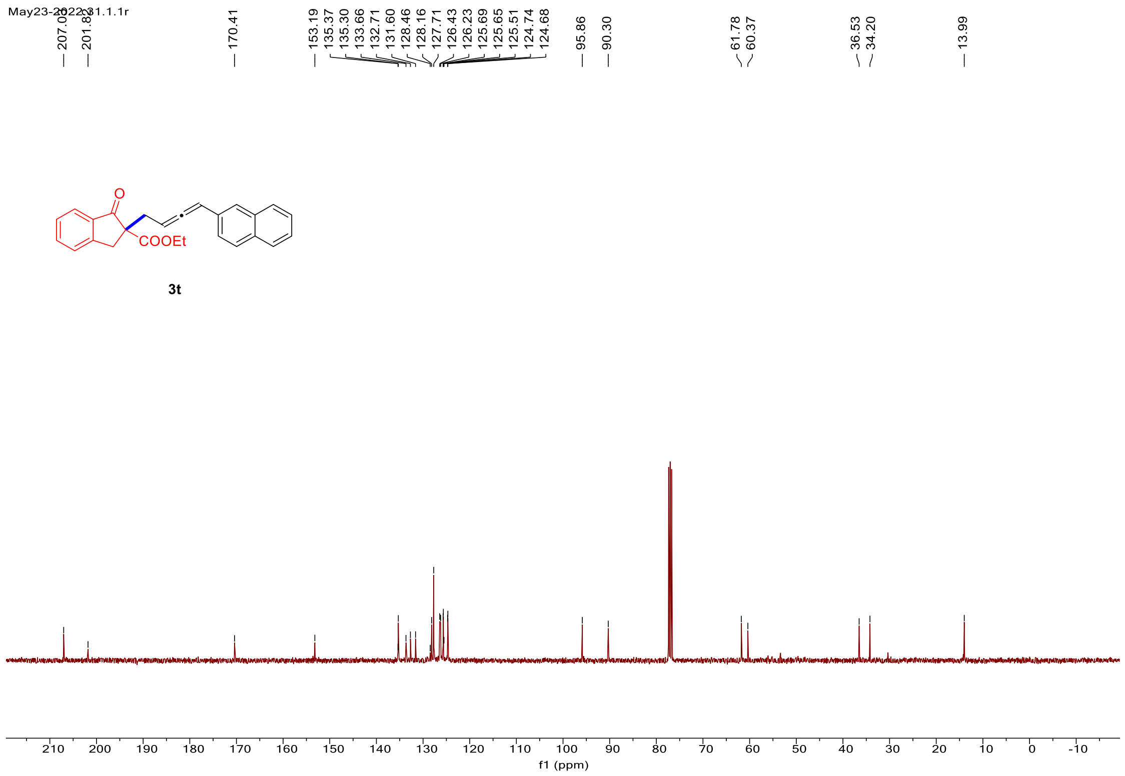
May23-2022.31.1.1r
207.03 201.82
170.41
153.19 135.37 135.30 133.66 132.71 131.60 128.46 128.16 127.71 126.43 126.23 125.69 125.65 125.51 124.74 124.68
95.86 90.30
61.78 60.37
36.53 34.20
13.99
O
COOEt
3t
f1 (ppm)

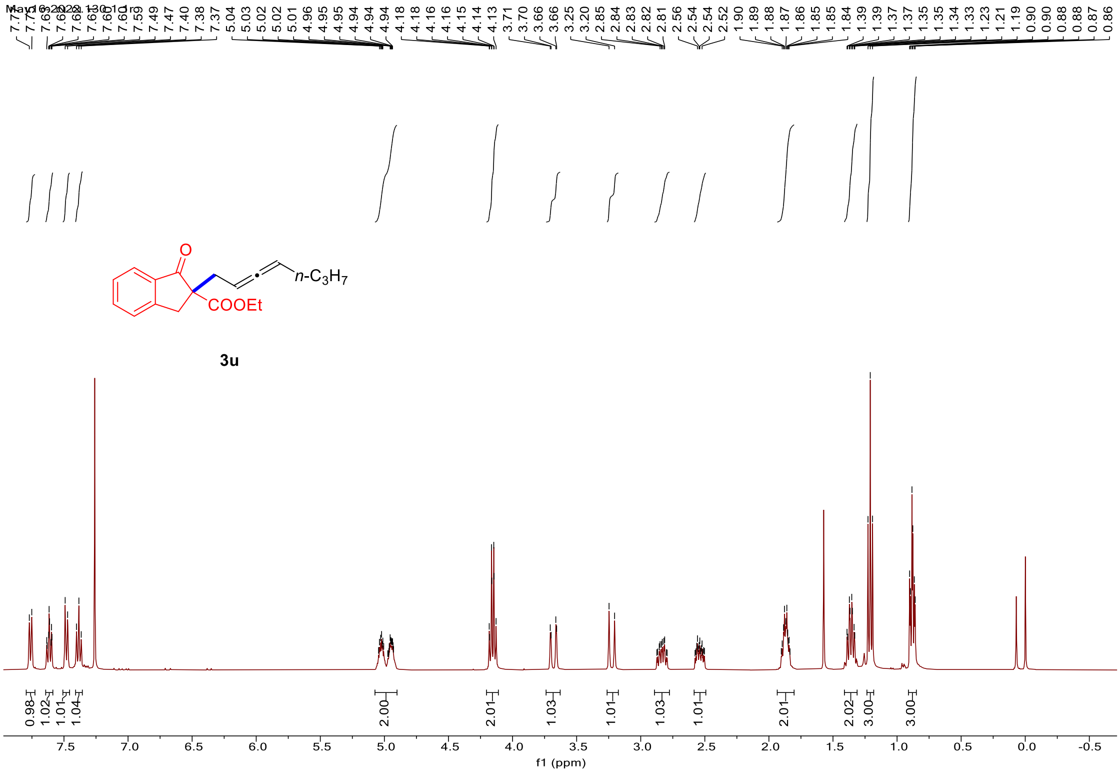
May16-2022.130.1.1r
COOEt
n-C3H7
3u
f1 (ppm)

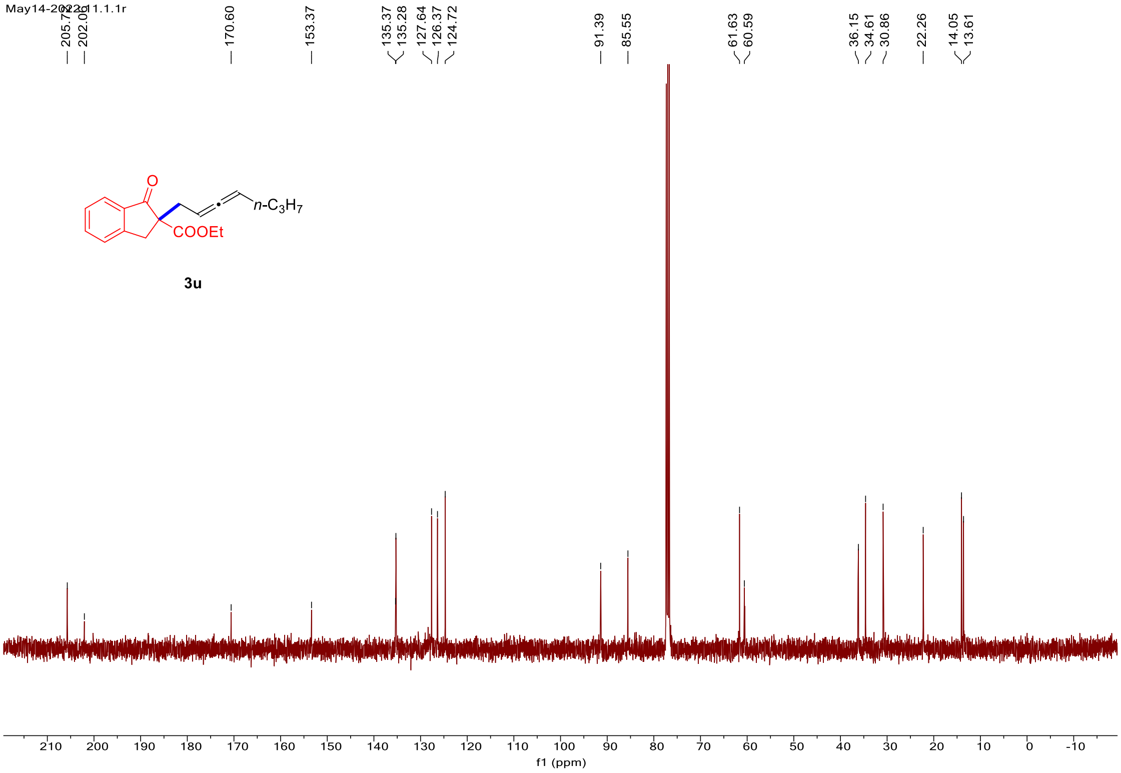
May14-2022c.11.1.1r
COOEt
n-C3H7
3u
f1 (ppm)

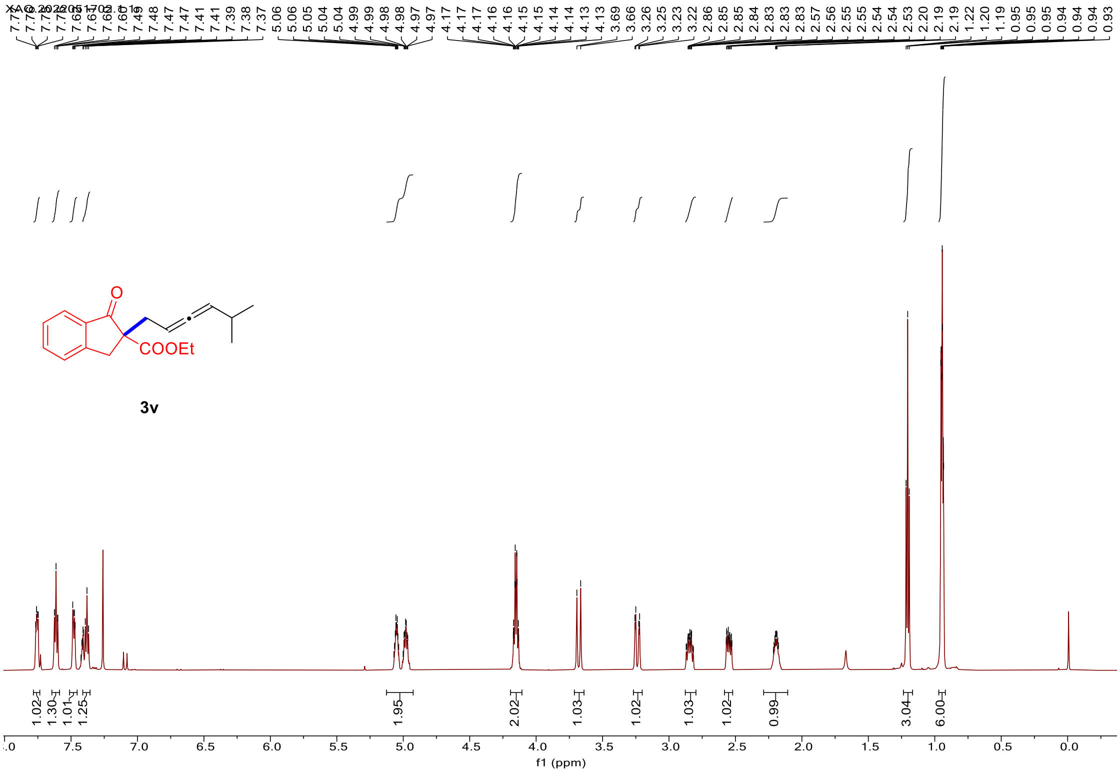
XAQ.2022051703.1.1r
7.49
7.48
7.47
7.47
7.41
7.39
7.38
7.37
5.06
5.06
5.05
5.04
5.04
4.99
4.99
4.98
4.98
4.97
4.97
4.17
4.17
4.16
4.16
4.15
4.15
4.14
4.14
4.13
4.13
3.69
3.66
3.26
3.25
3.23
3.22
2.86
2.85
2.85
2.84
2.83
2.83
2.57
2.56
2.55
2.55
2.54
2.54
2.53
2.20
2.19
2.19
1.22
1.20
1.19
0.95
0.95
0.94
0.94
0.94
0.94
0.93
COOEt
3v
1.02
1.30
1.01
1.25
1.95
2.02
1.03
1.02
1.03
1.02
0.99
3.04
6.00
f1 (ppm)

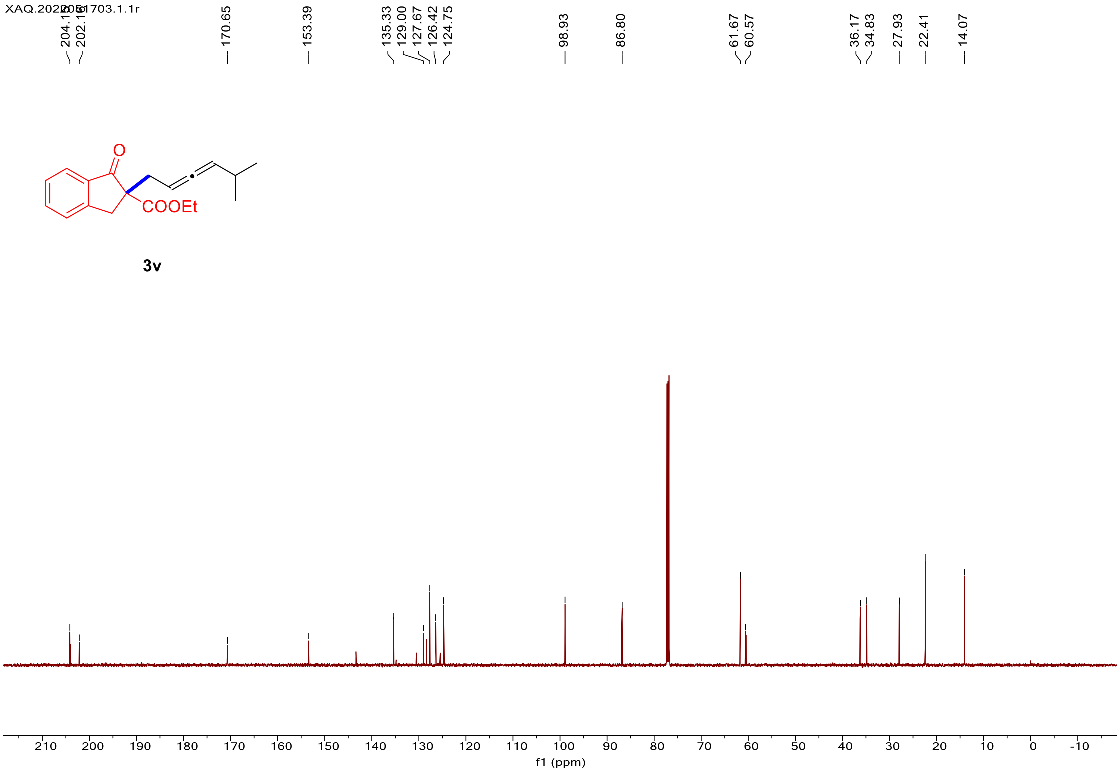
XAQ.2022051703.1.1r
204.15
202.15
170.65
153.39
135.33
129.00
127.67
126.42
124.75
98.93
86.80
61.67
60.57
36.17
34.83
27.93
22.41
14.07
COOEt
3v
f1 (ppm)

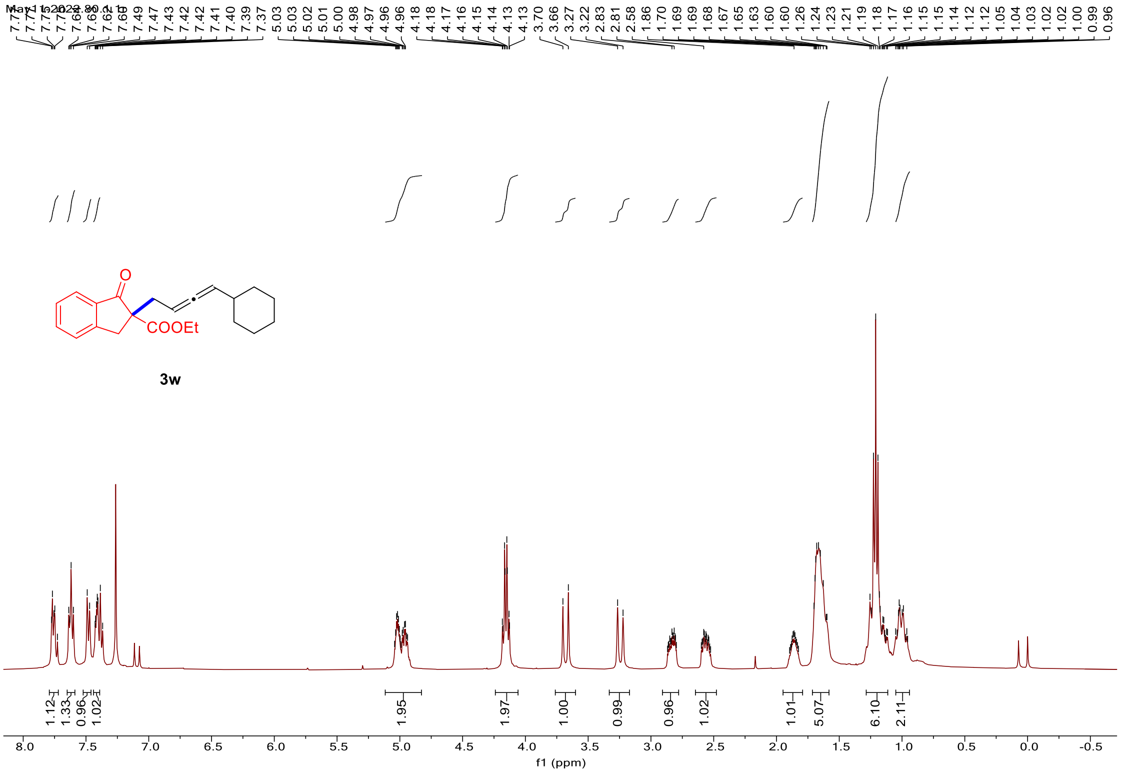
5.03
5.03
5.02
5.01
5.00
4.98
4.97
4.96
4.96
4.18
4.17
4.16
4.15
4.14
4.13
4.13
3.70
3.66
3.27
3.22
2.83
2.81
2.58
1.86
1.70
1.69
1.69
1.68
1.67
1.65
1.63
1.60
1.60
1.26
1.24
1.23
1.21
1.19
1.18
1.17
1.16
1.15
1.15
1.14
1.12
1.12
1.05
1.04
1.03
1.02
1.02
1.00
0.99
0.96
O
COOEt
3w
1.12
1.33
0.96
1.02
1.95
1.97
1.00
0.99
0.96
1.02
1.01
5.07
6.10
2.11
f1 (ppm)

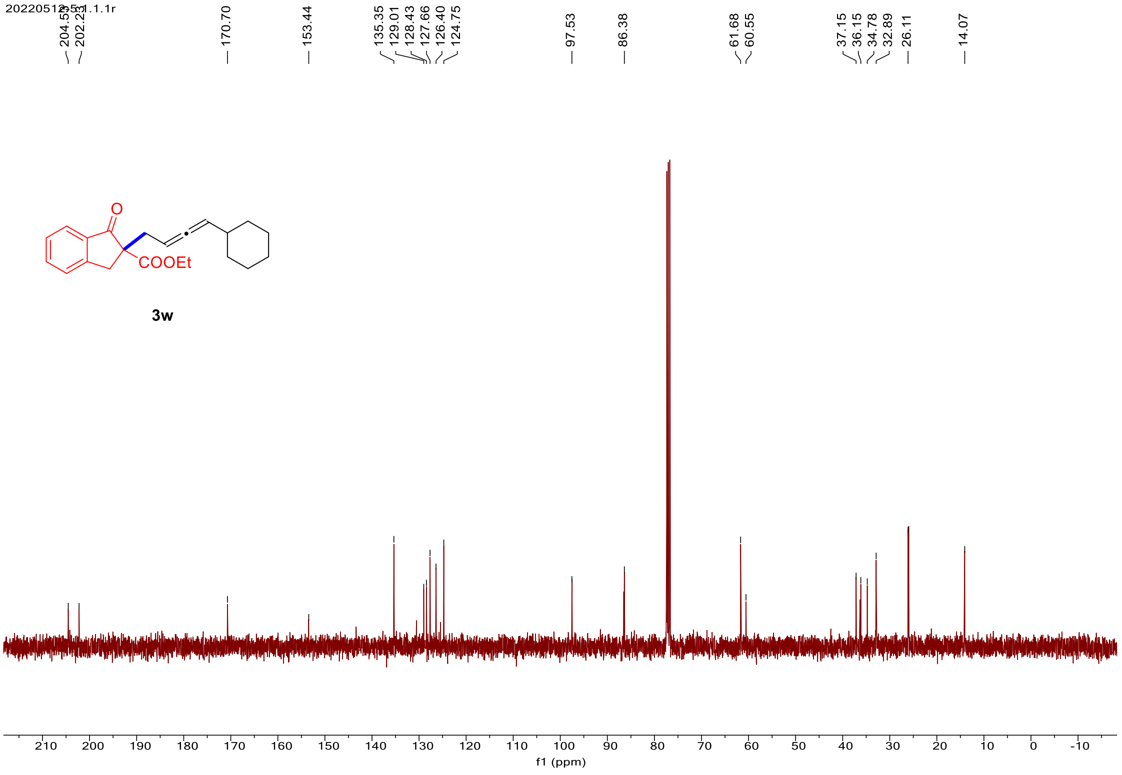
204.53
202.23
170.70
153.44
135.35
129.01
128.43
127.66
126.40
124.75
97.53
86.38
61.68
60.55
37.15
36.15
34.78
32.89
26.11
14.07
O
COOEt
3w
f1 (ppm)

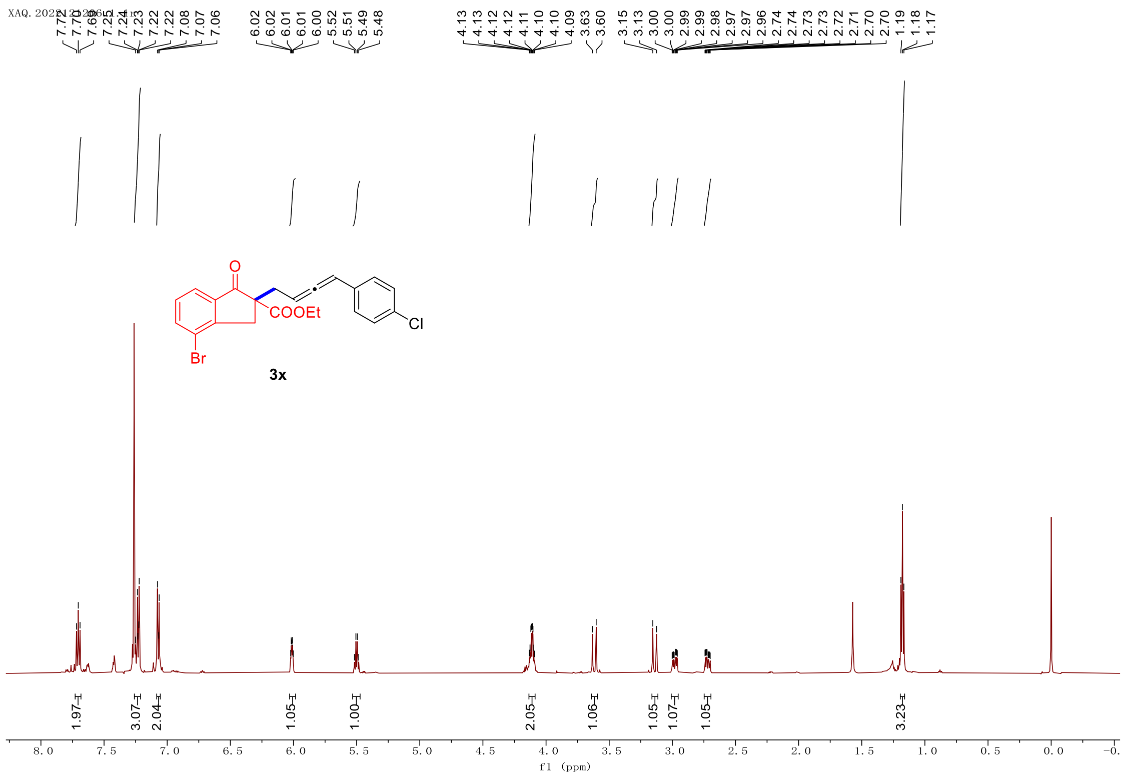

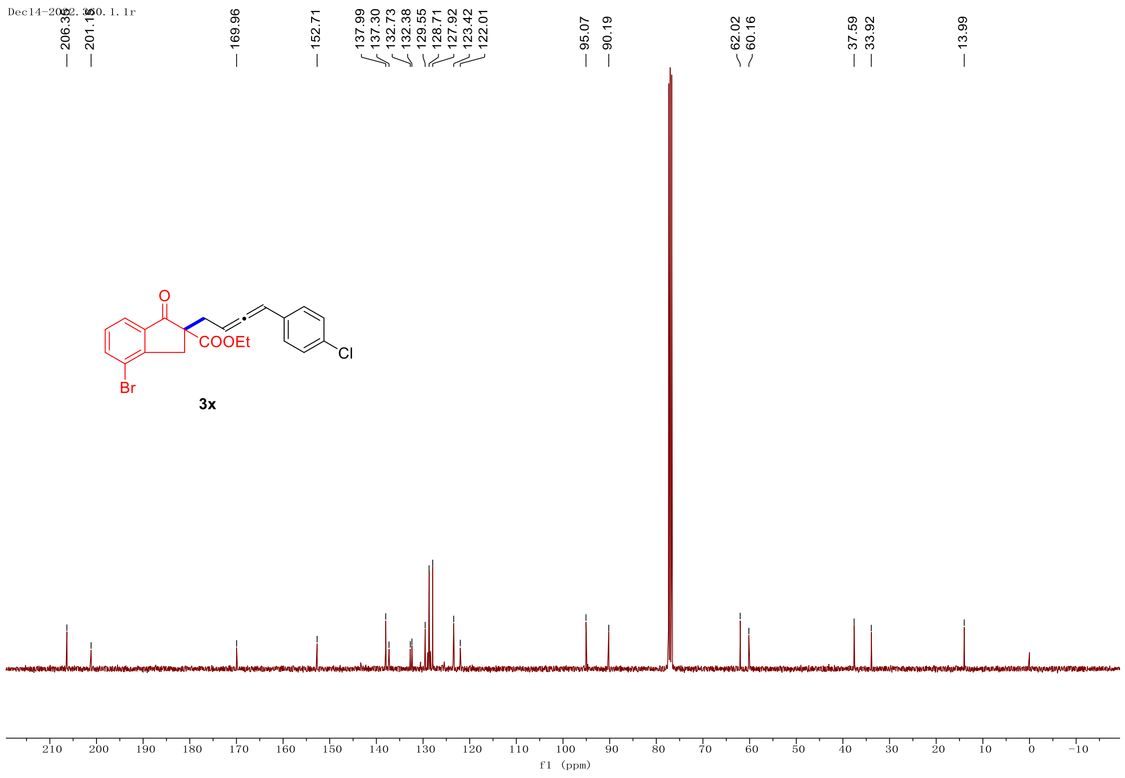

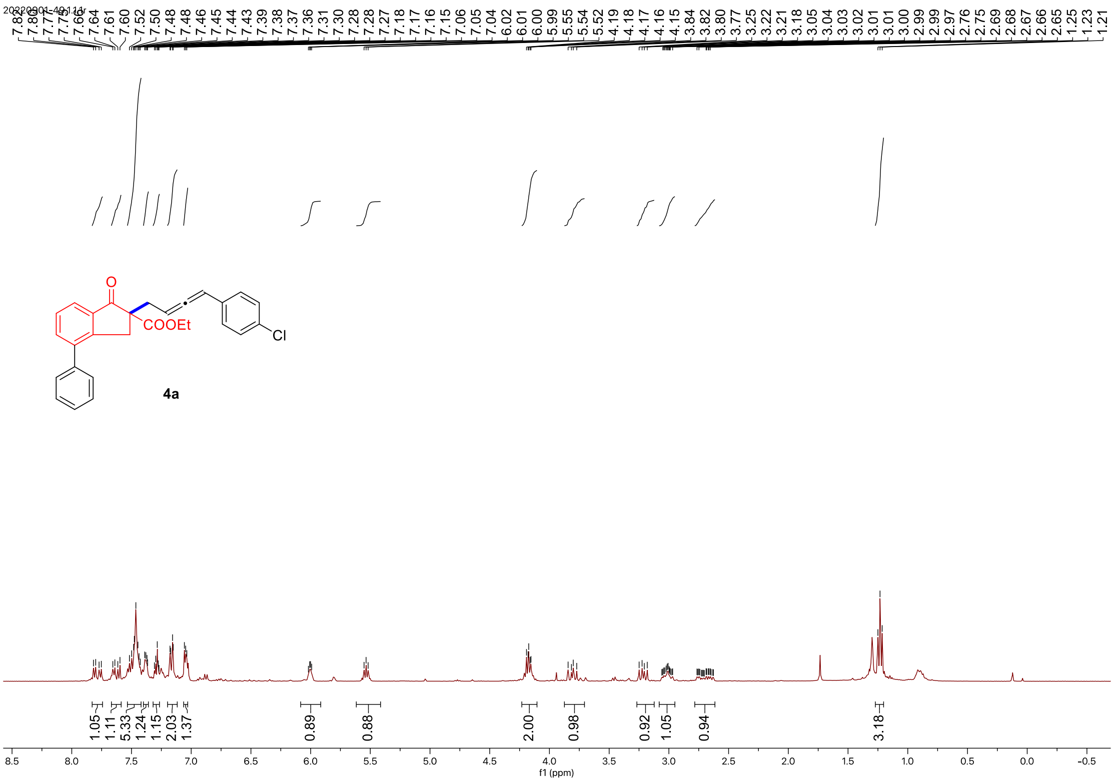
7.82 7.80 7.77 7.76 7.66 7.64 7.61 7.60 7.52 7.50 7.48 7.48 7.46 7.45 7.44 7.43 7.39 7.38 7.37 7.36 7.31 7.30 7.28 7.27 7.18 7.17 7.16 7.15 7.06 7.05 7.04 6.02 6.01 6.00 5.99 5.55 5.54 5.52 4.19 4.18 4.17 4.16 4.15 3.84 3.82 3.80 3.77 3.25 3.22 3.21 3.18 3.05 3.04 3.03 3.02 3.01 3.00 2.99 2.99 2.97 2.76 2.75 2.69 2.68 2.67 2.66 2.65 1.25 1.23 1.21
O
COOEt
Cl
4a
1.05 1.11 5.33 1.24 1.15 2.03 1.37 0.89 0.88 2.00 0.98 0.92 1.05 0.94 3.18
f1 (ppm)

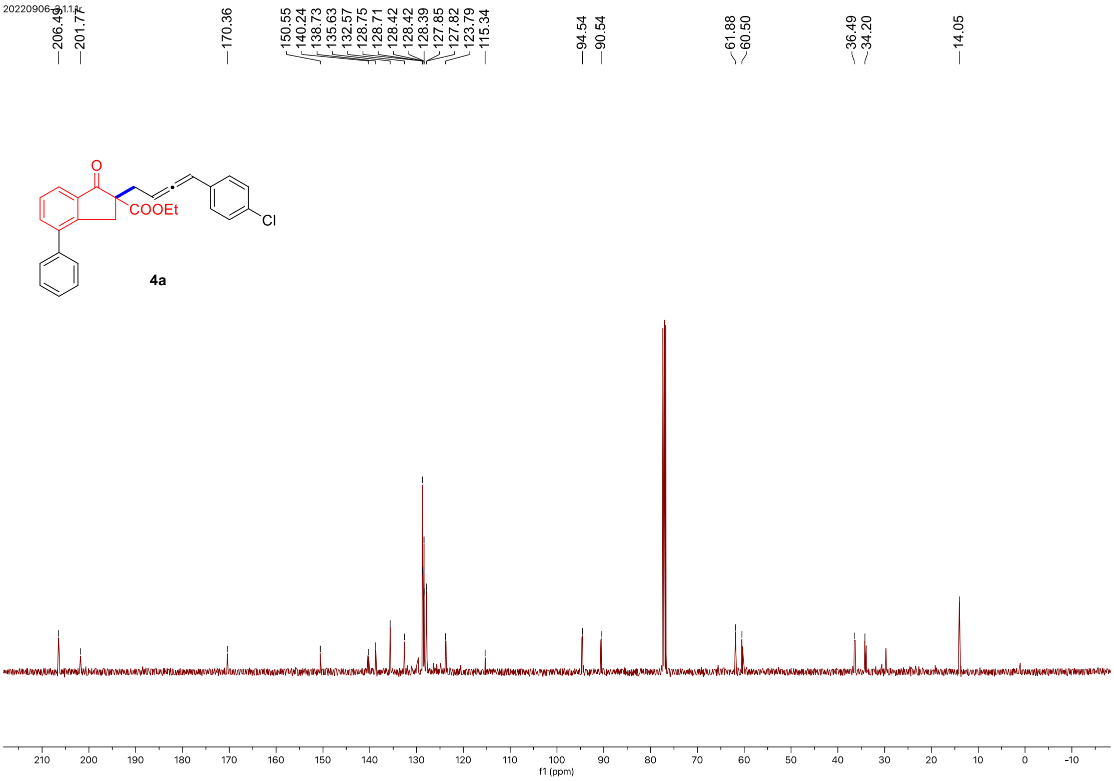
206.49 201.77 170.36 150.55 140.24 138.73 135.63 132.57 128.75 128.71 128.42 128.42 128.39 127.85 127.82 123.79 115.34 94.54 90.54 61.88 60.50 36.49 34.20 14.05
O
COOEt
Cl
4a
f1 (ppm)
